# Optimal Exercise Type and Dose for Blood Pressure Improvement in Middle-Aged and Older Adults with Type 2 Diabetes: A Systematic Review and Network Meta-Analysis

**DOI:** 10.3390/life16050843

**Published:** 2026-05-19

**Authors:** Bingwu Pang, Dongze Li, Kaiming Chen, Luguang Luo, Xinmiao Feng, Jiezhong Wu

**Affiliations:** 1 Department of Police Physical Education and Tactical Training, Railway Police College, Zhengzhou 450046, China; pangbingwu@rpc.edu.cn (B.P.); luoluguang@rpc.edu.cn (L.L.); 2 School of Sports Science and Engineering, East China Institute of Technology, Shanghai 330013, China; lidongze414@163.com; 3 Faculty of Sports Science, Ningbo University, Ningbo 315211, China; chenkm1017@163.com; 4 China Wushu School, Beijing Sport University, Beijing 100084, China; fengxinmiao666@163.com; 5 Department of Physical Education, Fuzhou University, Fuzhou 350108, China

**Keywords:** systolic blood pressure, diastolic blood pressure, middle-aged and older adults, exercise, meta-analysis, type 2 diabetes

## Abstract

Objective: This study assessed the blood-pressure effects of various exercise regimens in middle-aged and older adults with type 2 diabetes, considering baseline levels, to clarify dose–response relationships for personalized exercise guidance. Methods: We conducted a dose–response network meta-analysis. Systematic searches were performed in Web of Science, EMBASE, PubMed and Cochrane Library. Baseline blood pressure was modelled as an explanatory variable via meta-regression. Thirty-six eligible RCTs of physical-activity interventions in older adults with type 2 diabetes reporting blood-pressure outcomes were included. Results: In stage 1–2 hypertension, mind–body activities were associated with the greatest systolic blood-pressure reduction and appear to be the most effective intervention based on available evidence. For diastolic blood pressure, combined aerobic-resistance training was effective in stage 1 hypertension. Dose–response analysis indicated that clinically meaningful reductions occur at modest volumes (668 and 657 MET-min/week for systolic and diastolic pressures, respectively), aligning with the lower end of international activity guidelines. Conclusions: Individualized exercise prescription based on baseline blood pressure may offer a useful non-pharmacological strategy for hypertension management in older adults with type 2 diabetes. By quantifying the required activity dose, this work provides an evidence base for integrating structured exercise into precision care for this high-risk population.

## 1. Introduction

Type 2 diabetes mellitus (T2DM) is a chronic metabolic disorder of worldwide epidemic proportions [1], with its prevalence among adults continuing to rise sharply [2,3]. This trend is particularly pronounced in middle-aged and older adults [4], among whom the disease is not only more prevalent but also associated with a greater burden of complications [5], functional decline, and healthcare costs [6], constituting a critical health threat for this demographic [7].

Hypertension disproportionately affects middle-aged and older adults with diabetes relative to the general population [8], and these two conditions often coexist [9]. The concurrence of these chronic diseases exerts synergistic effects through multiple pathophysiological mechanisms, substantially elevating the risk of cardiovascular and cerebrovascular events, renal impairment, and other target organ damage [10]. Consequently, this leads to markedly increased rates of mortality and morbidity in middle-older patients. In the absence of a curative treatment, international guidelines, notably those from the American Diabetes Association (ADA), uniformly emphasize lifestyle modification as the cornerstone of disease management [11]. Within this framework, regular and structured physical activity plays a pivotal role in improving blood pressure (BP) control in middle-aged and older adults with T2DM [12,13].

Current guidelines widely recommend that individuals with type 2 diabetes should engage in at least 150 min of moderate-intensity aerobic exercise per week, supplemented with resistance training on two or more days, although more detailed, individualized recommendations for middle-aged and older adults have yet to be established [14]. Despite known BP benefits of activity, evidence on tailoring safe, effective exercise prescriptions for older adults with type 2 diabetes and hypertension, given their cardiovascular and health profiles, remains limited. Existing systematic reviews [15] have largely failed to delineate the effects of different exercise modalities (e.g., aerobic, resistance, mind–body activities) and have often overlooked the potential moderating influence of participants’ baseline BP levels and specific hypertension staging on intervention outcomes [16]. These factors are precisely the key considerations for ensuring the safety and efficacy of exercise interventions.

To address current limitations and advance individualized exercise prescription, this study systematically evaluates the effects of distinct exercise modalities on blood pressure control in middle-aged and older adults with type 2 diabetes and hypertension, with emphasis on baseline stratification and dose–response relationships, thereby providing targeted, clinically actionable evidence for precision guidance in this high-risk population.

## 2. Materials and Methods

### 2.1. Search Strategy

We performed a systematic review combined with a network meta-analysis. The results are reported in accordance with the Preferred Reporting Items for Systematic Reviews and Meta-Analyses (PRISMA) guidelines [17]. The protocol for this systematic review was preregistered with PROSPERO (International Prospective Register of Systematic Reviews) under the identifier CRD420251266429. We conducted a systematic search across several major bibliographic databases, such as PubMed, EMBASE, Web of Science, and the Cochrane Library. The literature search was constructed using a combination of indexed subject headings and keywords. The full syntax is available in Supplementary Material S1 and is registered with PROSPERO. To ensure exhaustive coverage of relevant literature, supplemental manual screening of references from eligible studies and related reviews was performed.

### 2.2. Selection Criteria

Inclusion required that studies fulfill all criteria below: (1) randomized controlled trials; (2) individuals with a diagnosis of type 2 diabetes, as defined by American Diabetes Association criteria [18], with a mean age ≥ 45 years (i.e., middle-aged and older adults); (3) a structured exercise regimen of any modality delivered as the intervention; (4) a comparator arm assigned to either usual care or a distinct physical activity regimen; and (5) reporting of blood pressure-related outcomes. We applied the following exclusion criteria: (1) Trials employing multi-component interventions (e.g., physical activity combined with dietary change) from which the specific effect of exercise could not be isolated. (2) Studies involving participants with serious comorbidities—such as advanced heart failure, major recent cardiovascular events, or uncontrolled psychiatric disorders—deemed likely to compromise safety, protocol adherence, or the validity of the findings. (3) Investigations examining only short-term effects (intervention duration < 4 weeks).

### 2.3. Data Extraction

Following Cochrane guidelines, independent data extraction was conducted by three members of the research team [19]. The extracted information encompassed general study characteristics, participant demographics, and details of the physical activity intervention. Data regarding control groups and key outcomes were also collected. Where available, statistical data necessary for calculating change scores were extracted to facilitate meta-analysis. To ensure completeness, requests for missing essential data were submitted directly to the studies’ corresponding authors.

Where studies provided only the standard error (*SE*) of the mean, values were transformed to the standard deviation (*SD*) based on the procedure outlined in the Cochrane Handbook for Systematic Reviews (1).

(1) $$\mathrm{SD}=\mathrm{SE}\times \sqrt{N}$$

For studies reporting only baseline (*M_pre_*, *SD_pre_*) and post-intervention (*M_pos_*_t_, *SD_pre_*) data, the intervention-induced change from baseline was calculated (2).

(2) $$M_{change}=M_{post}-M_{pre}$$

The following formula was used to calculate the standard deviation of change scores (*SD_change_*): In the absence of reported coefficients from the original studies, a conservative estimate of *R* = 0.5 was adopted (3).

(3) $${SD}_{change}=\sqrt{{SD}_{pre}^{2}+{SD}_{post}^{2}-(2\times R\times {SD}_{pre}\times {SD}_{post})}$$

### 2.4. Data Analysis

Referencing the published reports, two reviewers independently coded the interventions. This coding adhered to both the ACSM classification [20] and the exercise details provided in each study. The modalities included: aerobic exercise, resistance training, combined aerobic and resistance training, high-intensity interval training (HIIT), mind–body exercise, and walking. Given its distinct low mechanical load with concurrent vascular occlusion, blood flow restriction exercise (BFRE) elicits greater metabolic stress and differential sympathetic activation compared with conventional high-load resistance training. These unique physiological responses may exert distinct blood pressure effects [21], justifying its separate classification and independent analysis in this network meta-analysis.

This analysis focused on changes in systolic and diastolic blood pressure (SBP, DBP), with all values reported in millimetres of mercury (mmHg). The intervention effect was quantified as the pooled mean change from baseline. We computed these effect sizes and corresponding standard deviations using reported pre- and post-intervention values (means, SDs, sample sizes) per arm, following established Cochrane guidelines [19].

We evaluated two alternative meta-regression frameworks, namely fixed-effects and random-effects models. All models included baseline blood pressure as a covariate to inform the selection of the optimal synthesis approach [19]. Model selection was based on goodness-of-fit statistics, including residual deviance, number of parameters, and the Deviance Information Criterion (DIC), which penalizes model complexity. The model yielding the lowest DIC value was chosen to report the primary intervention effect estimates. These analyses [22], which effectively constitute an analysis of covariance (ANCOVA) within the meta-analytic framework, therefore provided the adjusted effect estimate and its associated standard error. The analyses were implemented within a Bayesian framework to integrate relevant prior evidence. A weakly informative normal prior (N (0, scale = 10)) was set for treatment effects, reflecting the expected range of blood pressure reduction from physical activity in this cohort [23]. Model choice relied on residual deviance, parameter count, and Deviance Information Criterion (DIC); the lowest DIC model provided adjusted effect estimates and standard errors via meta-analytic analysis of covariance (ANCOVA) [24]. Model comparison based on residual deviance, Deviance Information Criterion (DIC), and τ estimates identified the random-effects covariate model as optimal (lowest DIC), balancing fit and complexity. Global consistency was assessed via consistency versus unrelated mean effects (UME) model comparison and deviance-deviance plots [25].

Based on the chosen model, we calculated effect sizes versus usual care and generated predicted blood pressure reductions across a range of baseline levels. These baseline levels were set at the mean values for the categorical ranges established by the American Heart Association [26]. Control groups within the trials we analyzed consisted of participants who either received usual care or were assigned to a different mode of exercise as the comparator intervention. In this network meta-analysis, usual care formed the shared reference treatment against which all interventions were evaluated. Concurrently, study arms involving alternative physical activity regimens served as active controls, enabling direct contrast between different exercise modalities. All varying control conditions were incorporated into a single analytical model. The Bayesian network meta-analysis approach directly handles these comparative structures, rendering separate subgroup analyses for this variable unnecessary.

An average reduction in systolic blood pressure of at least 5 mmHg was considered a clinically meaningful change [27]. Given the absence of a validated minimal clinically important difference for diastolic blood pressure, this study pragmatically defined the threshold for clinical significance based on a change in standard hypertension classification (Table 1).

This criterion carries direct clinical relevance: a downgrade in blood pressure category is closely associated with a graded reduction in cardiovascular event risk, and such reclassification frequently informs clinical decision-making regarding the initiation or intensification of antihypertensive therapy. For example, a reduction in diastolic blood pressure to below 90 mmHg, resulting in reclassification from stage 2 to stage 1 hypertension, was considered a clinically meaningful improvement [28]. To visualize the impact of baseline blood pressure, we constructed a regression plot showing the fitted line and its confidence band. To further compare the interventions, we calculated posterior treatment rankings and their corresponding probabilities for different baseline levels, thereby identifying the physical activity type most likely to be effective at each specific stage [29].

Evidence indicates that in the presence of heterogeneity, decision-makers should additionally consider the predictive distribution of relative effects in a new, future study, as this reflects the uncertainty associated with the rollout of a treatment in practice [30]. Accordingly, we computed the predictive probabilities for a null or adverse blood pressure outcome in a hypothetical new trial, which would assess a specific exercise modality among participants across a range of baseline blood pressure levels.

### 2.5. Analysis of Dose–Response Relationships in Calculated Physical Activity

This study calculated weekly activity dose (MET-minutes) by multiplying the prescribed activity’s MET coefficient by minutes per session and sessions per week, summing across components for multi-intervention protocols. When trials reported specific details (e.g., speed, modality), we assigned MET values using standard compendia [31]. For studies reporting only intensity category (light, moderate, vigorous), we applied ACSM’s absolute thresholds [32]: light 2.0–2.9, moderate 3.0–5.9, vigorous ≥ 6.0 METs, using the midpoint (e.g., moderate = 4.5). For multi-component sessions, we calculated each component’s dose separately and then aggregated. In the absence of metabolic data, we operationally coded high-intensity interval training (HIIT) as vigorous exercise unless workload data allowed precise MET estimation. Resistance training intensity followed ACSM’s %1RM criteria (light < 50%, moderate 50–69%, vigorous 70–85%); when %1RM was unreported, we derived intensity from perceived exertion (RPE: moderate 12–13, vigorous 14–17) or repetition ranges (e.g., 6–8 reps). For dose estimation, we adopted standard MET values from the Compendium of Physical Activities: 3.5 (light/moderate), 6.0 (vigorous), and 8.0 (circuit training). When published reports lacked complete information or the minimum data needed for dose–response modelling, we contacted corresponding authors to request supplementary data.

The final analysis focused on the relationship between varying weekly physical activity volumes (MET-min/week) and corresponding changes in systolic and diastolic blood pressure. Both linear and non-linear regression models were fitted to the data. Linear association was assessed via ordinary least squares regression, while non-linear patterns were explored by fitting natural spline models with 2 and 3 knots, implemented in line with Harrell’s flexible modeling strategy [33]. The comparison of nested models via F-tests revealed a consistent pattern for both systolic and diastolic blood pressure [34]. For systolic blood pressure, both two- and three-knot spline models significantly outperformed the linear model (*p* = 4.31 × 10^−5^ and *p* = 1.82 × 10^−4^, respectively); diastolic findings were similar (*p* = 3.75 × 10^−5^ and *p* = 1.58 × 10^−4^). The two-knot spline demonstrated superior statistical efficiency, achieving fit improvement with one additional parameter (Δdf = 1; SBP: F = 18.85; DBP: F = 19.50) versus the three-knot model requiring two (Δdf = 2; SBP: F = 9.68; DBP: F = 9.89). Upholding parsimony and mitigating overfitting, the two-knot natural spline was selected as the optimal interpretable form for dose–response characterisation. The optimal weekly dose was defined as the volume minimising predicted blood pressure. Nonlinear models were applied only where ≥5 independent estimates spanned ≥4 dose levels. Confidence intervals quantified uncertainty along predicted curves. The World Health Organization recommended activity range (600–1200 MET-min/week) is annotated in the figure for interpretation [35].

All analyses were conducted in R (version 4.5.2). Bayesian network meta-analyses and meta-regression were performed using the multinma package with a Stan backend [36]. Dose–response modelling via linear and natural spline regression was implemented using the stats and splines packages, and all data visualization was produced with ggplot2 [37].

### 2.6. Assessment of Study Quality and Publication Bias

The Cochrane Risk of Bias tool (RoB 2) was applied to assess the methodological quality of all included trials [38]. Funnel plot inspection (for asymmetry) and Egger’s linear regression test served to evaluate the potential for publication bias [39].

## 3. Results

**Figure 1:**
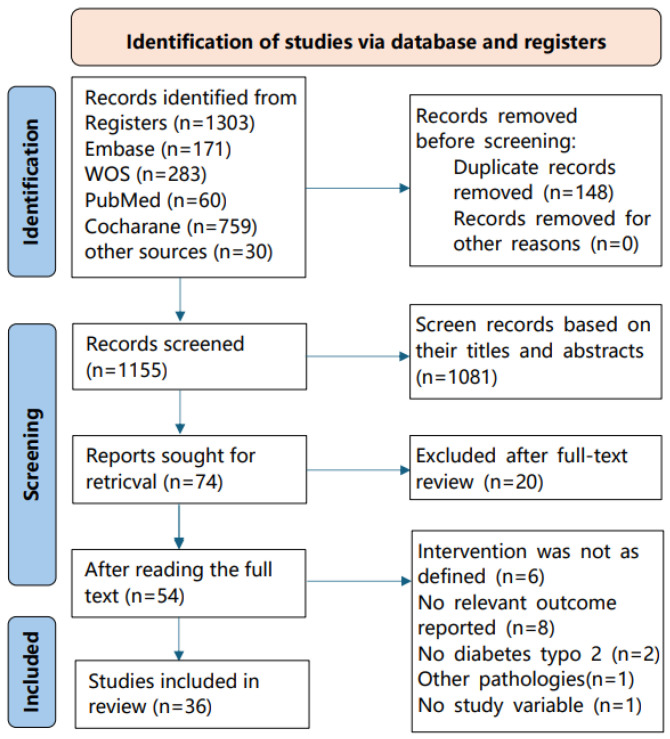
Flow chart illustrating the selection of studies according to PRISMA guidelines.

The systematic literature search identified 1303 potentially eligible articles. After the removal of duplicates, 1155 articles were screened based on titles and abstracts. Of these articles, 74 met the criteria for a full-text review. After a detailed assessment, 36 studies were included in the final systematic review and meta-analysis. The complete study selection process is depicted in Figure 1. Detailed characteristics of the included studies are provided in Supplementary S2.

### 3.1. Network Geometry

These are based on data from the 36 included studies. Our analysis revealed 45 and 44 direct comparisons in the evidence networks for SBP and DBP, respectively (Supplementary S5). The connectivity within each network allowed for meaningful direct and indirect comparisons, thereby satisfying the core connectivity assumption required for a valid network meta-analysis [40].

Comparison of models based on the DIC indicated that for both SBP and DBP outcomes, the random-effect model incorporating the covariate (DIC = 123.9 for SBP and 128.2 for DBP) was favored over the corresponding unrelated mean effects model (DIC = 126.1 for SBP and 132.7 for DBP). The absence of detectable inconsistency in these analyses supports the underlying consistency assumption of our network meta-analysis (Supplementary S6). These findings indicate an agreement between direct and indirect evidence. However, network sparsity (e.g., few trials for BFRE) warrants caution for comparisons relying on indirect evidence.

### 3.2. Network Meta-Analysis

#### 3.2.1. SBP

**Figure 2:**
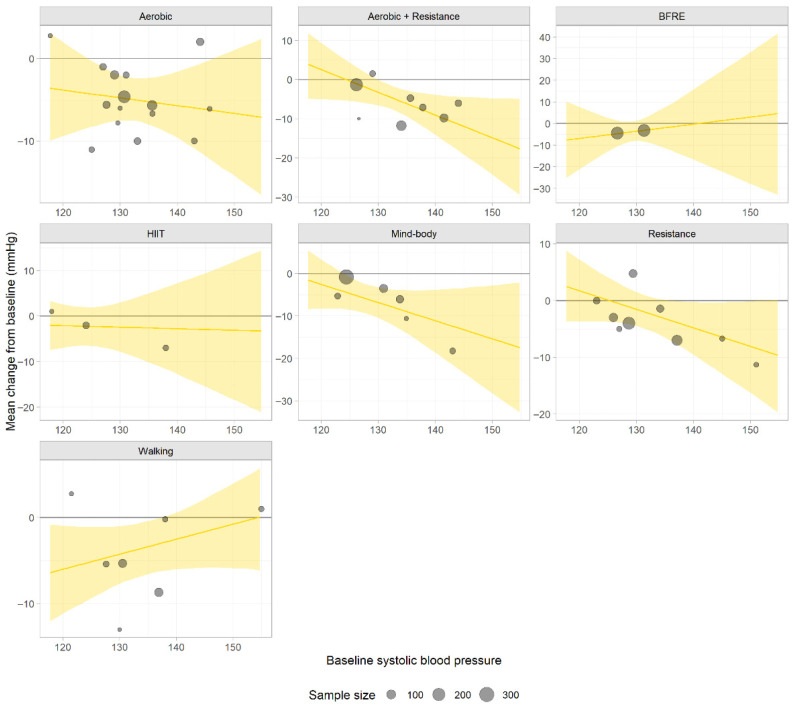
The meta-regression lines depict treatment-specific effects on systolic blood pressure. The sample size of each study determines the area of its corresponding data point in the figure.

In individuals with a baseline SBP of 125 mmHg (elevated BP), walking was the only intervention that resulted in a statistically significant and clinically meaningful reduction in BP (CFB = −5.13; 95% CrI, −9.37 to −1.08). Mind–body activities resulted in a statistically significant reduction (CFB = −4.69; 95% CrI, −8.52 to −0.79); however, the magnitude of the reduction was not clinically meaningful. In patients with stage 1 hypertension (baseline SBP, 135 mmHg), three interventions yielded both statistically significant and clinically meaningful effects: mind–body activities yielded the most pronounced effect (CFB = −9.00; 95% CrI, −14.06 to −3.89), followed by combined aerobic and resistance training (CFB = −6.15; 95% CrI, −9.41 to −2.78) and aerobic exercise (CFB = −5.27; 95% CrI, −8.00 to −2.34). In patients with stage 2 hypertension (baseline SBP, 145 mmHg), the number of effective interventions and the magnitude of BP reduction increased further. Mind–body activities (CFB = −13.27; 95% CrI, −23.39 to −3.46) and combined aerobic and resistance training (CFB = −12.02; 95% CrI, −19.21 to −4.45) showed the strongest effects. In addition, resistance training (CFB = −6.43; 95% CrI, −12.89 to −0.32) and aerobic exercise (CFB = −6.18; 95% CrI, −11.81 to −0.38) showed statistically significant and clinically meaningful reductions (Supplementary S7). The meta-regression lines are shown in Figure 2.

Based on available data, in individuals with a baseline SBP of 125 mmHg (elevated BP), walking ranked the highest among all interventions (rank value = 2.60). In patients with stage 1 and stage 2 hypertension, mind–body activities showed a ranking advantage, with rank values of 1.52 and 1.92, respectively, exhibiting the highest relative probability of being the optimal treatment option (Supplementary S11).

Analysis of ranking probabilities suggested that in individuals with high-normal baseline SBP (125 mmHg), BFRE showed the highest estimated probability of being the optimal treatment option (39%), followed by walking (25%). The cumulative top-three probabilities for these interventions reached 65% and 76%, respectively, indicating a possible early advantage with remaining uncertainty. In patients with stage 1 hypertension, mind–body activities showed a high probability of being the optimal intervention (70%) and an 87% cumulative top-two probability, suggesting favorable applicability. Combined aerobic and resistance training showed notable performance (51% second-best probability), indicating that the combined use of these interventions is a priority consideration under current evidence. In patients with stage 2 hypertension, mind–body activities showed the highest probability of being the optimal intervention (49%), followed by combined aerobic and resistance training (35%). Their cumulative top-two probabilities (78% and 74%, respectively) indicate promising outcomes; however, the relative magnitude of the advantage remains somewhat uncertain (Supplementary S13).

Analysis of predictive probabilities revealed that the risk of a future trial showing no effect or adverse effects substantially differed across exercise interventions and was closely related to baseline BP levels. Across all BP strata, mind–body activities consistently exhibited the lowest risk (≤4.5%), indicating the most favorable safety profile. Combined aerobic exercise and resistance training also showed a very low risk (≤1.6%) in patients with hypertension, particularly in stage 2. However, the risk associated with BFRE and high-intensity interval training increased with increasing baseline BP, reaching its highest level in patients with stage 2 hypertension. The risk associated with resistance training was the highest in individuals with elevated BP but decreased with increasing baseline BP (Supplementary S15).

#### 3.2.2. DBP

**Figure 3:**
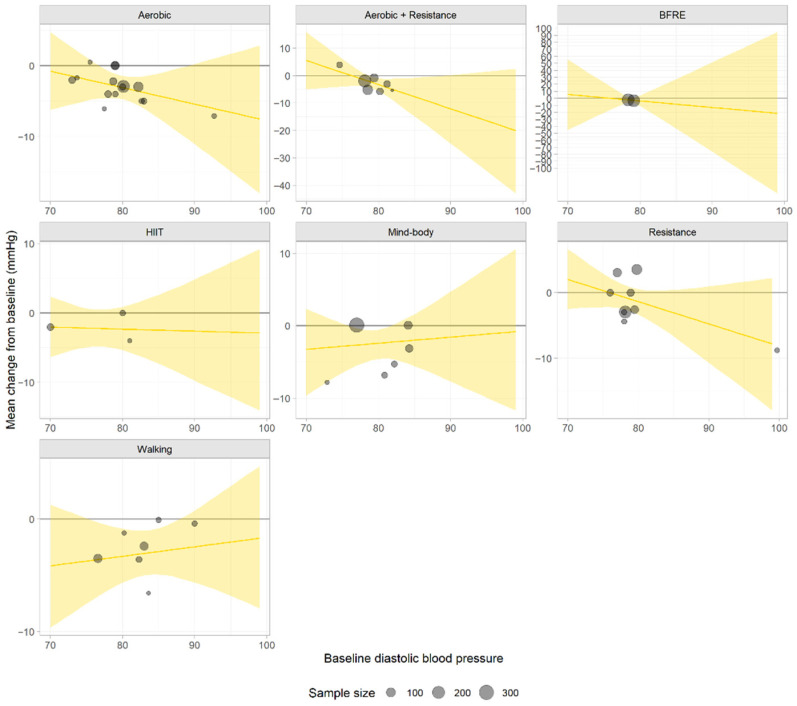
The meta-regression lines depict treatment-specific effects on diastolic blood pressure. The sample size of each study determines the area of its corresponding data point in the figure.

Among individuals with a baseline DBP of 75 mmHg (normal), none of the interventions resulted in a statistically significant reduction in DBP. In patients with stage 1 hypertension (baseline DBP, 85 mmHg), three interventions resulted in statistically significant reductions in DBP: combined aerobic and resistance training (CFB = −7.64; 95% CrI, −14.78 to −0.78), aerobic exercise (CFB = −4.27; 95% CrI, −7.41 to −1.11), and walking (CFB = −2.90; 95% CrI, −4.94 to −0.82). Among these interventions, only combined aerobic and resistance training exceeded the −5.0 mmHg threshold for a clinically meaningful change. In patients with stage 2 hypertension (baseline DBP, 90 mmHg), although the point estimates indicated the largest DBP reductions under combined aerobic exercise and resistance training (CFB = −12.04; 95% CrI, −24.73 to 0.15) and BFRE (CFB = −12.77; 95% CrI, −76.20 to 51.70), neither intervention resulted in a statistically significant change. The point estimate for aerobic exercise (CFB = −5.43; 95% CrI, −11.07 to 0.24) reached the clinically meaningful threshold but did not show statistical significance (Supplementary S8). The meta-regression lines are shown in Figure 3.

Based on available data, among individuals with a normal baseline DBP, walking showed a relatively high rank (rank value = 2.29), followed by mind–body activities (rank value = 2.96). In patients with stage 1 hypertension, combined aerobic and resistance training showed a relatively high rank (rank value = 2.13), followed by aerobic exercise (rank value = 3.29) and BFRE (rank value = 3.79). In patients with stage 2 hypertension, combined aerobic and resistance training maintained a relatively high rank (rank value = 2.09), suggesting consistent applicability in this population. Aerobic exercise (rank value = 3.45) and resistance training (rank value = 3.87) occupied intermediate and closely adjacent positions (Supplementary S12).

Analysis of treatment ranking probabilities indicated that among individuals with a normal baseline DBP, walking showed the highest probability of being the best treatment option (37%). In patients with stage 1 hypertension, BFRE exhibited the highest probability of being the best treatment option (50%), whereas combined aerobic and resistance training had a cumulative probability of 80% for ranking within the top two choices. These findings suggest that both of these interventions are the most likely optimal treatment choices for stage 1 hypertension, with the latter showing greater overall certainty in ranking. In patients with stage 2 hypertension, BFRE (best-option probability, 50%) and combined aerobic and resistance training (cumulative top-two probability, 82%) were the optimal treatment options (Supplementary S14).

Based on available data, among individuals with a normal baseline DBP, walking achieved a relatively high rank (rank value = 2.29). In patients with stage 1 hypertension, combined aerobic and resistance training showed a relatively high rank (rank value = 2.13). In patients with stage 2 hypertension, combined aerobic and resistance training maintained a relatively high rank (rank value = 2.09) (Supplementary S12). Predictive probability analysis suggested that walking was associated with a low predicted risk across all DBP strata (≤9.2%). In patients with stage 1 or 2 hypertension, both combined aerobic–resistance training and aerobic exercise exhibited low risk estimates (≤4.2%) (Supplementary S16).

#### 3.2.3. Dose–Response Relationship

Simple linear regression analysis indicated a significant inverse association between weekly physical activity dose and both SBP (β = −0.0042, *p* = 0.001) and DBP (β = −0.0023, *p* = 0.003). However, natural spline models showed a non-linear dose–response relationship. These models showed a statistically significant improvement in model fit over linear specification for both SBP (*p* < 0.01) and DBP (*p* < 0.01), indicating that a linear model was insufficient to capture the underlying relationship. To balance model flexibility with parsimony, natural spline models with two knots were used to characterize the dose–response relationship between physical activity and BP outcomes (Supplementary S17 and S21).

**Figure 4:**
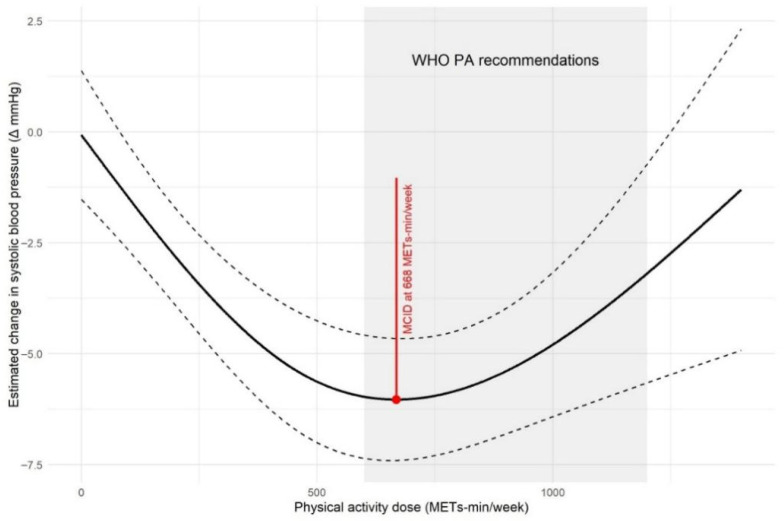
A Bayesian dose–response model was fitted to show the relationship between weekly activity dose (MET-min/week) and SBP. The solid line represents the posterior mean, flanked by dashed lines representing 95% CrI. Clinical reference points are marked: a red vertical line for the MCID (668 MET-min/week) and shading for the WHO-recommended range (600–1200 MET-min/week).

**Figure 5:**
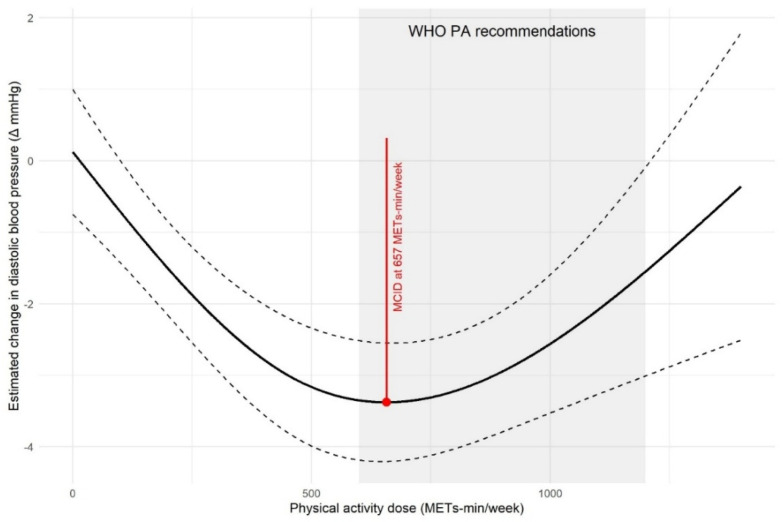
A Bayesian dose–response model was fitted to show the relationship between weekly activity dose (MET-min/week) and DBP. The solid line represents the posterior mean, flanked by dashed lines representing 95% CrI. Clinical reference points are marked: a red vertical line for the MCID (657 MET-min/week) and shading for the WHO-recommended range (600–1200 MET-min/week).

Figure 4 and Figure 5 show the association between physical activity dose and SBP or DBP reduction, alongside the estimated optimal doses. These doses fall within the lower end of the range (600–1200 MET-min/week) recommended by the World Health Organization. Although the WHO recommendation predominantly focuses on aerobic activity, our analysis estimates a total weekly dose that encompasses all modalities (aerobic exercise, resistance training, and mind–body activities). The doses are expressed in MET equivalents to enable comparison.

To determine the optimal dose of different exercise modalities for reducing SBP, dose–response curve fitting was performed for the exercise types meeting the criteria of sufficient sample size (*n* ≥ 5) and adequate dose variation (≥4 levels). An effect size was considered non-significant if its 95% confidence interval included zero. Consequently, the analysis was only able to identify optimal doses for specific modalities: For SBP reduction (Supplementary S20), the optimal doses were 860 MET-min/week for aerobic exercise (corresponding to a reduction of −7.4 mmHg) and 634 MET-min/week for walking (−8.9 mmHg). For DBP reduction (Supplementary S24), the optimal doses were 940 MET-min/week for aerobic exercise (−4.5 mmHg), 801 MET-min/week for combined aerobic and resistance training (−4.5 mmHg), and 596 MET-min/week for walking (−4.3 mmHg).

When the weekly exercise dose was categorized into low-dose (≤500 MET-min/week), medium-dose (501–1000 MET-min/week), and high-dose (>1000 MET-min/week) groups, a significant overall effect of dose category was observed for both SBP (F = 11.67, *p* < 0.001) and DBP (F = 8.1, *p* < 0.001). Post hoc comparisons using Tukey’s HSD test revealed that the medium-dose group showed the most pronounced benefits, with significant reductions in both SBP (−6.07 mmHg, *p* < 0.001) and DBP (−3.13 mmHg, *p* < 0.001) compared to those under usual care. The low-dose group showed a significant reduction in DBP (−2.72 mmHg, *p* = 0.014). However, the high-dose group showed no statistically significant difference from usual care for either SBP (*p* = 0.690) or DBP (*p* = 0.627) (Supplementary S18 and S22).

#### 3.2.4. Assessment of Methodological Quality and Publication Bias

The methodological quality of the 36 included studies was evaluated. Results revealed the following distribution of bias risk (Supplementary S25): A total of 10 (27.8%) studies showed a low risk of bias, indicating rigorous design and complete reporting of key methodological details, both of which support higher confidence in the study results. A total of 23 (63.9%) studies showed an unclear risk of bias, primarily owing to insufficient description of critical information concerning randomization, allocation concealment, or blinding. The remaining 3 (8.3%) studies showed a high risk of bias owing to notable methodological limitations. Publication bias was evaluated using funnel plots for visual inspection and Egger’s linear regression test for statistical verification. In the case of SBP, the funnel plot appeared largely symmetrical. This visual assessment was supported by the non-significant result of Egger’s test (*p* = 0.118). On the contrary, the funnel plot for DBP exhibited noticeable asymmetry, and Egger’s test indicated statistically significant evidence of small-study effects (*p* = 0.0095). This asymmetry suggests a potential risk of publication bias or other small-study effects for DBP outcomes (Supplementary S26).

## 4. Discussion

### 4.1. Interpretation with Regard to Previous Research

This study indicates that exercise significantly improves BP and other cardiovascular risk factors in type 2 diabetes, a finding that aligns with and reinforces the findings of previous studies [41]. This improvement in risk factors may represent a key mechanism linking physical activity to the reduced all-cause mortality and cardiovascular event rates reported by major trials. Notably, this beneficial effect varies considerably, depending on the specific form of exercise undertaken.

Resistance training, especially high-intensity resistance training [42], has been shown to significantly reduce BP. The underlying mechanisms may involve enhanced vasodilation through improved bioavailability [43] and increased muscle perfusion [44]. Aerobic training targets the complex pathophysiology of arterial stiffness, particularly in individuals with both diabetes and hypertension. Aerobic exercise attenuates excessive sympathetic tone while increasing the compliance of arteries. These changes promote equilibrium in autonomic function and contribute to a decrease in vascular stiffening. These outcomes are especially valuable for managing patients at later stages of hypertension [45]. Mind–body activities have favorable effects on a majority of biological cardiovascular risk factors, including BP [46]. The observed benefits likely result from reduced vascular resistance in both local and systemic circulation and from reduced cardiac output owing to the modulation of heart rate and stroke volume. In addition, combined aerobic and resistance training has shown synergistic BP-lowering potential in multiple studies, offering comprehensive benefits particularly for patients with hypertension with concomitant glucolipid metabolic disorders [47].

Dose–response analysis indicated that the optimal total physical activity dose required to achieve a significant BP reduction was approximately 668 MET-min/week for SBP (corresponding to a reduction of approximately 6.03 mmHg) and 657 MET-min/week for DBP (corresponding to a reduction of approximately 3.38 mmHg). These doses fall within the lower end of the WHO-recommended physical activity dose range, suggesting that clinically meaningful benefits can be achieved even when meeting the basic recommended physical activity level. It is noteworthy that our calculated optimal doses are lower than those reported by previous meta-analyses [23], likely owing to the exclusive inclusion of middle-aged and older adults in this meta-analysis—a population whose physical capacity, metabolic characteristics, and activity tolerance may differ from those of the general adult population, thereby requiring a relatively low effective dose.

Overall, the divergent physiological mechanisms indicate an underlying relationship between baseline vascular status and the preferential hemodynamic effects of each exercise type. This relationship accounts for the differential, stage-specific BP responses observed in this meta-analysis.

### 4.2. Clinical Implications

This systematic review evaluated the effects of different exercise interventions on SBP and DBP in middle-aged and older adults with type 2 diabetes. The findings indicate that the antihypertensive efficacy of exercise exhibits a clear dependence on both the type of BP elevation and the stage of hypertension, with notable differences in effectiveness and safety profiles.

For SBP management, the intervention effect was positively correlated with baseline levels. During the elevated BP stage (125 mmHg), walking was the only intervention that resulted in statistically significant and clinically meaningful reductions in BP. In patients with stage 1 (SBP, 135 mmHg) and stage 2 (SBP, 145 mmHg) hypertension, mind–body activities showed the most pronounced antihypertensive effect, highlighting their promise as a preferential SBP reduction strategy. Combined aerobic and resistance training also showed favorable effects in patients with stage 2 hypertension.

The effectiveness and certainty of evidence for DBP management presented a more complex scenario. No exercise intervention resulted in a statistically significant reduction in DBP during the normotensive stage (baseline, ~75 mmHg). For stage 1 hypertension (DBP, 85 mmHg), combined aerobic and resistance training was the only intervention that achieved the thresholds for both clinical meaningfulness and statistical significance. However, for stage 2 hypertension (90 mmHg), although the point estimates indicated the largest reductions under combined aerobic and resistance training and BFRE, neither intervention resulted in statistically significant reductions. These findings suggest considerable uncertainty regarding treatment efficacy in this population, warranting caution during clinical application.

Dose–response analysis offers one of the most actionable insights for clinical practice: maximum reductions occurred at approximately 668 MET-min/week for SBP and 657 MET-min/week for DBP. This suggests that a weekly dose of roughly 660 MET-min (corresponding to about 150–170 min of moderate-intensity exercise) is associated with optimal benefit. This dose falls at the lower end of the WHO recommendation (600–1200 MET-min/week), which is encouraging as it indicates that significant clinical benefit (approximately 5–6 mmHg reduction in SBP) is achievable by meeting the basic recommended activity level, potentially enhancing patient adherence. This study therefore propose a target of approximately 660 MET-min/week as an evidence-based starting point for personalized exercise prescription in adults with type 2 diabetes and elevated blood pressure.

### 4.3. Strengths and Limitations

This study indicates that the most appropriate type of physical activity for optimizing BP reduction in middle-aged and older adults with type 2 diabetes can be selected based on their specific SBP and DBP levels. The relative effect estimates derived from different baseline BP states provide direct evidence for this individualized selection. A Bayesian predictive model was used to estimate the probability of non-beneficial outcomes in future trials of exercise across diverse BP levels, thereby directly informing the clinical generalizability of the meta-analysis results. In addition, this study found a significant dose-dependent association between the total weekly physical activity dose and BP reduction, highlighting the importance of optimizing physical activity dosage in clinical practice. However, several limitations of this study should be acknowledged.

Some of the derived effect estimates are associated with considerable uncertainty. This uncertainty is likely attributed to substantial heterogeneity [23] in intervention protocols across the included primary studies and the limited sample sizes of certain trials, particularly those evaluating BFRE and HIIT. These factors constrain the precision of the estimates. Although coding HIIT without MET data as vigorous activity is justifiable, it introduces classification bias that warrants explicit acknowledgment. The preferential publication of positive results (publication bias) may influence the findings on DBP, potentially yielding less robust overall estimates of the treatment effect. To develop more precise individualized exercise regimens, future studies should focus on obtaining and analyzing individual patient-level data. This approach may enable more effective control of confounding factors, such as age, sex, disease duration, concomitant medications, and other potential covariates, on overall effect estimates, thereby enabling tailored exercise recommendations for patient subgroups with different characteristics [48]. We could not quantitatively assess effect modifiers (training status, medication, diabetes duration, and comorbidities) owing to a lack of individual-level data. Heterogeneity in BP measurement methods across the included studies (devices, operator technique, cuff specifications, and environmental conditions) may introduce a non-negligible measurement error. These methodological inconsistencies may affect the statistical power of between-intervention comparisons and the accuracy of the conclusion [26]. Finally, it must be emphasized that when estimating the optimal dose of different exercise modalities (e.g., aerobic, resistance, or mind–body interventions), the limited number of primary studies and relatively small sample sizes for certain activity categories introduce uncertainty into the dose–response relationship, thereby affecting the precision of the corresponding results, particularly point estimates and confidence intervals. Therefore, these “optimal dose” recommendations, derived from limited data, should be interpreted as preliminary references. Caution is warranted during their clinical interpretation and practical application, and further high-quality studies are warranted to validate and refine these estimates.

## 5. Conclusions

This systematic review indicates that the choice of a suitable exercise intervention for BP management in middle-aged and older adults with type 2 diabetes depends on the predominant type of elevated BP (systolic or diastolic) and its clinical stage. Mind–body activities were associated with significant reductions in SBP and appear to be a preferred intervention, especially for patients with stage 1 or 2 hypertension. For DBP management, combined aerobic and resistance training demonstrated efficacy in stage 1 hypertension, while its effects in stage 2 hypertension warrant cautious interpretation. Dose–response analysis suggests that a relatively moderate volume of physical activity—approximately 668 MET-min/week for SBP and 657 MET-min/week for DBP—may produce clinically meaningful reductions, aligning with the lower end of the WHO-recommended range. Importantly, an effective BP-lowering dose range varied across exercise modalities. Therefore, when designing individualized exercise regimens, it is important to consider the patient’s exercise preferences alongside their primary BP control target to guide tailored dose adjustments.

## Figures and Tables

**Table 1:** Hypertension classification and predicted values in meta-regression.

Classification	SBP	DBP	Predicted SBP	Predicted DBP
Normal	<120	<80	NA	NA
Elevated	120–129	<80	125	75
Hypertension stage 1	130–139	80–89	135	85
Hypertension stage 2	≥140	≥90	145	90

All values in this table are reported in mmHg. ‘NA’ denotes ‘not applicable’. A diagnosis of hypertension (stage 1 or 2) requires only one of the two listed systolic (SBP) or diastolic (DBP) pressure limits to be met.

## Data Availability

Data available on request from the authors. The data that support the findings of this study are available from the corresponding author, JZ, upon reasonable request.

## Supplementary Materials

The following supporting information can be downloaded at: https://www.mdpi.com/article/10.3390/life16050843/s1, S1 Search Strategy; S2 Characteristics of included studies; S3 Exploratory analysis; S4 Model fit comparison; S5 Network geometry at treatment-levels; S6 Inconsistency check; S7 Relative effect at treatment according to systolic blood pressure baseline; S8 Relative effect at treatment according to diastolic blood pressure baseline; S9 Meta-regression lines for each of the included interventions at treatment in systolic blood pressure; S10 Meta-regression lines for each of the included interventions at treatment in diastolic blood pressure; S11 Treatment ranking at treatment according to systolic blood pressure baseline; S12 Treatment ranking at treatment according to diastolic blood pressure baseline; S13 Rank probabilities at treatment according to systolic blood pressure baseline; S14 Rank probabilities at treatment according to diastolic blood pressure baseline; S15 Predictive probabilities that a new trial shows no or adverse effects applying different interventions on people with different baseline SBP levels; S16 Predictive probabilities that a new trial shows no or adverse effects applying different interventions on people with different baseline DBP levels; S17 Comparison of linear and nonlinear dose-response models; S18 Grouping analysis of systolic blood pressure dose; S19 Dose-response relationship between weekly physical activity and systolic blood pressure; S20 Dose response relationship of different exercises; S21 Comparison of linear and nonlinear dose-response models; S22 Grouping analysis of diastolic blood pressure dose; S23 Dose-response relationship between weekly physical activity and diastolic blood pressure; S24 Dose response relationship of different exercises; S25 Overall-level risk of bias of included studies; S26 Funnel plot for the assessment of publication bias; S27 Grade summary of all studies.

## Abbreviations

The following abbreviations are used in this manuscript:

T2DM	Type 2 diabetes mellitus
ADA	American Diabetes Association
BP	blood pressure
SE	standard error
SD	standard deviation
SD_change_	change scores
HIIT	high-intensity interval training
BFRE	blood flow restriction exercise
SBP, DBP	systolic and diastolic blood pressure
MmHG	millimetres of mercury
DIC	Deviance Information Criterion
UME	unrelated mean effects
MET	standard metabolic equivalent
PER	perceived exertion
