# Supplementary material for: Optimal Exercise Type and Dose for Blood Pressure Improvement in Middle-Aged and Older Adults with Type 2 Diabetes: A Systematic Review and Network Meta-Analysis
Source: Life (Basel). 2026 May 19;16(5):843. doi: 10.3390/life16050843 (PMC13208410; doi:10.3390/life16050843)
Supplement: Supplementary file 1 [file life-16-00843-s001.zip › life-4236838-supplementary.pdf]

Supplementary S1. Search Strategy....3

Supplementary S2. Characteristics of included studies....14

References....25

Supplementary S3. Exploratory analysis....29

Supplementary S4. Model fit comparison....31

Supplementary S5. Network geometry at treatment-levels....39

Supplementary S6. Inconsistency check....41

Supplementary S7. Relative effect at treatment according to systolic blood pressure baseline....47

Supplementary S8. Relative effect at treatment according to diastolic blood pressure baseline....50

Supplementary S9. Meta-regression lines for each of the included interventions at treatment in systolic blood pressure....53

Supplementary S10. Meta-regression lines for each of the included interventions at treatment in diastolic blood pressure....55

Supplementary S11. Treatment ranking at treatment according to systolic blood pressure baseline....58

Supplementary S12. Treatment ranking at treatment according to diastolic blood pressure baseline....60

Supplementary S13. Rank probabilities at treatment according to systolic blood pressure baseline....62

Supplementary S14. Rank probabilities at treatment according to diastolic blood pressure baseline....66

Supplementary S15. Predictive probabilities that a new trial shows no or adverse effects applying different interventions on people with different baseline SBP levels....69

Supplementary S16. Predictive probabilities that a new trial shows no or adverse effects applying different interventions on people with different baseline DBP levels....71

Supplementary S17. Comparison of linear and nonlinear dose-response models....73

Supplementary S18. Grouping analysis of systolic blood pressure dose....75

Supplementary S19. Dose-response relationship between weekly physical activity and systolic blood pressure....77

Supplementary S20. Dose response relationship of different exercises....78

Supplementary S21. Comparison of linear and nonlinear dose-response models....80

Supplementary S22. Grouping analysis of diastolic blood pressure dose....82

Supplementary S23. Dose-response relationship between weekly physical activity and diastolic blood pressure....84

Supplementary S24. Dose response relationship of different exercises....86

Supplementary S25. Overall-level risk of bias of included studies....88

Supplementary S26. Funnel plot for the assessment of publication bias. 91

Supplementary S27. Grade summary of all studies 93

1.Systolic blood pressure 93

2.Diastolic blood pressure 96

Supplementary S1. Search Strategy

**PubMed**	((((((((((((((((((((((((((((((("Diabetes Mellitus, Type 2"[Mesh]) OR (Diabetes Mellitus, Type 2[Title/Abstract]) OR (Diabetes Mellitus, Stable[Title/Abstract])) OR (Stable Diabetes Mellitus[Title/Abstract])) OR (Diabetes Mellitus, Noninsulin Dependent[Title/Abstract])) OR (Diabetes Mellitus, Adult-Onset[Title/Abstract])) OR (Adult-Onset Diabetes Mellitus[Title/Abstract])) OR (Diabetes Mellitus, Adult Onset[Title/Abstract])) OR (Diabetes Mellitus, Ketosis-Resistant [Title/Abstract])) OR (Diabetes Mellitus, Ketosis Resistant[Title/Abstract])) OR (Ketosis-Resistant Diabetes Mellitus[Title/Abstract])) OR (Diabetes Mellitus, Non Insulin Dependent[Title/Abstract])) OR (Diabetes Mellitus, Non-Insulin-Dependent[Title/Abstract])) OR (Non-Insulin-Dependent Diabetes Mellitus[Title/Abstract])) OR (Diabetes Mellitus, Type II[Title/Abstract])) OR (NIDDM [Title/Abstract])) OR (Diabetes Mellitus, Maturity-Onset[Title/Abstract])) OR (Diabetes Mellitus, Maturity Onset[Title/Abstract])) OR (Maturity-Onset Diabetes Mellitus[Title/Abstract])) OR (Maturity Onset Diabetes Mellitus[Title/Abstract])) OR (MODY[Title/Abstract])) OR (Diabetes Mellitus, Slow-Onset[Title/Abstract])) OR (Diabetes Mellitus, Slow Onset[Title/Abstract])) OR (Slow-Onset Diabetes Mellitus[Title/Abstract])) OR (Type 2 Diabetes Mellitus[Title/Abstract])) OR (Noninsulin-Dependent Diabetes Mellitus[Title/Abstract])) OR

	(Noninsulin Dependent Diabetes Mellitus[Title/Abstract])) OR (Maturity-Onset Diabetes[Title/Abstract])) OR (Diabetes, Maturity-Onset[Title/Abstract])) OR (Maturity Onset Diabetes[Title/Abstract])) OR (Type 2 Diabetes[Title/Abstract])) OR (Diabetes, Type 2[Title/Abstract])) OR (Diabetes Mellitus, Noninsulin-Dependent[Title/Abstract])
	(((Exercise[Title/Abstract]) OR (Exercise Therapy[Title/Abstract])) OR (sports[Title/Abstract])) OR (Physical Activity[Title/Abstract])) OR (Physical Activities[Title/Abstract])) OR (Physical Training[Title/Abstract])) OR (muscle strength[Title/Abstract])) OR (muscle-strengthening[Title/Abstract])) OR (muscle training[Title/Abstract])) OR (strength training[Title/Abstract])) OR (weight lifting[Title/Abstract])) OR (weight-lifting[Title/Abstract])) OR (weight bearing[Title/Abstract])) OR (weight-bearing[Title/Abstract])) OR (weight training[Title/Abstract])) OR (resistance exercise[Title/Abstract])) OR (resistance training[Title/Abstract])) OR (resistive training [Title/Abstract])) OR (aerobic training[Title/Abstract])) OR (endurance training[Title/Abstract])) OR (stability training[Title/Abstract])) OR (Flexibility training[Title/Abstract])) OR (Isometric Training[Title/Abstract])) OR (Isokinetic Training[Title/Abstract])) OR (Running[Title/Abstract])) OR (Walking[Title/Abstract])) OR (jogging[Title/Abstract])) OR (sprinting[Title/Abstract])) OR (cycling[Title/Abstract])) OR (rowing[Title/Abstract])) OR (swimming[Title/Abstrac

	t])) OR (bicycling[Title/Abstract])) OR (treadmill[Title/Abstract])) OR (dancing[Title/Abstract])) OR (Tai Chi[Title/Abstract])) OR (qigong[Title/Abstract])) OR (qi gong[Title/Abstract])) OR (Yoga[Title/Abstract])) OR (Tai-Ji[Title/Abstract])) OR (Taiji[Title/Abstract])) OR (meditative movement[Title/Abstract])) OR (Meditation[Title/Abstract])) OR (eight section brocades[Title/Abstract])) OR (Mindfulness[Title/Abstract])) OR (Liuzijue[Title/Abstract])) OR (Yijinjing[Title/Abstract])) OR (traditional Chinese exercise[Title/Abstract])) OR (Chi Kung[Title/Abstract])) OR (pilates[Title/Abstract])) OR (dance[Title/Abstract])) OR (five animals[Title/Abstract])) OR (Wuqinxi[Title/Abstract])) OR (Baduanjin[Title/Abstract])) OR (Mind Body Therapies[Title/Abstract])) OR (Mind Body Therapies[Title/Abstract])) OR (Mind Body Medicine[Title/Abstract])) OR (mind body medicine[Title/Abstract])) OR (neuromuscular training[Title/Abstract])
	(((((((((((Aged[Title/Abstract]) OR (Frail Elderly[Title/Abstract])) OR (aged[Title/Abstract])) OR (elder[Title/Abstract])) OR (senior[Title/Abstract])) OR (older[Title/Abstract])) OR (old people[Title/Abstract])) OR (old adult[Title/Abstract])) OR (retired[Title/Abstract])) OR (aging[Title/Abstract]) OR (middle elderly[Title/Abstract]) OR (middle age[Title/Abstract]) OR (middle aged[Title/Abstract])

	((((randomized controlled trial[Publication Type]) OR (randomized controlled trial[Title/Abstract])) OR (randomized controlled trials[Title/Abstract])) OR (randomised controlled trial[Title/Abstract])) OR (randomised controlled trials[Title/Abstract])
	("Blood Pressure"[Mesh]) OR ((((((((Pressure, Blood[Title/Abstract]) OR (Pulse Pressure[Title/Abstract])) OR (Pressure, Pulse[Title/Abstract])) OR (Diastolic Pressure[Title/Abstract])) OR (Pressure, Diastolic[Title/Abstract])) OR (Systolic Pressure[Title/Abstract])) OR (Pressures, Systolic[Title/Abstract])) OR (Pressure, Systolic[Title/Abstract]))

**Web of Science**	Diabetes Mellitus, Type 2 OR Diabetes Mellitus, Stable OR Stable Diabetes Mellitus OR Diabetes Mellitus, Noninsulin Dependent OR Diabetes Mellitus, Adult-Onset OR Adult-Onset Diabetes Mellitus OR Diabetes Mellitus, Adult Onset OR Diabetes Mellitus, Ketosis-Resistant OR Diabetes Mellitus, Ketosis Resistant OR Ketosis-Resistant Diabetes Mellitus OR Diabetes Mellitus, Non Insulin Dependent OR Diabetes Mellitus, Non-Insulin-Dependent OR Non-Insulin-Dependent Diabetes Mellitus OR Diabetes Mellitus, Type II OR NIDDM OR Diabetes Mellitus, Maturity-Onset OR Diabetes Mellitus, Maturi

	ty Onset OR Maturity-Onset Diabetes Mellitus OR Maturity Onset Diabetes Mellitus OR MODY OR Diabetes Mellitus, Slow-Onset OR Diabetes Mellitus, Slow Onset OR Slow-Onset Diabetes Mellitus OR Type 2 Diabetes Mellitus OR Noninsulin-Dependent Diabetes Mellitus OR Noninsulin Dependent Diabetes Mellitus OR Maturity-Onset Diabetes OR Diabetes, Maturity-Onset OR Maturity Onset Diabetes OR Type 2 Diabetes OR Diabetes, Type 2 OR Diabetes Mellitus, Noninsulin-Dependent
	Exercise OR Exercise Therapy OR sports OR Physical Activity OR Physical Activities OR Physical Training OR muscle strength OR muscle-strengthening OR muscle training OR strength training OR weight lifting OR weight-lifting OR weight bearing OR weight-bearing OR weight training OR resistance exercise OR resistance training OR resistive training OR aerobic training OR endurance training OR stability training OR Flexibility training OR Isometric Training OR Isokinetic Training OR Running OR Walking OR jogging OR sprinting OR cycling OR rowing OR swimming OR bicycling OR treadmill OR dancing OR Tai Chi OR qigong OR qi gong OR Yoga OR Tai-Ji OR Taiji OR meditative movement OR Meditation OR eight section brocades OR Mindfulness OR Liuzijue OR Yijinjing OR traditional Chinese exercise OR Chi Kung OR pilates OR dance OR five animals OR Wuqinxi O

	R Baduanjin OR Mind Body Therapies OR Mind Body Therapies OR Mind Body Medicine OR mind body medicine OR neuromuscular training
	Aged OR Frail Elderly OR aged OR elder OR senior OR older OR old pe ople OR old adult OR retired OR aging OR middle elderly OR middle age OR middle aged
	Blood Pressure OR Pressure, Blood OR Pulse Pressure OR Pressure, Pulse OR Diastolic Pressure OR Pressure, Diastolic OR Systolic Pressure OR Pressures, Systolic OR Pressure, Systolic
	randomized controlled trial OR randomized controlled trials OR randomised controlled trial OR randomised controlled trials

**Emabase**	'non insulin dependent diabetes mellitus'/exp OR 'adult onset diabetes':ab,ti OR 'adult onset diabetes mellitus':ab,ti OR 'diabetes mellitus type 2':ab,ti O R 'diabetes mellitus type ii':ab,ti OR 'diabetes mellitus, maturity onset':ab,ti OR 'diabetes mellitus, non insulin dependent':ab,ti OR 'diabetes mellitus, non

	-insulin-dependent':ab,ti OR 'diabetes mellitus, type 2':ab,ti OR 'diabetes mellitus, type II':ab,ti OR 'diabetes type 2':ab,ti OR 'diabetes type II':ab,ti OR 'diabetes, adult onset':ab,ti OR 'dm 2':ab,ti OR 'insulin independent diabetes':ab,ti OR 'insulin independent diabetes mellitus':ab,ti OR 'ketosis resistant diabetes mellitus':ab,ti OR 'maturity onset diabetes':ab,ti OR 'maturity onset diabetes mellitus':ab,ti OR 'niddm':ab,ti OR 'NIDDM (non insulin dependent diabetes mellitus)':ab,ti OR 'non insulin dependent (type 2) diabetes mellitus':ab,ti OR 'non insulin dependent diabetes':ab,ti OR 'non-insulin-dependent diabetes mellitus':ab,ti OR 'noninsulin dependent (type 2) diabetes mellitus':ab,ti OR 'noninsulin dependent diabetes':ab,ti OR 'noninsulin dependent diabetes mellitus':ab,ti OR 'T2DM':ab,ti OR 'TIIDM':ab,ti OR 'type 2 (insulin independent) diabetes':ab,ti OR 'type 2 diabetes':ab,ti OR 'type 2 diabetes mellitus':ab,ti OR 'type II diabetes':ab,ti OR 'type II diabetes mellitus':ab,ti OR 'non insulin dependent diabetes mellitus':ab,ti
	'Exercise':ab,ti OR 'Exercise Therapy':ab,ti OR 'sports':ab,ti OR 'Physical Activity':ab,ti OR 'Physical Activities':ab,ti OR 'Physical Training':ab,ti OR 'muscle strength':ab,ti OR 'muscle-strengthening':ab,ti OR 'muscle training':ab,ti OR 'strength training':ab,ti OR 'weight lifting':ab,ti OR 'weight-lifting':ab,ti OR 'weight bearing':ab,ti OR 'weight-bearing':ab,ti OR 'weight training':ab,ti OR '

	resistance exercise':ab,ti OR 'resistance training':ab,ti OR 'resistive training':ab,ti OR 'aerobic training':ab,ti OR 'endurance training':ab,ti OR 'stability training':ab,ti OR 'Flexibility training':ab,ti OR 'Isometric Training':ab,ti OR 'Isokinetic Training':ab,ti OR 'Running':ab,ti OR 'Walking':ab,ti OR 'jogging':ab,ti OR 'sprinting':ab,ti OR 'cycling':ab,ti OR 'rowing':ab,ti OR 'swimming':ab,ti OR 'bicycling':ab,ti OR 'treadmill':ab,ti OR 'dancing':ab,ti OR 'Tai Chi':ab,ti OR 'qigong':ab,ti OR 'qi gong':ab,ti OR 'Yoga':ab,ti OR 'Tai-Ji':ab,ti OR 'Taiji':ab,ti OR 'meditative movement':ab,ti OR 'Meditation':ab,ti OR 'eight section brocades':ab,ti OR 'Mindfulness':ab,ti OR 'Liuzijue':ab,ti OR 'Yijinjing':ab,ti OR 'traditional Chinese exercise':ab,ti OR 'Chi Kung':ab,ti OR 'pilates':ab,ti OR 'dance':ab,ti OR 'five animals':ab,ti OR 'Wuqinxi':ab,ti OR 'Baduanjin':ab,ti OR 'Mind Body Therapies':ab,ti OR 'Mind Body Therapies':ab,ti OR 'Mind Body Medicine':ab,ti OR 'mind body medicine':ab,ti OR 'neuromuscular training'
	'Aged':ab,ti OR 'Frail Elderly':ab,ti OR 'aged':ab,ti OR 'elder':ab,ti OR 'senior':ab,ti OR 'older':ab,ti OR 'old people':ab,ti OR 'old adult':ab,ti OR 'retired':ab,ti OR 'aging':ab,ti OR 'middle elderly' OR 'middle age' OR 'middle aged'
	'randomized controlled trial':ab,ti OR 'randomized controlled trials':ab,ti OR 'r

	andomised controlled trial':ab,ti OR 'randomised controlled trials':ab,ti
	'blood pressure'/exp OR 'blood tension':ab,ti OR 'intravascular pressure':ab,ti OR 'normotension':ab,ti OR 'pressure, blood':ab,ti OR 'vascular pressure':ab,ti OR 'blood pressure':ab,ti OR 'Diastolic Pressure':ab,ti OR 'Pressure, Diastolic':ab,ti OR 'Systolic Pressure':ab,ti OR 'Pressures, Systolic':ab,ti OR 'Pressure, Systolic'

**Cochrane Library**	(Maturity-Onset Diabetes Mellitus):ti,ab,kw OR (Diabetes Mellitus, Slow-Onset):ti,ab,kw OR (Type 2 Diabetes):ti,ab,kw OR (Adult-Onset Diabetes Mellitus):ti,ab,kw OR (Ketosis-Resistant Diabetes Mellitus):ti,ab,kw OR (Diabetes Mellitus, Type II):ti,ab,kw OR (Slow-Onset Diabetes Mellitus):ti,ab,kw OR (Type 2 Diabetes Mellitus):ti,ab,kw OR (Diabetes Mellitus, Noninsulin-Dependent):ti,ab,kw OR (Maturity Onset Diabetes):ti,ab,kw OR (Diabetes Mellitus, Non Insulin Dependent):ti,ab,kw OR (Diabetes Mellitus, Slow Onset):ti,ab,kw OR (Diabetes Mellitus, Noninsulin Dependent):ti,ab,kw OR (Non-Insulin-Dependent Diabetes Mellitus):ti,ab,kw OR (Diabetes Mellitus, Ketosis-Resistant):ti,ab,kw OR (Maturity-Onset Diabetes):ti,ab,kw OR (Diabetes Mellitus, Stable):ti,ab,kw OR (Diabetes Mellitus, Ketosis Resistant):ti,ab,kw OR (Diabetes Mellitus, Maturity Onset):ti,ab,kw OR (Noninsulin Dependent Diabetes Mellit

	us):ti,ab,kw OR (Diabetes Mellitus, Adult Onset):ti,ab,kw OR (NIDDM):ti,ab,kw OR (Diabetes Mellitus, Adult-Onset):ti,ab,kw OR (Diabetes Mellitus, Maturity-Onset):ti,ab,kw OR (Maturity Onset Diabetes Mellitus):ti,ab,kw OR (Diabetes, Type 2):ti,ab,kw OR (Diabetes, Maturity-Onset):ti,ab,kw OR (MODY):ti,ab,kw OR (Noninsulin-Dependent Diabetes Mellitus):ti,ab,kw OR (Diabetes Mellitus, Non-Insulin-Dependent):ti,ab,kw OR (Stable Diabetes Mellitus):ti,ab,kw
	(Exercise OR Exercise Therapy OR sports OR Physical Activity OR Physical Activities OR Physical Training OR muscle strength OR muscle-strengthening OR muscle training OR strength training OR weight lifting OR weight-lifting OR weight bearing OR weight-bearing OR weight training OR resistance exercise OR resistance training OR resistive training OR aerobic training OR endurance training OR stability training OR Flexibility training OR Isometric Training OR Isokinetic Training OR Running OR Walking OR jogging OR sprinting OR cycling OR rowing OR swimming OR bicycling OR treadmill OR dancing OR Tai Chi OR qigong OR qi gong OR Yoga OR Tai-Ji OR Taiji OR meditative movement OR Meditation OR eight section brocades OR Mindfulness OR Liuzijue OR Yijinjing OR traditional Chinese exercise OR Chi Kung OR pilates OR dance OR five animals OR Wuqinxi

	OR Baduanjin OR Mind Body Therapies OR Mind Body Therapies OR Mind Body Medicine OR mind body medicine OR neuromuscular training):ti,ab,kw
	(Aged OR Frail Elderly OR aged OR elder OR senior OR older OR old people OR old adult OR retired OR aging OR middle elderly OR middle age OR middle aged):ti,ab,kw
	(Blood Pressure OR Pressure, Blood OR Pulse Pressure OR Pressure, Pulse OR Diastolic Pressure OR Pressure, Diastolic OR Systolic Pressure OR Pressures, Systolic OR Pressure, Systolic):ti,ab,kw
	(randomized controlled trial OR randomized controlled trials OR randomised controlled trial OR randomised controlled trials):ti,ab,kw

Supplementary S2. Characteristics of included studies

StudyID	Experimental/control group samples	Average age of experimental group /control group	Males/n	Blood pressure Baseline/mmHg	Duration/ Frequency	Experimental group	Control group
Theng Choon Ooi 2021[1]	28/31	61.79±5.11/61.58 ±5.92	EG:12/28<br>CG:15/31	EG:<br>Sys:134.14<br>Dia:78.9<br>CG:<br>Sys:146.56<br>Dia:78.41	16 weeks<br>3 times/weeks	Resistance<br>Intensity:70%1RM	Usual care
Sadanori Okada 2010[2]	21/17	61.9±8.6/64.5±5.9	EG:10/21<br>CG:11/17	EG:<br>Sys:129<br>Dia:74.6<br>CG:<br>Sys:126.6<br>Dia:73.8	12 weeks<br>3-5 times/week<br>60 min/time	Aerobic + Resistance<br>Aerobic：60% of HR reserve<br>Resistance：50-69%1RM	Usual care
Taisa Belli 20	9/10	53.4±2.3/55.9±2.2	EG:0/9	EG:	12 weeks	Walk	Usual care

11[3]			CG:0/10	Sys:130<br>Dia:83.6<br>CG:<br>Sys:138<br>Dia:84.4	3 times/week<br>60 min/time	4.7-5.4 km/h	
Nesrin Doğan Dede 2014[4]	30/30	52.5±7.5/55.5±8.4	EG:15/30<br>CG:14/30	EG:<br>Sys:127.6<br>Dia:78.7<br>CG:<br>Sys:127.2<br>Dia:77.7	12 weeks<br>3 times/week<br>45 min/time	Aerobic<br>60-75% HR max	Usual care
KENNETH M. MADDEN 2009[5]	18/18	71.7±1.1/71.1±0.9	EG:NA<br>CG:NA	EG:<br>Sys:143<br>Dia:83<br>CG:<br>Sys:150<br>Dia:86	12 weeks<br>3 times/week<br>40 min/time	Aerobic<br>60-75% HRR	Usual care
Huimin Yan 2014[6]	31/10	53±2/55±3	EG:31/31<br>CG:10/10	EG:<br>Sys:131<br>Dia:80<br>CG:<br>Sys:145<br>Dia:80	12 weeks<br>3-5 times/week<br>45 min/time	Aerobic<br>50-70% VO_2peak	Usual care
Kimberley L. Way 2020[7]	12/12/11	54.8 ± 2.4/56.9 ± 2.1/51.9±1.4	EG:7/12<br>EG:6/12<br>CG: 7/11	EG:<br>Sys:138<br>Dia:80	12 weeks<br>3 times/week<br>HIIT: 19 min/	HIIT:<br>90%VO_2peak	Usual care

				EG:<br>Sys:125<br>Dia:79<br>CG:<br>Sys:127<br>Dia:83	time<br>Aerobic：55 min/time	Aerobic: 60% VO₂peak	
Vijay Viswanathan 2020[8]	150/150	50.8±8.3/52.8±7.0	EG:93/150<br>CG:103/150	EG:<br>Sys:124.4<br>Dia:77<br>CG:<br>Sys:121.3<br>Dia:76.7	12 weeks<br>5 times/week<br>50 min/time	NA	Usual care
Arsalan Tariq 2025[9]	55/60	58.3±3.9/60.8±6.1	EG:55/55<br>CG:60/60	EG:<br>Sys:135.6<br>Dia:82.2<br>CG:<br>Sys:143.2<br>Dia:81.5	12 weeks<br>4 times/week<br>50 min/time	Aerobic cycling<br>55–60 rpm;<br>30-60W	Usual care
Javier Parra-Sánchez 2015[10]	47/41	73.2±4.8/72.6±5.1	NA	EG:<br>Sys:136.9<br>Dia:76.6<br>CG:<br>Sys:138.1<br>Dia:76.6	12 weeks<br>2 times/week<br>40 min/time	Walk<br>50%-65% HRR	Usual care
Cindy LW Ng 2010[11]	25/24	57±7/59±7	NA	EG:<br>Sys:123	8 weeks<br>2-3 times/wee	Resistance：<br>65% 1RM	Aerobic：<br>65%HRmax

				Dia:76<br>CG:<br>Sys:133<br>Dia:78	k<br>50 min/time		
Kelly A McDermott 2014[12]	20/18	47±9.7/47.2±9.1	EG:9/20<br>CG:7/21	EG:<br>Sys:122.9<br>Dia:82.2<br>CG:<br>Sys:127.6<br>Dia:82.3	8 weeks<br>3-6 times/week<br>75 min/time	Yoga:<br>75min	Walking:<br>75min
Xiaojun Ma 2024[13]	34/31/33	57.5 ± 2.4/60.8 ± 2.2/57.1±3	EG1:12/34<br>EG2:13/31<br>CG:14/33	EG1:<br>Sys:126.65<br>Dia:79.12<br>EG2:<br>Sys:128.68<br>Dia:78.13<br>EG:<br>Sys:127.73<br>Dia:79.97	24 weeks<br>3 times/week<br>50 min/time	BFRE: 20-30% 1-RM<br>Resistance: 60-70% 1-RM	Usual care
KRISTIAN KARSTOFT2013[14]	12/12/8	57.5 ± 2.4/60.8 ± 2.2/57.1±3	EG1：7/12<br>EG2：8/12<br>CG： 5/8	EG1:<br>Sys:138<br>Dia:85<br>EG2:<br>Sys:155<br>Dia:90	16 weeks<br>5 times/week<br>60 min/time	Interval-Walking Group：65.7 %HRmax<br>Continuous-Walking Gr	Usual care

				CG:<br>Sys:142<br>Dia:86.6		oup: 66.4% HRmax	
Rodrigo S. Delevatti 2016[15]	11/10	54.2±8.3/59.2±6.9	NA	EG:<br>Sys:130<br>Dia:85<br>CG:<br>Sys:142<br>Dia:86.6	12 weeks<br>3 times/week<br>45 min/time	Aquatic Training: 85-100%HRAT	Dry-Land Training: 85-100%HRAT
Sophie Cassidy 2019[16]	11/11	60±3/59±3	EG:9/11<br>CG:8/11	EG:<br>Sys:118<br>Dia:81<br>CG:<br>Sys:119<br>Dia:78	12 weeks<br>3 times/week<br>45 min/time	HIIT:<br>Borg RPE:16-17<br>Rest:2min	Usual care
F. Bellavere 2018[17]	19/11	57.1±1.6/60±6.8	NA	EG:<br>Sys:135.67<br>Dia:82.67<br>CG:<br>Sys:127<br>Dia:78	16 weeks<br>3 times/week<br>60 min/time	Aerobic: 60-65% HRR	Resistance: 70-80%1-RM
Giosuè Annibalini 2017[18]	8/8	57±9.1/60±6.8	EG:8/8<br>CG:8/8	EG:<br>Sys:126.6<br>Dia:81.9<br>CG:	16 weeks<br>3 times/week<br>60-90 min/time	Aerobic+Resistance<br>Aerobic:40-65%HRR;30-60min	Usual care

				Sys:130.6<br>Dia:80.6		Resistance:40-60%1-RM	
lka Yolane Teixeira Passos de Andrade 2022[19]	11/11	65.5±5.5/67.5±6.3	EG:0/11<br>CG:0/11	EG:<br>Sys:134.9<br>Dia:72.9<br>CG:<br>Sys:126.5<br>Dia:74.6	12 weeks<br>3 times/week<br>60 min/time	Yoga：Moderate intensity (RPE:11–12 on Borg 6–20 scale)	Usual care
TANYA D. AGURS-COLLINS 1997[20]	32/32	62.4±5.9/61.0±5.7	EG:11/32<br>CG:4/32	EG:<br>Sys:144<br>Dia:79<br>CG:<br>Sys:139<br>Dia:77	24 weeks<br>1 times/week<br>30 min/time	Aerobic：Moderate intensity	Usual care
Børge Moe 2011[21]	13/13	56.23 ± 8.29/57.77 ±7.82	EG:13/13<br>CG:13/13	EG:<br>Sys:145.7<br>Dia:92.7<br>CG:<br>Sys:151<br>Dia:99.7	12 weeks<br>3 times/week<br>45 min/time	Aerobic：75-85%VO_2 peak	Resistance：60% 1RM
Chueh-Lung Hwang 2019[22]	18/16/16	65±2/<br>62±2/<br>61±2	EG:9/18<br>EG:10/16<br>CG:8/16	EG:<br>Sys:124<br>Dia:70<br>EG:<br>Sys:127	8 weeks<br>4 times/week<br>40 min/time	HIIT：80%HRR<br>Aerobic：55% HRR	Usual care

				Dia:73<br>CG:<br>Sys:126<br>Dia:73			
Fangli Tang 2024[23]	37/37	65.919±3.974/<br>67.892±4.713	EG:16/37<br>CG:18/37	EG:<br>Sys:145.7<br>Dia:92.7<br>CG:<br>Sys:151<br>Dia:99.7	24 weeks<br>3 times/week<br>40 min/time	Resistance:<br>45-55%1RM	Usual care
Nikolaos P. E. Kadoglou 2012[24]	23/24	61.5±5.4/<br>64.6±4.3	EG:7/23<br>CG:5/24	EG:<br>Sys:121<br>Dia:71<br>CG:<br>Sys:144<br>Dia:83	12 weeks<br>3 times/week<br>45-60 min/time	Resistance:<br>60-80%1RM	Usual care
Shujuan Hu2022[25]	19/19	62.68±7.33/<br>61.58±6.62	NA	EG:<br>Sys:143.05<br>Dia:80.84<br>CG:<br>Sys:138.16<br>Dia:84.32	12 weeks<br>4 times/week<br>50 min/time	Taichi(24-type)	Usual care
Uttio Gupta2020[26]	38/40	51.1±8.6/<br>50.2±8.6	EG:21/38<br>CG:24/40	EG:<br>Sys:130.9<br>Dia:84.1<br>CG:	16weeks	Yoga:<br>2-3times/week<br>45 min/time	Walk:<br>5 times/week<br>30 min/time

				Sys:130.5<br>Dia:83			5-6km
Aswathy Sree devi 2017[27]	32/30	51.97±7.4/<br>51.92±6.57	EG:0/32<br>CG:0/30	EG:<br>Sys:133.81<br>Dia:84.25<br>CG:<br>Sys:126.06<br>Dia:81.84	12 weeks<br>2 times/week<br>60 min/time	Yoga	Usual care
Antti Loimaa la 2007[28]	24/24	52.8±5.2/<br>52.8±6.0	EG:24/24<br>CG:24/24	EG:<br>Sys:144<br>Dia:NA<br>CG:<br>Sys:146<br>Dia:NA	48 weeks<br>4 times/week	Aerobic+Resistance：<br>Aerobic:65–75%VO_2max	Usual care
Matthew D. HORDERN 2008[29]	68/64	57±11/<br>65±8	EG:36/68<br>CG:34/64	EG:<br>Sys:137.1<br>Dia:79.8<br>CG:<br>Sys:129.2<br>Dia:76.9	4 weeks<br>3 times/week<br>50 min/time	Resistance:<br>Borg RPE 12–13 on 20-point scale	Usual care
Romeu Mendes 2017[30]	39/85	62.05±7.62/<br>63.88±7.62	EG:19/39<br>CG:41/85	EG:<br>Sys:134.02<br>Dia:78.49<br>CG:<br>Sys:136.42<br>Dia:79.33	36 weeks<br>3 times/week<br>70 min/time	Aerobic+Resistance<br>Moderate-to-vigorous intensity (Borg RPE 12–	Usual care

						17)	
Yasuo Terauchi 2022[31]	97/108	54.6±9.2/<br>55.7±10.2	EG:68/97<br>CG:69/108	EG:<br>Sys:126.2<br>Dia:78.1<br>CG:<br>Sys:126<br>Dia:778	12 weeks<br>3 times/week<br>60 min/time	Aerobic+Resistance<br>Aerobic: moderate intensity<br>Resistance: moderate intensity	Usual care
Chathuranga Ranasinghe 2021[32]	28/28/30	52.0±9.8/<br>49.0±9.2/<br>49.3±7.0	EG:11/28<br>EG:13/28<br>CG:16/30	EG:<br>Sys:129<br>Dia:79<br>EG:<br>Sys:126<br>Dia:77<br>CG:<br>Sys:121<br>Dia:80	12 weeks<br>2 times/week<br>75 min/time	Aerobic: 60-75% HRmax<br>Resistance: 50% 1RM	Usual care
Alfonso Bellia 2017[33]	11/11	58.8±7.9/56.3±6.4	EG:9/11<br>CG:7/11	EG:<br>Sys:117.75<br>Dia:75.5<br>CG:<br>Sys:121.5<br>Dia:80.25	12 weeks<br>3 times/week<br>60 min/time	Aerobic: 75-80% HRmax	Walk: 10000steps/day
João P. Magalhães 2019[34]	16/13/22	60.4±6.8/<br>58.9±7.5/	NA	EG:<br>Sys:135.6	52 weeks<br>3 times/week	Aerobic+Resistance1: m	Usual care

		60.8±7.5		Dia:81.2<br>EG:<br>Sys:137.8<br>Dia:80.2<br>CG:<br>Sys:136.5<br>Dia:80.1	40 min/time	oderate intensity<br>Aerobic+Resistance2：moderate-high intensity	
Mohammed Amin 2023[35]	43/36	56.2±8.3/<br>56.5±8.5	NA	EG:<br>Sys:141.5<br>Dia:79.4<br>CG:<br>Sys:135.6<br>Dia:78.3	12 weeks<br>3 times/week<br>45 min/time	Aerobic+Resistance：moderate intensity	Usual care
Xiaojun Ma 2024[36]	30/32/<br>31	57.73±5.85/<br>57.56±4.85/<br>56.29±5.92	EG:16/30<br>EG:16/32<br>CG:11/31	EG:<br>Sys:130.67<br>Dia:80.13<br>EG:<br>Sys:131.31<br>Dia:78.34<br>CG:<br>Sys:132.48<br>Dia:79.06	24 weeks<br>3 times/week	Aerobic：40-59% HRR; 60min<br>BFRE: 20-40% 1-RM;40min	Usual care

EG: Experimental group; CG: Control group; BFRE: Blood flow restriction exercise; HIIT: High-intensity interval training; Sys: systolic pressure; Dia: diastolic pressure; rpm: Revolutions Per Minute; HRR: Heart Rate Reserve; 1RM: One Repetition Maximum; HRAT: Heart Rate at the Anaerobic Threshold; VO2max: Maximal oxygen uptake; VO2peak: Peak oxygen uptake; HRmax: Maximum Heart Rate; Borg RPE: Borg Rating of Perceived Exertion (RPE) Scale

References

[1]Ooi, T.C., et al., A 16-Week Home-Based Progressive Resistance Tube Training Among Older Adults With Type-2 Diabetes Mellitus: Effect on Glycemic Control. Gerontology and Geriatric Medicine, 2021. 7: p. 233372142110387

[2]Okada, S., et al., Effect of Exercise Intervention on Endothelial Function and Incidence of Cardiovascular Disease in Patients with Type 2 Diabetes. Journal of Atherosclerosis and Thrombosis, 2010. 17(8): p. 828-833.

[3]Belli, T., et al., Effects of 12-week overground walking training at ventilatory threshold velocity in type 2 diabetic women. Diabetes Research and Clinical Practice, 2011. 93(3): p. 337-343.

[4]Dede, N.D., et al., Influence of Exercise on Leptin, Adiponectin and Quality of Life in Type 2 Diabetics. Turkish Journal of Endocrinology and Metabolism, 2015. 19(1): p. 7-13.

[5]Madden, K.M., et al., Short-Term Aerobic Exercise Reduces Arterial Stiffness in Older Adults With Type 2 Diabetes, Hypertension, and Hypercholesterolemia. Diabetes Care, 2009. 32(8): p. 1531-1535.

[6]Yan, H., et al., Effect of Aerobic Training on Glucose Control and Blood Pressure in T2DDM East African Males. ISRN Endocrinology, 2014. 2014: p. 1-6.

[7]Way, K.L., et al., The effect of low-volume high-intensity interval training on cardiovascular health outcomes in type 2 diabetes: A randomised controlled trial. International Journal of Cardiology, 2020. 320: p. 148-154.

[8]Viswanathan, V., et al., Effect of yoga intervention on biochemical, oxidative stress markers, inflammatory markers and sleep quality among subjects with type 2 diabetes in South India: Results from the SATYAM project. Diabetes Research and Clinical Practice, 2021. 172: p. 108644.

[9]Tariq, A., et al., Pedalling towards better health: a randomised controlled trial of aerobic cycling to improve glycaemic control and quality of life in middle-aged men with type 2 diabetes. BMJ Nutrition, Prevention & Health, 2025. 8(1): p. 150-155.

[10]Parra-Sánchez, J., et al., Evaluación de un programa de ejercicio físico supervisado en pacientes sedentarios mayores de 65 años con diabetes mellitus tipo 2. Atención Primaria, 2015. 47(9): p. 555-562.

[11]Ng, C.L.W., et al., Minimal difference between aerobic and progressive resistance exercise on metabolic profile and fitness in older adults with diabetes mellitus: a randomised trial. Journal of Physiotherapy, 2010. 56(3): p. 163-170.

[12]McDermott, K.A., et al., A yoga intervention for type 2 diabetes risk reduction: a pilot randomized controlled trial. BMC Complementary and Alternative Medicine, 2014. 14(1).

[13]Ma, X., et al., Effect of blood flow-restrictive resistance training on metabolic disorder and body composition in older adults with type 2 diabetes: a randomized controlled study. Frontiers in endocrinology, 2024. 15: p. 1409267.

[14]Karstoft, K., et al., The Effects of Free-Living Interval-Walking Training on Glycemic Control, Body Composition, and Physical Fitness in Type 2 Diabetic Patients. Diabetes Care, 2013. 36(2): p. 228-236.

[15]Delevatti, R.S., et al., Glucose control can be similarly improved after aquatic or dry-land aerobic training in patients with type 2 diabetes: A randomized clinical trial. Journal of Science and Medicine in Sport, 2016. 19(8): p. 688-693.

[16]Cassidy, S., et al., Unsupervised high-intensity interval training improves glycaemic control but not cardiovascular autonomic function in type 2 diabetes patients: A randomised controlled trial. Diabetes and Vascular Disease Research, 2018. 16(1): p. 69-76.

[17]Bellavere, F., et al., Effects of aerobic or resistance exercise training on cardiovascular autonomic function of subjects with type 2 diabetes: A pilot study. Nutrition, Metabolism and Cardiovascular Diseases, 2018. 28(3): p. 226-233.

[18]Annibalini, G., et al., Concurrent Aerobic and Resistance Training Has Anti-Inflammatory Effects and Increases Both Plasma and Leukocyte Levels of IGF-1 in Late Middle-Aged Type 2 Diabetic Patients. Oxidative Medicine and Cellular Longevity, 2017. 2017(1): p. 3937842.

[19]de Andrade, I.Y.T.P., et al., Pilates training reduces blood pressure in older women with type 2 diabetes: A randomized controlled trial. Journal of Bodywork and Movement Therapies, 2022. 30: p. 168-175.

[20]Agurs-Collins, T.D., et al., A Randomized Controlled Trial of Weight Reduction and Exercise for Diabetes Management in Older African-American Subjects. Diabetes Care, 1997. 20(10): p. 1503-1511.

[21]Moe, B., et al., Effects of aerobic versus resistance training on glycaemic control in men with type 2 diabetes. European Journal of Sport Science, 2011. 11(5): p. 365-374.
[22]Hwang C L, Lim J, Yoo J K, et al. Effect of all-extremity high-intensity interval training vs. moderate-intensity continuous training on aerobic fitness in middle-aged and older adults with type 2 diabetes: A randomized controlled trial[J]. Experimental gerontology, 2019, 116: 46-53.
[23]Tang, F., et al., Moderate resistance training reduces intermuscular adipose tissue and risk factors of atherosclerotic cardiovascular disease for elderly patients with type 2 diabetes. Diabetes, obesity & metabolism, 2024. 26(8): p. 3418-3428.
[24]Kadoglou, N.P.E., et al., The effects of resistance training on ApoB/ApoA-I ratio, Lp(a) and inflammatory markers in patients with type 2 diabetes. Endocrine, 2012. 42(3): p. 561-569.
[25]Hu, S., et al., The effect of tai chi intervention on NLRP3 and its related antiviral inflammatory factors in the serum of patients with pre-diabetes. Frontiers in Immunology, 2022. 13.
[26]Gupta, U., et al., Effectiveness of yoga-based exercise program compared to usual care, in improving HbA1c in individuals with type 2 diabetes: A randomized control trial. International Journal of Yoga, 2020. 13(3): p. 233.
[27]Sreedevi, A., et al., A Randomized controlled trial of the effect of yoga and peer support on glycaemic outcomes in women with type 2 diabetes mellitus: a feasibility study. BMC Complementary and Alternative Medicine, 2017. 17(1).
[28]Loimaala A, Groundstroem K, Rinne M, et al. Exercise training does not improve myocardial diastolic tissue velocities in Type 2 diabetes[J]. Cardiovascular ultrasound, 2007, 5(1): 32.
[29]Hordern, M.D., et al., Determinants of changes in blood glucose response to short-term exercise training in patients with Type 2 diabetes. Clinical Science, 2008. 115(9): p. 273-281.
[30]Mendes, R., et al., Implementing Low-Cost, Community-Based Exercise Programs for Middle-Aged and Older Patients with Type 2 Diabetes: What Are the Benefits for Glycemic Control and Cardiovascular Risk? International Journal of Environmental Research and Public Health, 2017. 14(9): p. 1057.

[31]Terauchi, Y., T. Takada and S. Yoshida, A randomized controlled trial of a structured program combining aerobic and resistance exercise for adults with type 2 diabetes in Japan. Diabetology International, 2021. 13(1): p. 75-84.

[32]Ranasinghe C, Devage S, Constantine G R, et al. Glycemic and cardiometabolic effects of exercise in South Asian Sri Lankans with type 2 diabetes mellitus: A randomized controlled trial Sri Lanka diabetes aerobic and resistance training study (SL-DARTS)[J]. Diabetes & Metabolic Syndrome: Clinical Research & Reviews, 2021, 15(1): 77-85.

[33]Bellia, A., et al., Exercise individualized by TRIMPi method reduces arterial stiffness in early onset type 2 diabetic patients: A randomized controlled trial with aerobic interval training. International Journal of Cardiology, 2017. 248: p. 314-319.

[34]Magalhães, J.P., et al., Effects of combined training with different intensities on vascular health in patients with type 2 diabetes: a 1-year randomized controlled trial. Cardiovascular Diabetology, 2019. 18(1).

[35]Amin, M., et al., Improving Metabolic Syndrome in Ghanaian Adults with Type 2 Diabetes through a Home-Based Physical Activity Program: A Feasibility Randomised Controlled Trial. International Journal of Environmental Research and Public Health, 2023. 20(8): p. 5518.

[36]Ma, X., et al., The effect of blood flow-restrictive resistance training on the risk of atherosclerotic cardiovascular disease in middle-aged patients with type 2 diabetes: a randomized controlled trial. Frontiers in Endocrinology, 2024. 15: p. 1482985.

Supplementary S3. Exploratory analysis

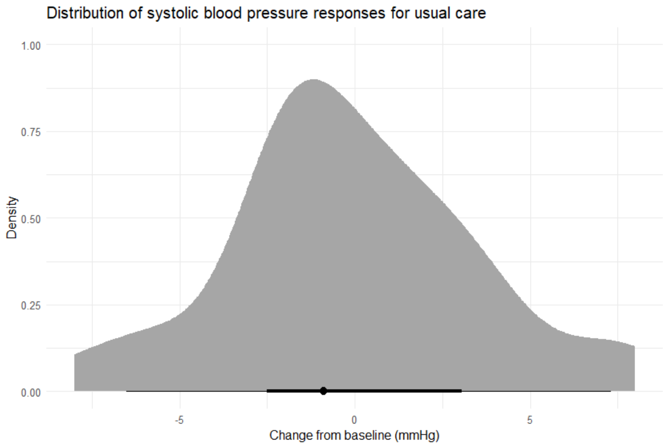

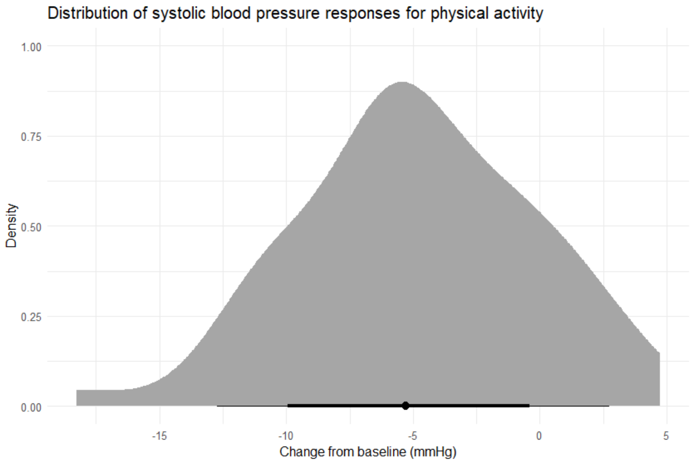

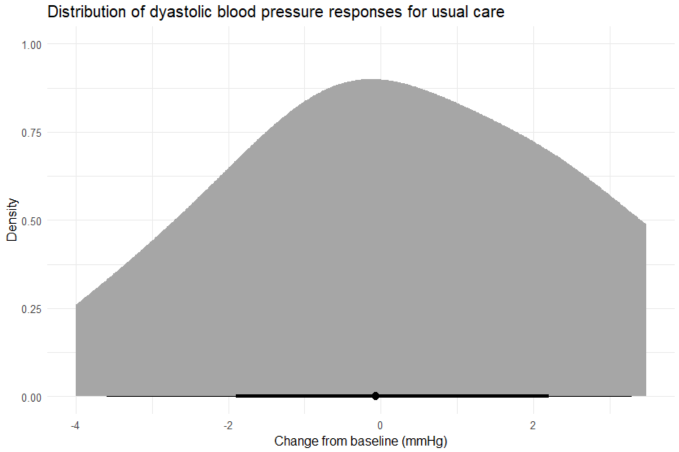

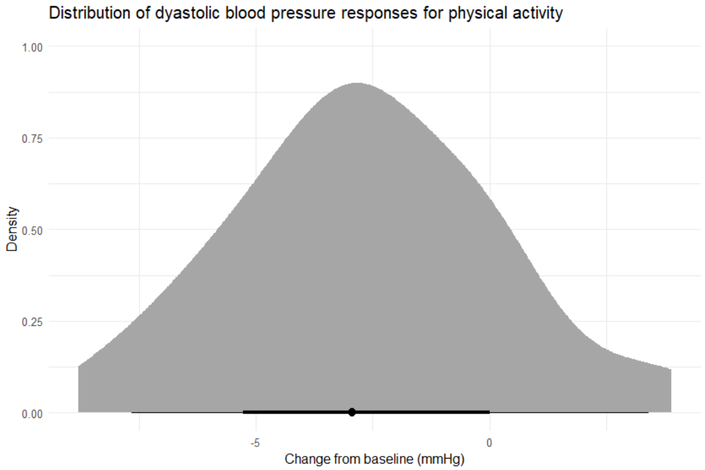

As illustrated, both systolic and diastolic blood pressure change values in the Usual Care and Physical Activity groups demonstrated an approximately symmetrical, bell-shaped distribution, with no marked skewness or extreme outliers. The data from both groups largely conformed to the assumption of normality, thereby supporting the appropriateness of employing parametric statistical methods for subsequent analyses.

Supplementary S4. Model fit comparison

Systolic blood pressure			
Model	Residual deviance (79 data points)	Number of parameters	DIC
Fixed-effect (covariate)	79	46.6	125.6
Random-effects (covariate)	71	52.9	123.9

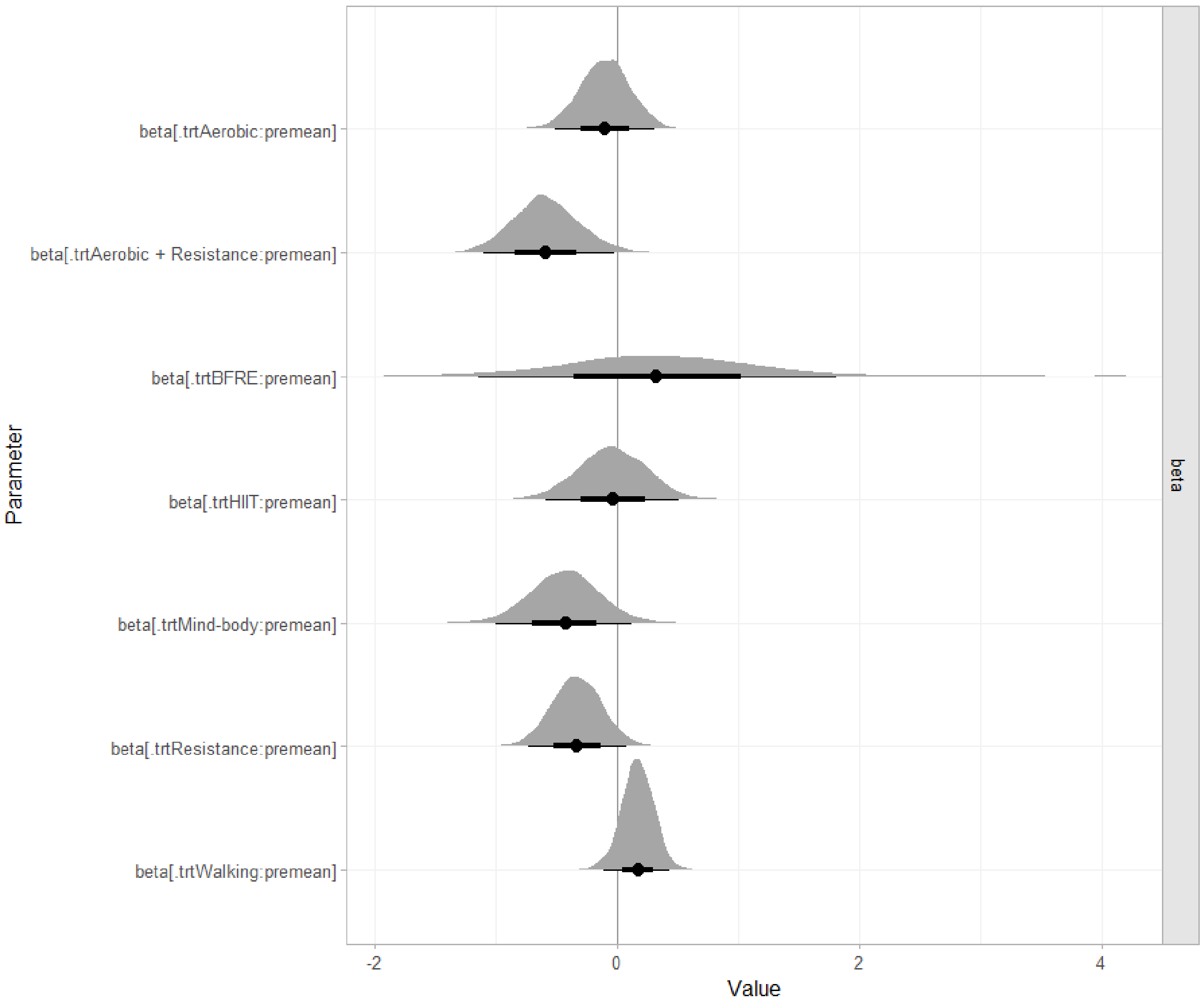

beta[.trtAerobic:premean]
beta[.trtAerobic + Resistance:premean]
beta[.trtBFRE:premean]
beta[.trtHIIT:premean]
beta[.trtMind-body:premean]
beta[.trtResistance:premean]
beta[.trtWalking:premean]
Parameter
-2
0
2
4
Value
beta

Systolic blood pressure :Halfeye plots of regression coefficients with 50% and 95% credible intervals

Diastolic blood pressure			
Model	Residual deviance (77 data points)	Number of parameters	DIC
Fixed-effect (covariate)	82	47	129
Random-effects (covariate)	75.3	52.9	128.2

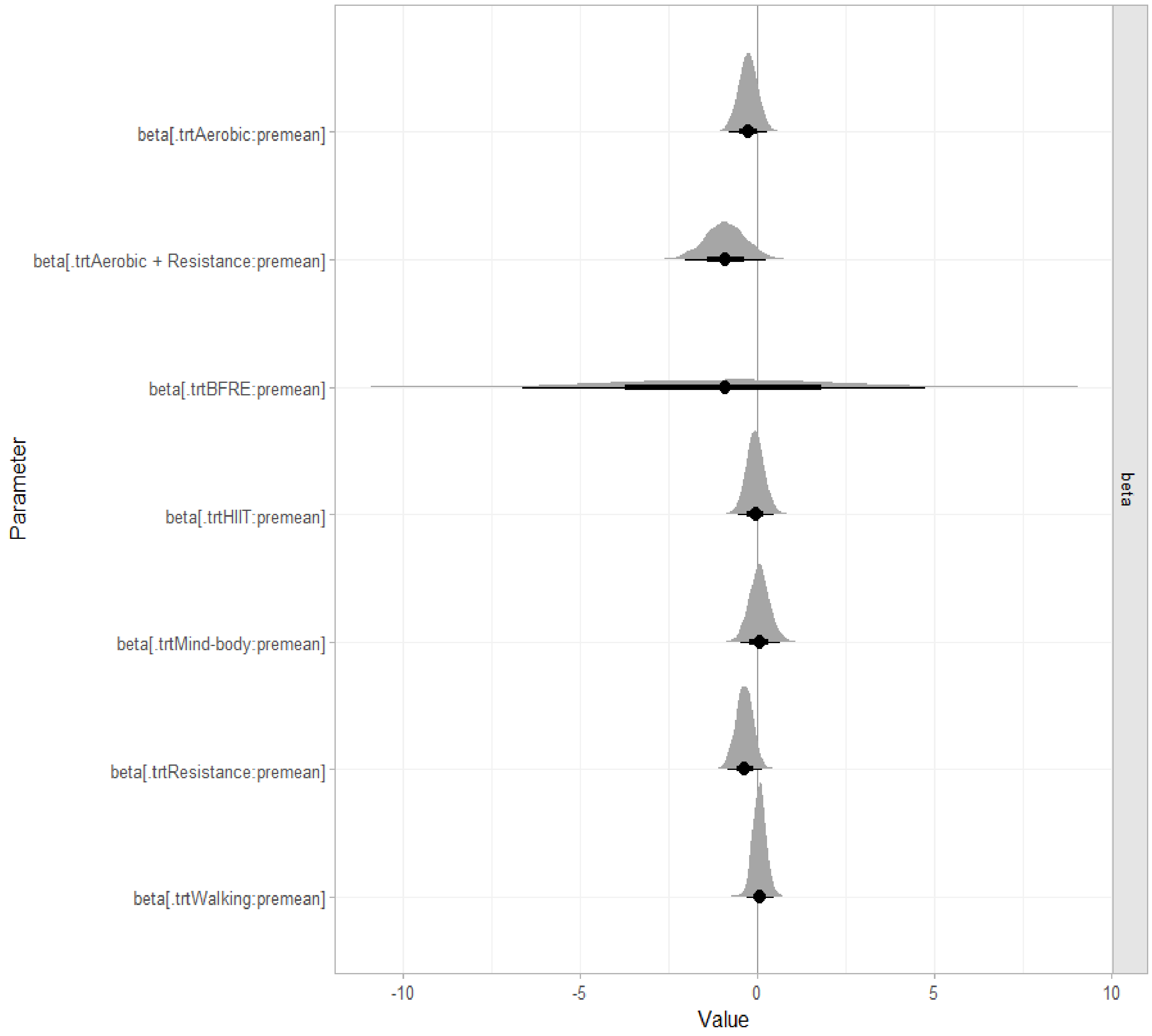

beta[.trtAerobic:premean]
beta[.trtAerobic + Resistance:premean]
beta[.trtBFRE:premean]
beta[.trtHIIT:premean]
beta[.trtMind-body:premean]
beta[.trtResistance:premean]
beta[.trtWalking:premean]
Parameter
beta
-10
-5
0
5
10
Value

Diastolic blood pressure: Halfeye plots of regression coefficients with 50% and 95% credible intervals

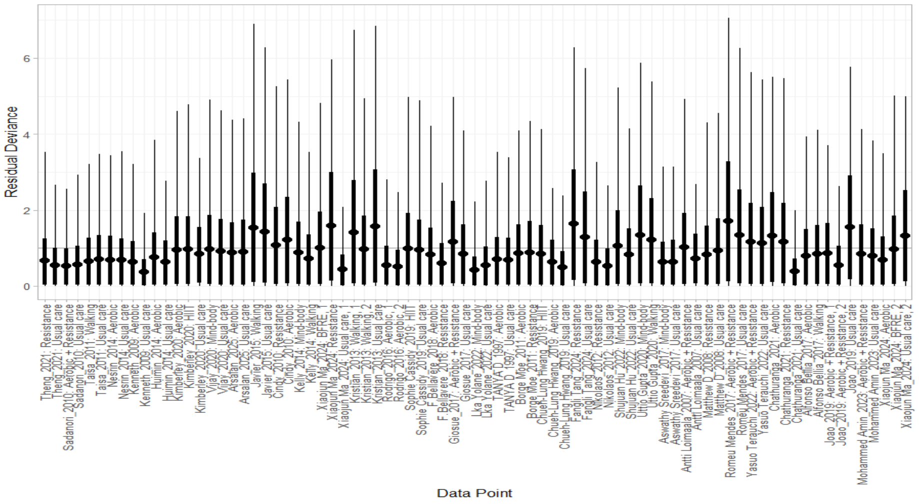

Systolic blood pressure: Diagnostic Plot of Residual Deviance Contributions for Individual Studies

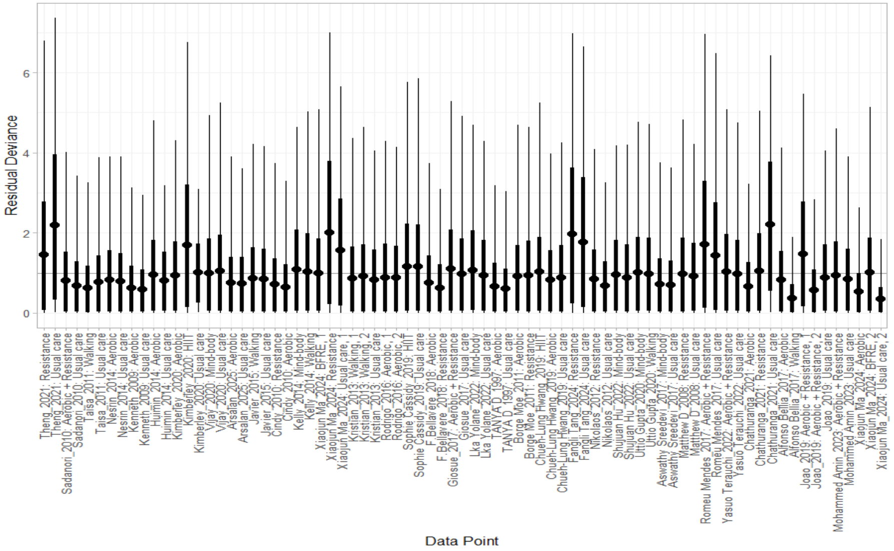

Diastolic blood pressure: Diagnostic Plot of Residual Deviance Contributions for Individual Studies

As shown in Figure , most studies show residual deviance contributions close to or below the expected value of 1, indicating adequate model fit.

Supplementary S5. Network geometry at treatment-levels.

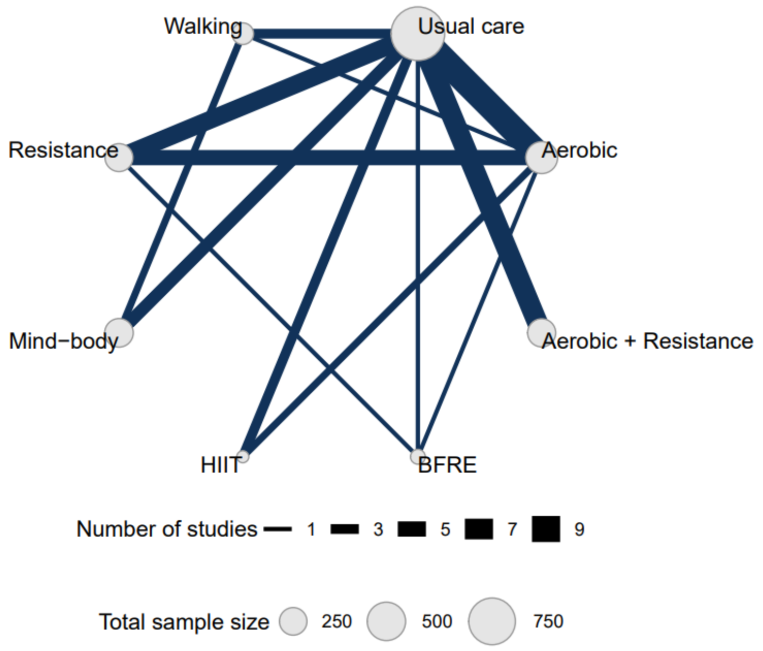

Systolic blood pressure

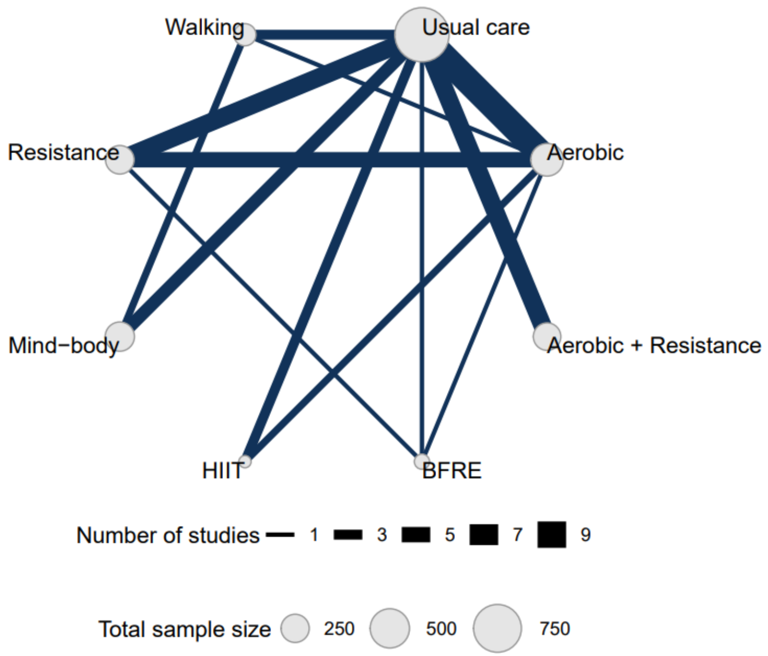

Diastolic blood pressure

Supplementary S6. Inconsistency check

Systolic blood pressure			
Model	Residual deviance (79 data points)	Number of parameters	DIC
UME	70.8	55.3	126.1
Random-effects （covariate）	71	52.9	123.9

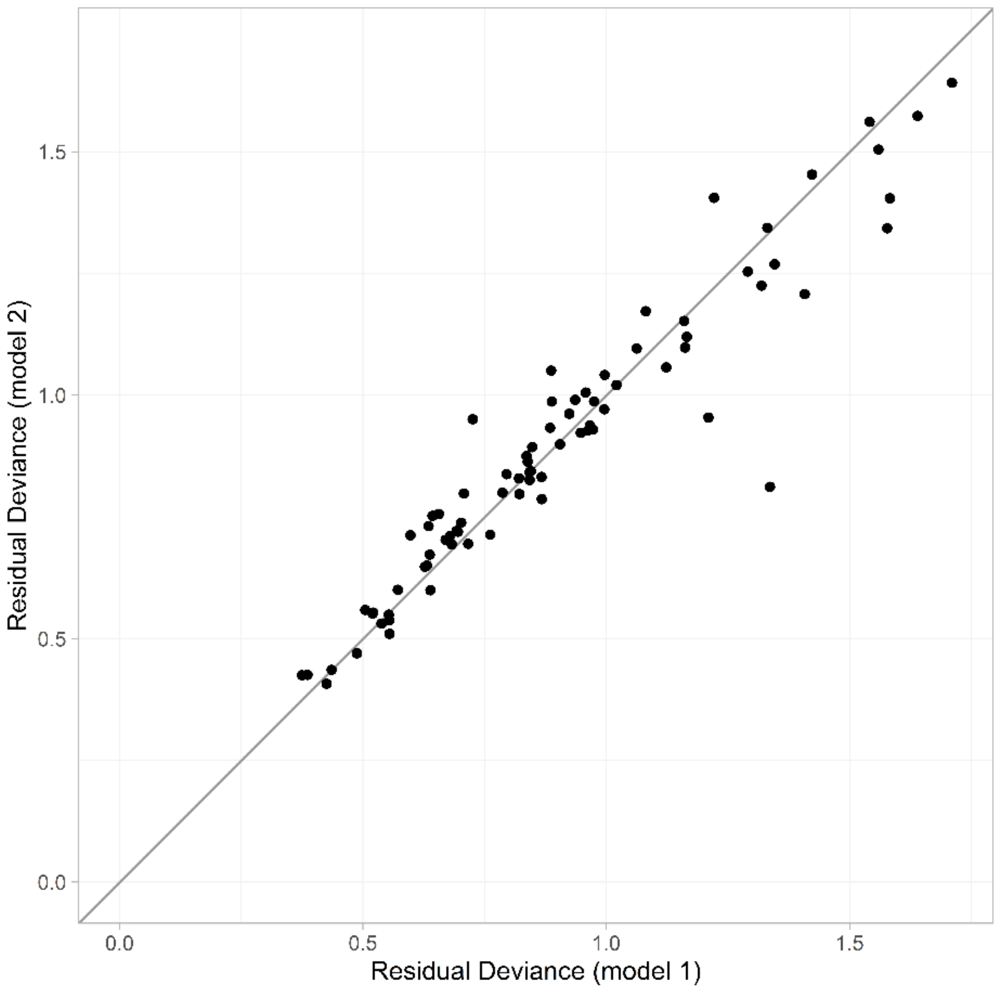

Systolic blood pressure: Dev-dev plot for consistency assessment

As shown in Figure , the majority of data points cluster closely around the reference diagonal line ($y = x$), indicating that the consistency assumption is generally tenable across the entire network. This distribution pattern suggests that for most studies, the model fit is similar under both the consistent and UME models, implying no significant conflict between direct and indirect evidence.

Diastolic blood pressure			
Model	Residual deviance (77 data points)	Number of parameters	DIC
UME	76.2	56.5	132.7
Random-effects （covariate）	75.3	52.9	128.2

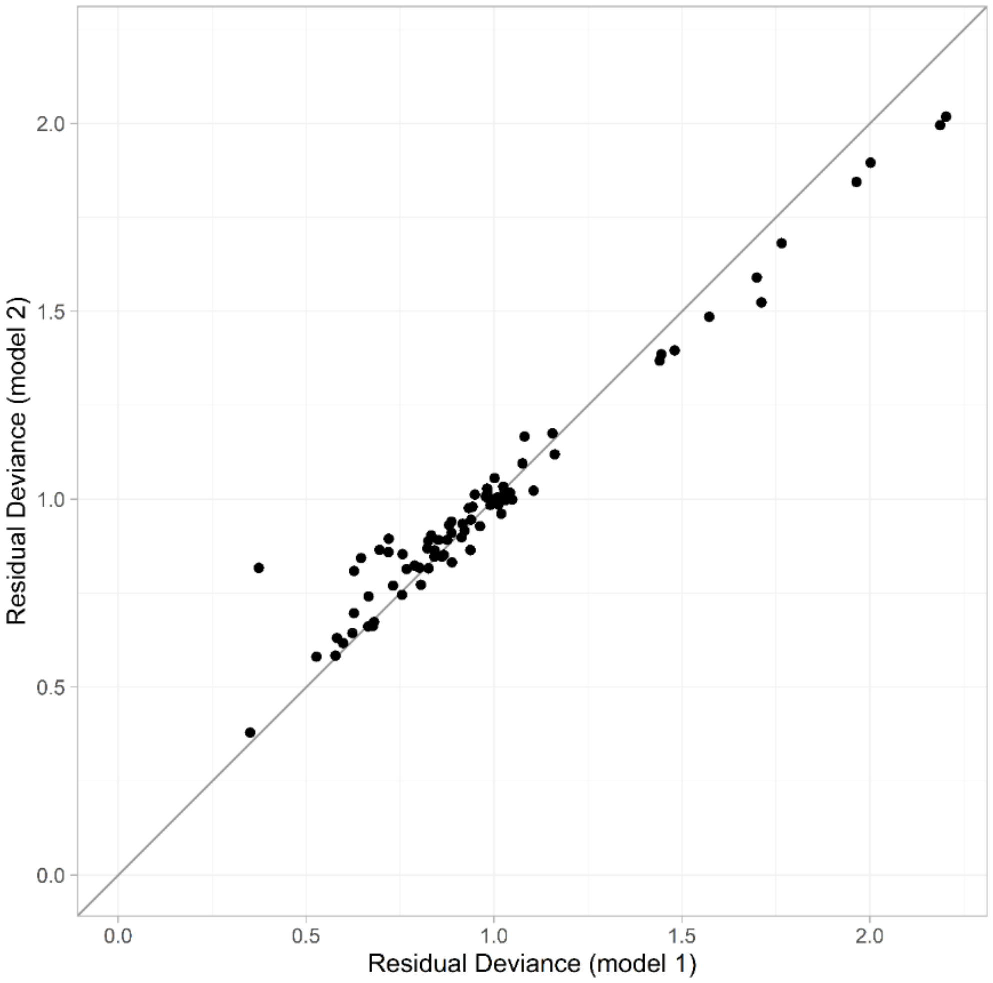

Diastolic blood pressure: Dev-dev plot for consistency assessment

As shown in Figure , the majority of data points cluster closely around the reference diagonal line (y = x), indicating that the consistency assumption is generally tenable across the entire network. This distribution pattern suggests that for most studies, the model fit is similar under both the consistent and UME models, implying no significant conflict between direct and indirect evidence.

Supplementary S7. Relative effect at treatment according to systolic blood pressure baseline.

Systolic blood pressure			
Trt	125mmHg	135mmHg	145mmHg
Aerobic	-4.29(-8.27, -0.29)*	-5.27 (-8.00, -2.34)*	-6.18 (-11.81, -0.38)*
Aerobic+Resistance	-0.35 (-5.45, 4.35)	-6.15 (-9.41, -2.78)*	-12.02 (-19.21, -4.45)*
BFRE	-5.20 (-12.77, 2.58)	-2.00 (-11.25, 7.58)	1.46 (-22.13, 24.49)
HIIT	-2.28 (-6.60, 1.96)	-2.60 (-10.18, 5.02)	-2.99 (-15.53, 9.48)
Mind-body	-4.69 (-8.52, -0.79)*	-9.00 (-14.06, -3.89)*	-13.27 (-23.39, -3.46)*
Resistance	0.08 (-3.74, 4.06)	-3.19 (-6.48, 0.15)	-6.43 (-12.89, -0.32)*
Walking	-5.13 (-9.37, -1.08)*	-3.32 (-6.55, -0.42)*	-1.54 (-5.81, 2.16)

Note: Data are presented as posterior medians (95% credible intervals); *indicates that the 95% credible interval does not include 0, representing statistical significance; negative values indicate a reduction in diastolic blood pressure.

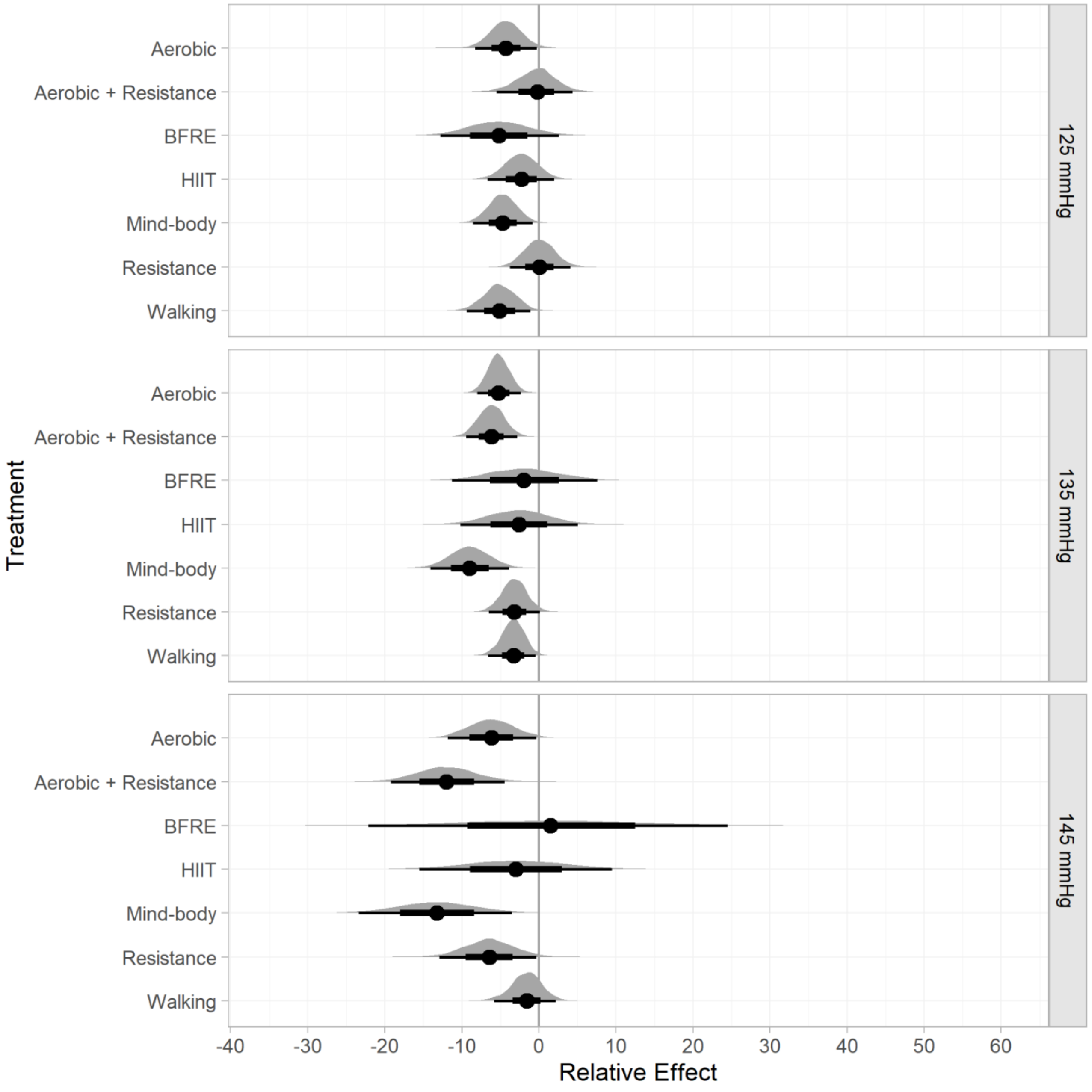

Treatment
Aerobic
Aerobic + Resistance
BFRE
HIIT
Mind-body
Resistance
Walking
125 mmHg
135 mmHg
145 mmHg
-40
-30
-20
-10
0
10
20
30
40
50
60
Relative Effect

Comparison of Intervention Effects on Systolic Blood Pressure at Different Baseline Levels

Supplementary S8. Relative effect at treatment according to diastolic blood pressure baseline.

Diastolic blood pressure			
Trt	75 mmHg	85 mmHg	90 mmHg
Aerobic	-1.91 (-4.94, 1.18)	-4.27 (-7.41, -1.11)*	-5.43 (-11.07, 0.24)
Aerobic+Resistance	1.14 (-3.95, 6.17)	-7.64 (-14.78, -0.78)*	-12.04 (-24.73, 0.15)
BFRE	0.95 (-20.65, 22.72)	-8.29 (-42.95, 27.55)	-12.77 (-76.20, 51.70)
HIIT	-2.19 (-4.95, 0.67)	-2.49 (-7.27, 2.61)	-2.71 (-9.72, 4.85)
Mind-body	-2.72 (-6.49, 0.31)	-1.99 (-5.59, 1.60)	-1.63 (-7.67, 4.72)
Resistance	0.29 (-2.23, 2.89)	-3.04 (-6.65, 0.44)	-4.75 (-10.70, 0.96)
Walking	-3.75 (-7.53, 0.08)	-2.90 (-4.94, -0.82)*	-2.46 (-5.65, 0.77)

Note: Data are presented as posterior medians (95% credible intervals); *indicates that the 95% credible interval does not include 0, representing statistical significance; negative values indicate a reduction in diastolic blood pressure.

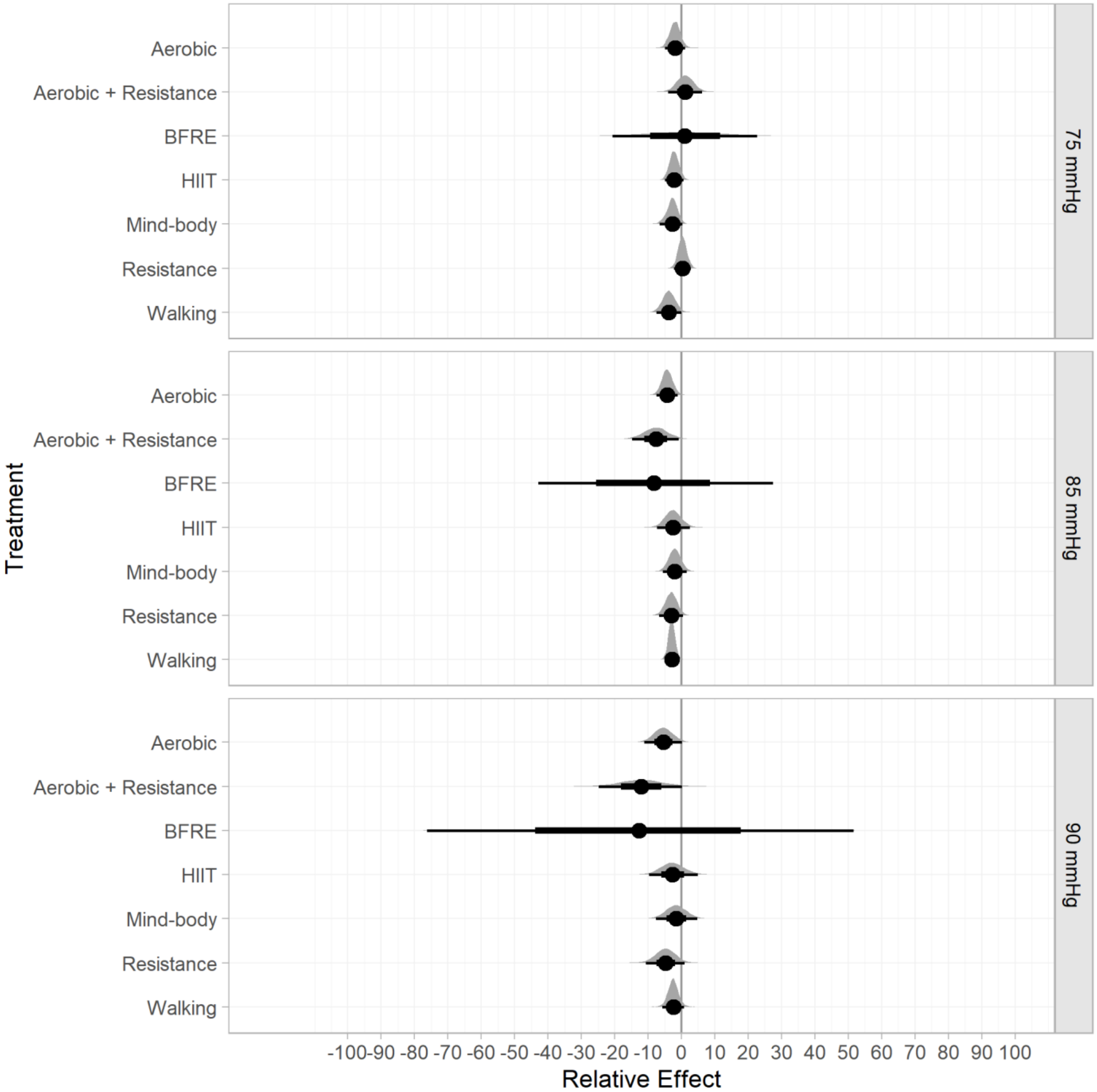

Treatment
Aerobic
Aerobic + Resistance
BFRE
HIIT
Mind-body
Resistance
Walking
75 mmHg
85 mmHg
90 mmHg
-100 -90 -80 -70 -60 -50 -40 -30 -20 -10 0 10 20 30 40 50 60 70 80 90 100
Relative Effect

Comparison of Intervention Effects on Systolic Blood Pressure at Different Baseline Levels

Supplementary S9. Meta-regression lines for each of the included interventions at treatment in systolic blood pressure.

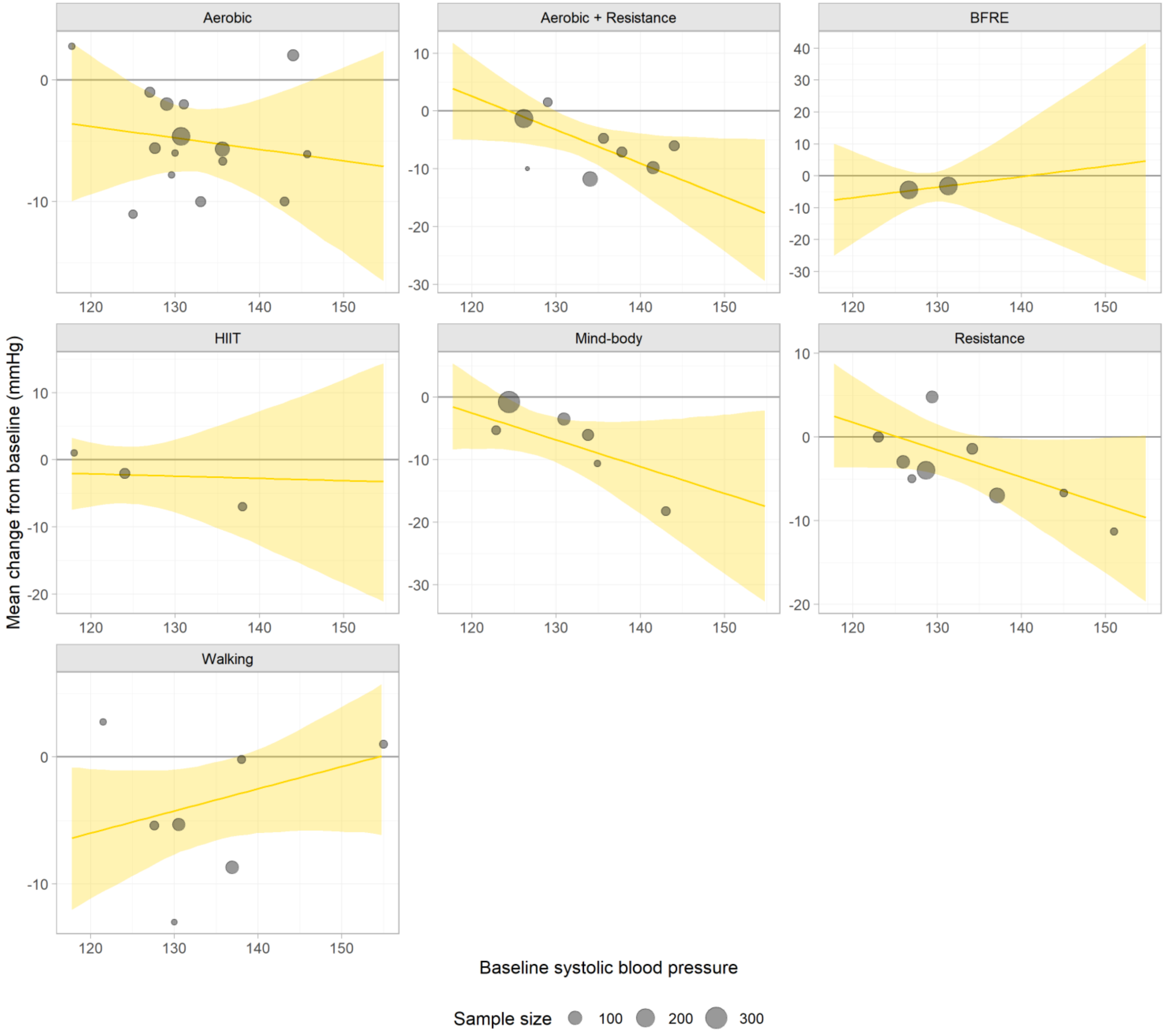

Aerobic
Aerobic + Resistance
BFRE
HIIT
Mind-body
Resistance
Walking
Mean change from baseline (mmHg)
Baseline systolic blood pressure
Sample size
100
200
300

Supplementary S10. Meta-regression lines for each of the included interventions at treatment in diastolic blood pressure.

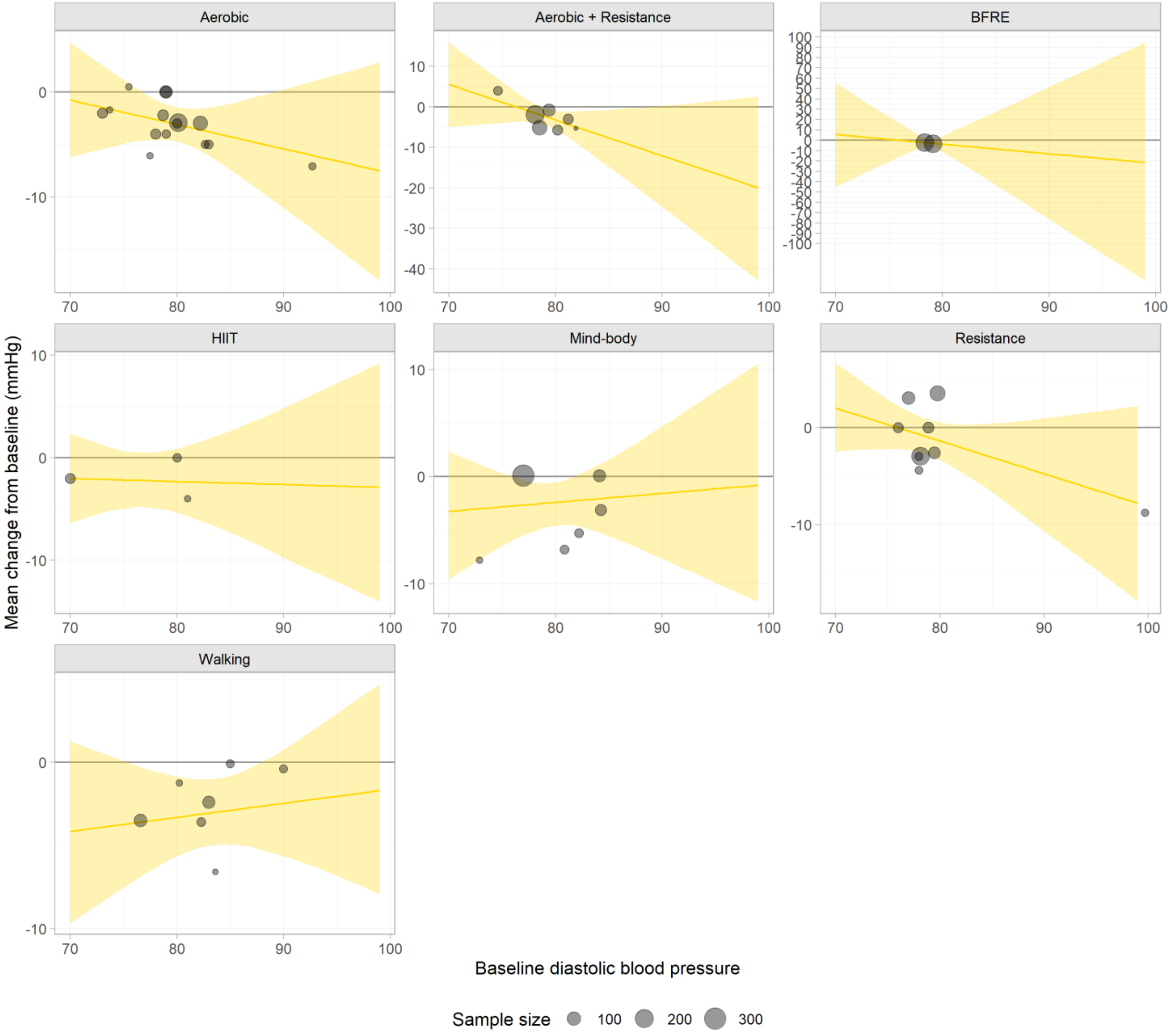

Aerobic
Aerobic + Resistance
BFRE
HIIT
Mind-body
Resistance
Walking
Mean change from baseline (mmHg)
Baseline diastolic blood pressure
Sample size
100
200
300

Supplementary S11. Treatment ranking at treatment according to systolic blood pressure baseline.

Systolic blood pressure			
Trt	125 mmHg	135 mmHg	145 mmHg
Usual care	6.75 (5.00-8.00)	7.38 (6.00-8.00)	6.88 (5.00-8.00)
Aerobic	3.15 (1.00-6.00)	3.37 (1.00-6.00)	4.02 (2.00-7.00)
Aerobic+Resistance	6.24 (2.00-8.00)	2.77 (1.00-6.00)	2.07 (1.00-5.00)
BFRE	2.95 (1.00-8.00)	5.53 (1.00-8.00)	5.99 (1.00-8.00)
HIIT	4.79 (1.00-8.00)	5.30 (1.00-8.00)	5.26 (1.00-8.00)
Mind-body	2.89 (1.00-6.00)	1.52 (1.00-5.00)	1.92 (1.00-5.00)
Resistance	6.64 (4.00-8.00)	5.13 (2.00-7.00)	3.90 (2.00-7.00)
Walking	2.60 (1.00-6.00)	4.99 (2.00-7.00)	5.96 (4.00-8.00)

Note: Values are posterior mean ranks (95% CrI). Rank 1 is best, rank 8 is worst. Parentheses show the 2.5% to 97.5% percentile.

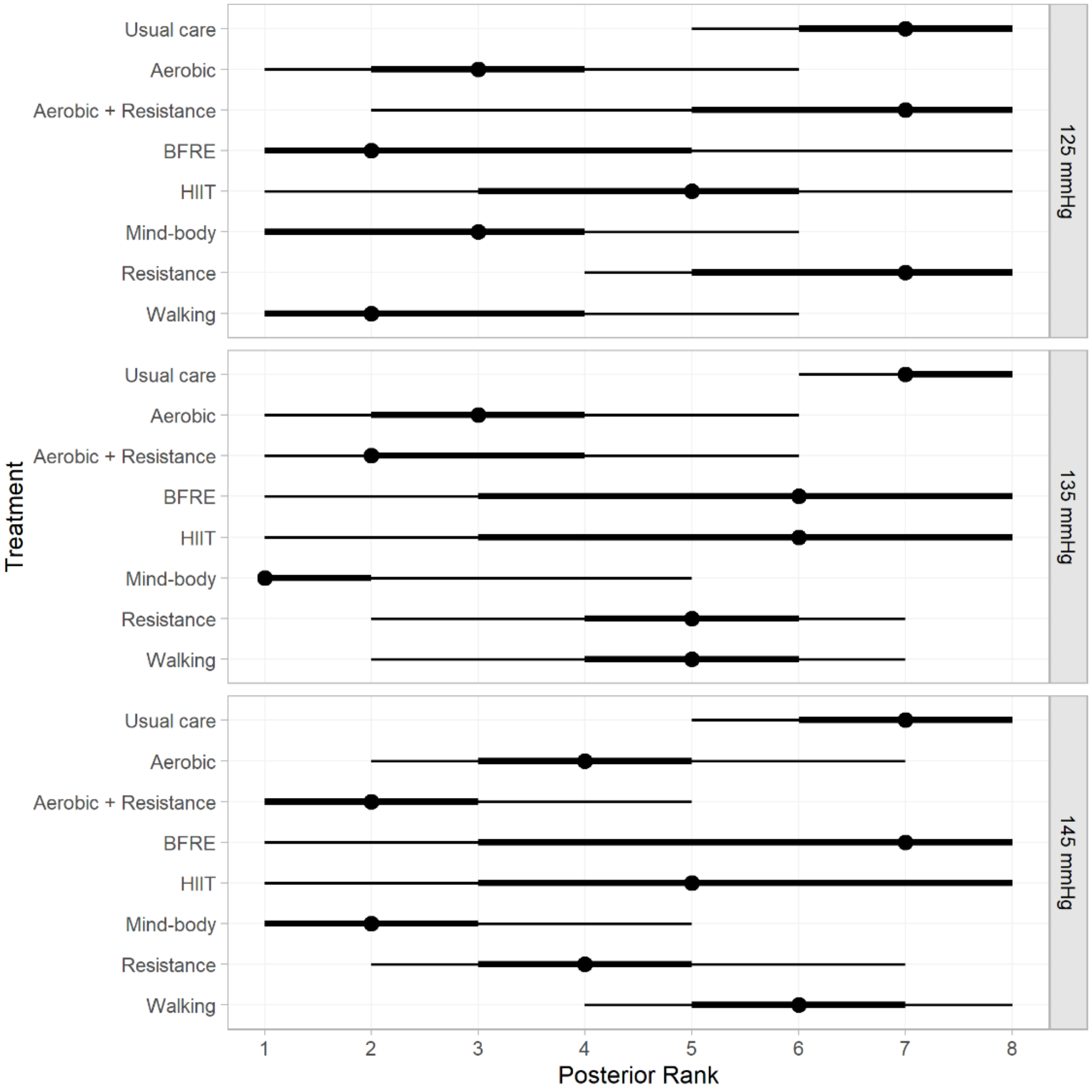

Treatment
Usual care
Aerobic
Aerobic + Resistance
BFRE
HIIT
Mind-body
Resistance
Walking
125 mmHg
135 mmHg
145 mmHg
1
2
3
4
5
6
7
8
Posterior Rank

Supplementary S12. Treatment ranking at treatment according to diastolic blood pressure baseline.

Diastolic blood pressure			
Trt	75 mmHg	85 mmHg	90 mmHg
Usual care	5.96 (4.00-8.00)	7.31 (6.00-8.00)	6.96 (5.00-8.00)
Aerobic	3.71 (1.00-7.00)	3.29 (1.00-6.00)	3.45 (1.00-7.00)
Aerobic+Resistance	6.35 (2.00-8.00)	2.13 (1.00-7.00)	2.09 (1.00-7.00)
BFRE	5.05 (1.00-8.00)	3.79 (1.00-8.00)	3.81 (1.00-8.00)
HIIT	3.47 (1.00-7.00)	4.99 (2.00-8.00)	5.09 (2.00-8.00)
Mind-body	2.96 (1.00-6.00)	5.43 (2.00-8.00)	5.64 (2.00-8.00)
Resistance	6.21 (3.00-8.00)	4.52 (2.00-7.00)	3.87 (1.00-7.00)
Walking	2.29 (1.00-6.00)	4.54 (2.00-7.00)	5.10 (2.00-7.00)

Note: Values are posterior mean ranks (95% CrI). Rank 1 is best, rank 8 is worst. Parentheses show the 2.5% to 97.5% percentile.

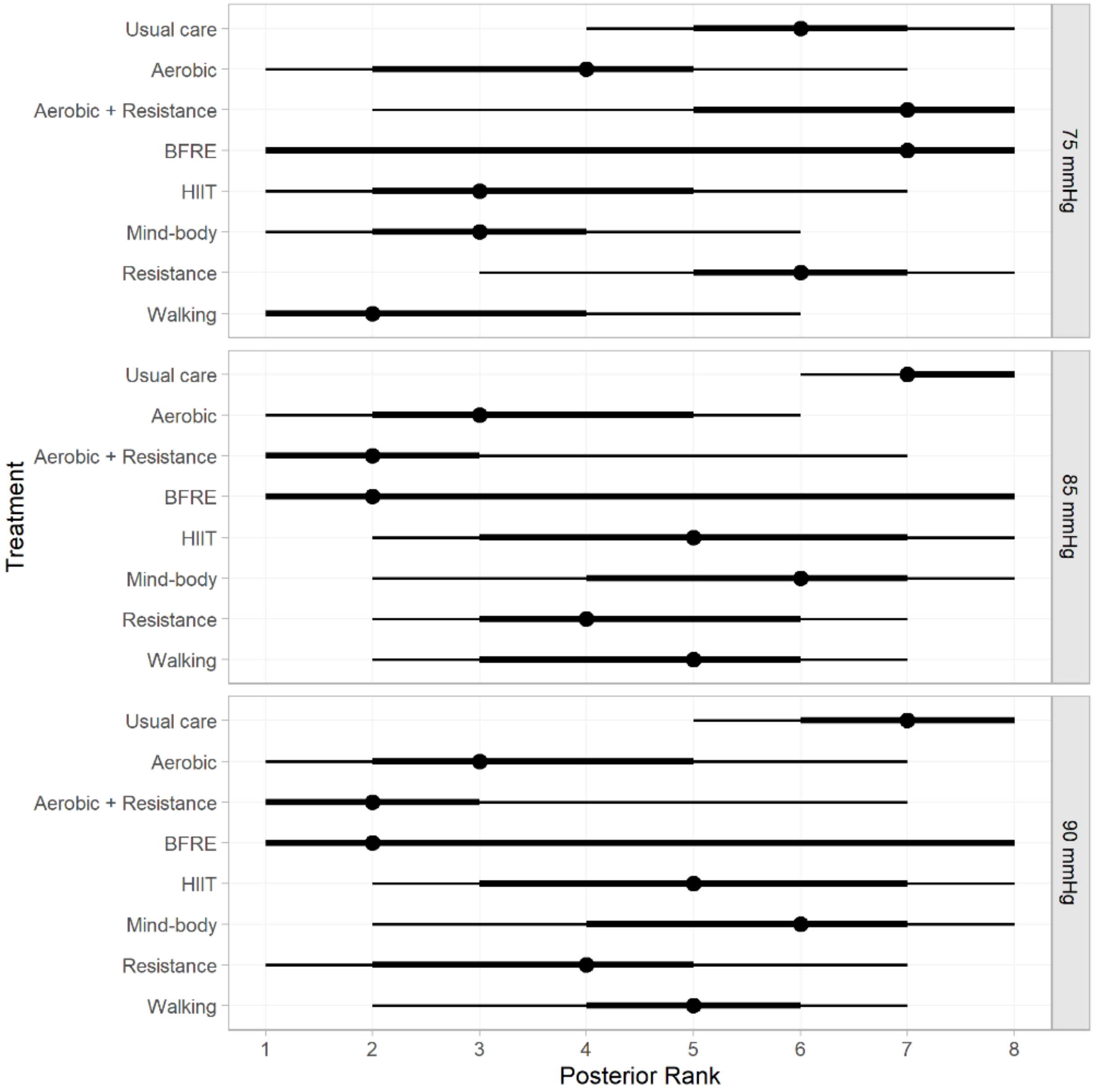

Usual care
Aerobic
Aerobic + Resistance
BFRE
HIIT
Mind-body
Resistance
Walking
75 mmHg
85 mmHg
90 mmHg
Treatment
1
2
3
4
5
6
7
8
Posterior Rank

Supplementary S13. Rank probabilities at treatment according to systolic blood pressure baseline.

**Baseline SBP:125mmHg**								
Treatment	p_rank[1]	p_rank[2]	p_rank[3]	p_rank[4]	p_rank[5]	p_rank[6]	p_rank[7]	p_rank[8]
BFRE	39%	53%	65%	77%	86%	91%	95%	100%
Walking	25%	53%	76%	90%	97%	99%	100%	100%
Mind-body	19%	46%	68%	86%	95%	98%	99%	100%
Aerobic	13%	34%	60%	84%	95%	99%	100%	100%
HIIT	3%	10%	22%	40%	68%	85%	94%	100%
Aerobic+Resistance	1%	4%	8%	16%	30%	50%	68%	100%
Resistance	0%	0%	2%	7%	20%	41%	66%	100%
Usual care	0%	0%	0%	1%	8%	37%	79%	100%
**Baseline SBP:135mmHg**								
Mind-body	70%	87%	94%	97%	99%	100%	100%	100%
Aerobic+Resistance	12%	51%	75%	89%	96%	99%	100%	100%
Aerobic	3%	24%	58%	83%	95%	99%	100%	100%
BFRE	7%	16%	24%	32%	42%	55%	71%	100%
HIIT	6%	16%	25%	34%	46%	63%	80%	100%
Walking	0%	4%	13%	33%	62%	89%	99%	100%
Resistance	0%	3%	11%	31%	58%	85%	98%	100%
Usual care	0%	0%	0%	0%	0%	9%	52%	100%
**Baseline SBP:145mmHg**								
Mind-body	49%	78%	89%	95%	98%	99%	100%	100%
Aerobic+Resistance	35%	74%	89%	96%	99%	100%	100%	100%
Resistance	2%	12%	39%	71%	89%	97%	100%	100%

Aerobic	2%	10%	35%	66%	89%	97%	99%	100%
BFRE	8%	14%	21%	28%	36%	42%	51%	100%
HIIT	4%	12%	24%	36%	52%	65%	81%	100%
Walking	0%	0%	2%	8%	32%	69%	93%	100%
Usual care	0%	0%	0%	0%	5%	30%	76%	100%

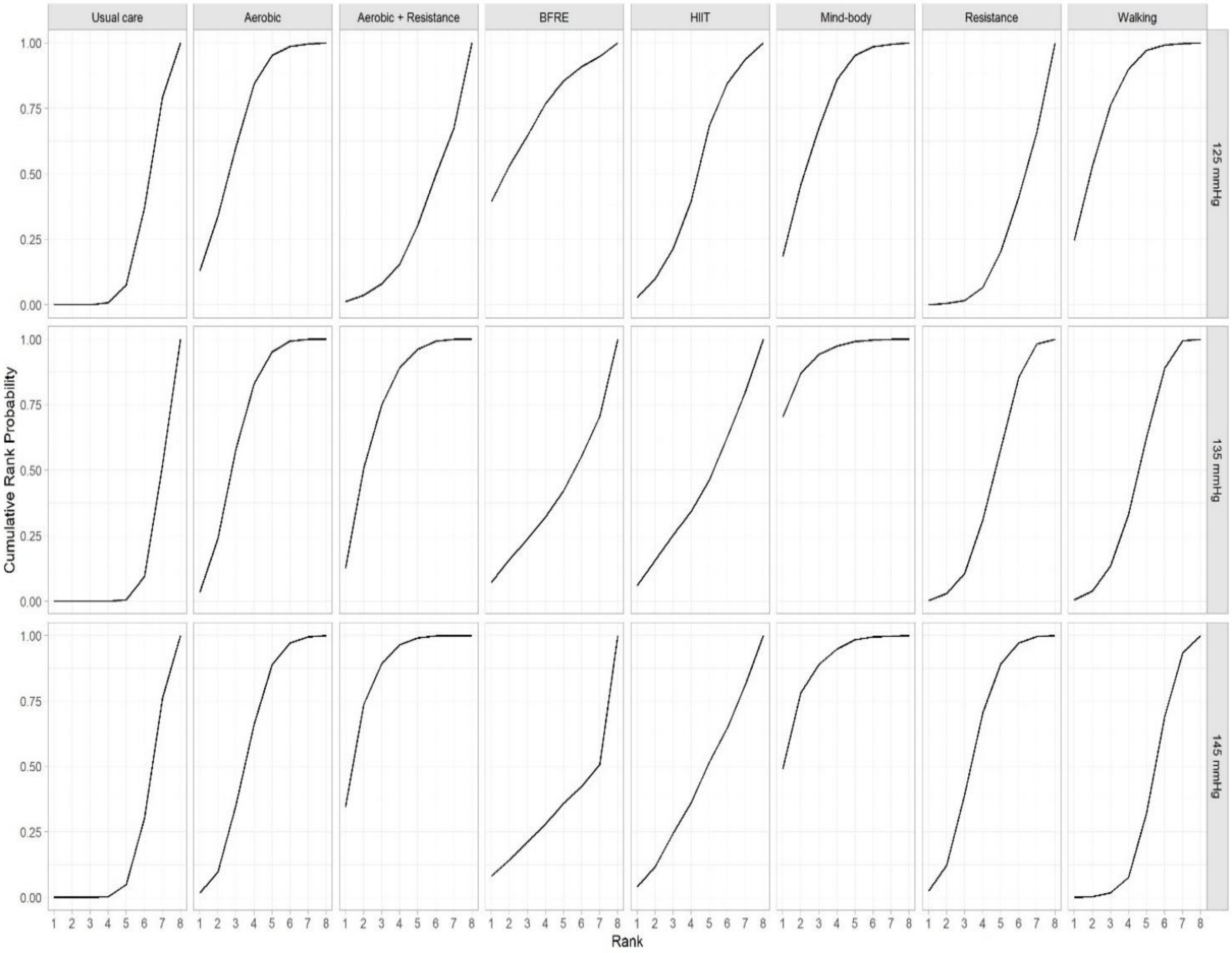

Usual care
Aerobic
Aerobic + Resistance
BFRE
HIIT
Mind-body
Resistance
Walking
125 mmHg
135 mmHg
145 mmHg
Cumulative Rank Probability
Rank

Supplementary S14. Rank probabilities at treatment according to diastolic blood pressure baseline.

**Baseline DBP:75mmHg**								
Treatment	p_rank[1]	p_rank[2]	p_rank[3]	p_rank[4]	p_rank[5]	p_rank[6]	p_rank[7]	p_rank[8]
Walking	37%	67%	81%	91%	96%	99%	100%	100%
BFRE	31%	36%	39%	42%	46%	48%	54%	100%
Mind-body	17%	45%	67%	84%	94%	98%	99%	100%
HIIT	7%	25%	54%	79%	92%	97%	99%	100%
Aerobic	6%	22%	46%	71%	89%	96%	99%	100%
Aerobic+Resistance	1%	5%	9%	16%	26%	38%	69%	100%
Resistance	0%	1%	3%	9%	26%	55%	86%	100%
Usual care	0%	0%	1%	7%	32%	70%	94%	100%
**Baseline DBP:85mmHg**								
BFRE	50%	57%	59%	61%	62%	65%	67%	100%
Aerobic+Resistance	39%	80%	87%	91%	94%	97%	99%	100%
Aerobic	5%	29%	63%	82%	93%	99%	100%	100%
HIIT	2%	12%	26%	40%	56%	73%	90%	100%
Resistance	1%	10%	27%	52%	72%	88%	98%	100%
Walking	1%	7%	23%	46%	75%	94%	100%	100%
Mind-body	1%	5%	15%	27%	46%	70%	92%	100%

Usual care	0%	0%	0%	0%	2%	14%	54%	100%
**Baseline DBP:90mmHg**								
BFRE	50%	57%	59%	61%	62%	64%	66%	100%
Aerobic+Resistance	40%	82%	87%	92%	95%	97%	98%	100%
Aerobic	4%	25%	58%	81%	91%	96%	99%	100%
Resistance	3%	17%	46%	70%	85%	94%	98%	100%
HIIT	2%	10%	23%	39%	58%	72%	88%	100%
Mind-body	1%	5%	15%	26%	43%	63%	83%	100%
Walking	0%	3%	12%	30%	60%	86%	98%	100%
Usual care	0%	0%	0%	1%	7%	28%	69%	100%

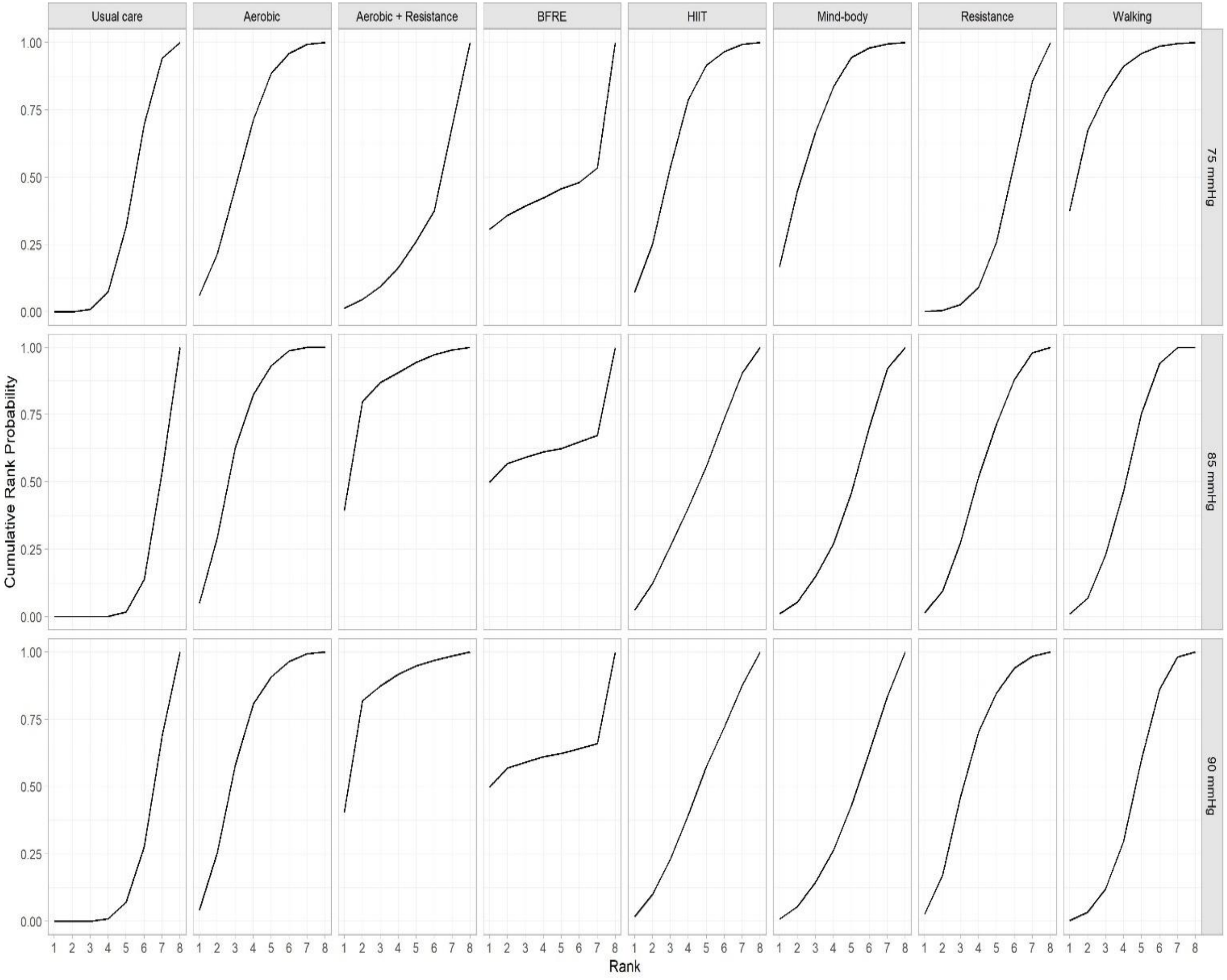

Usual care
Aerobic
Aerobic + Resistance
BFRE
HIIT
Mind-body
Resistance
Walking
75 mmHg
85 mmHg
90 mmHg
Cumulative Rank Probability
0.00
0.25
0.50
0.75
1.00
1 2 3 4 5 6 7 8
Rank

Supplementary S15. Predictive probabilities that a new trial shows no or adverse effects applying different interventions on people with different baseline SBP levels.

**Systolic blood pressure**			
Trt	125 mmHg	135 mmHg	145 mmHg
BFRE	11.3%	34.0%	54.8%
Mind-body	4.5%	0.5%	1.0%
Aerobic+Resistance	47.6%	1.6%	0.6%
HIIT	20.3%	27.3%	33.1%
Resistance	50.7%	10.1%	4.7%
Aerobic	6.6%	2.7%	4.8%
Walking	3.5%	7.3%	26.4%

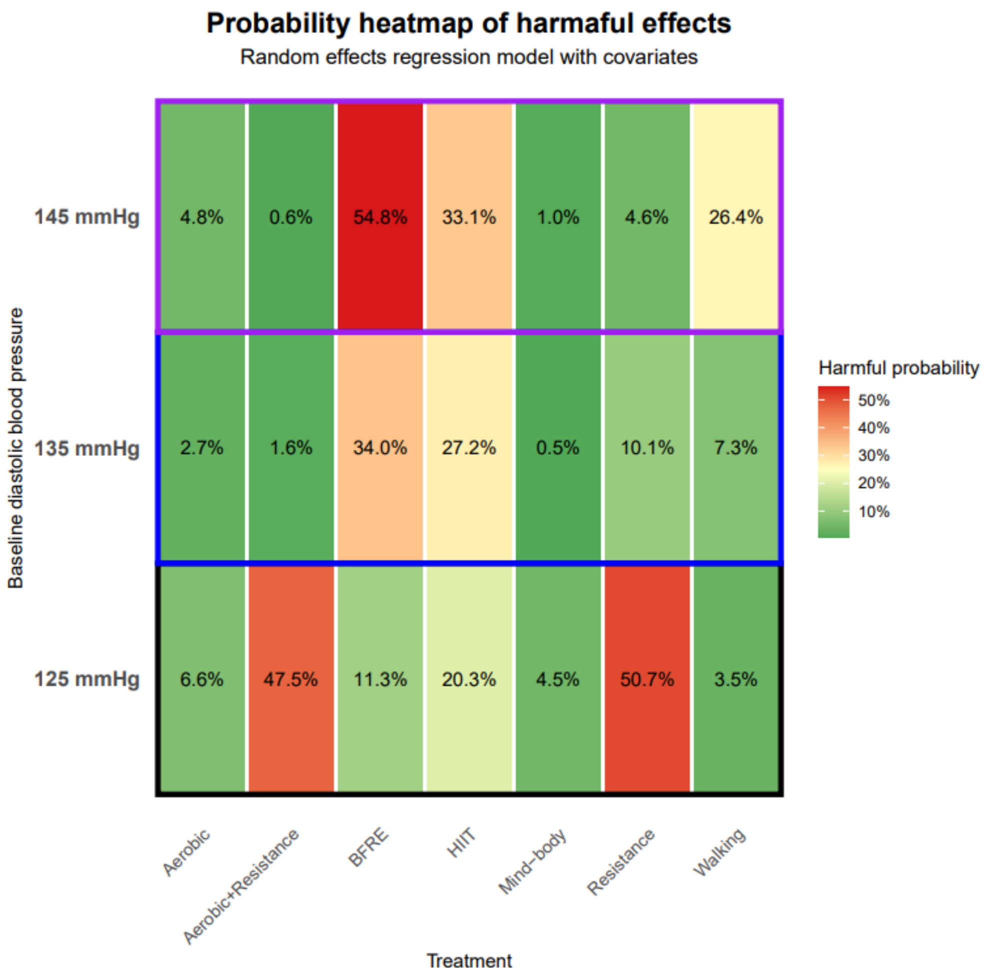
Probability heatmap of harmaful effects
Random effects regression model with covariates
Baseline diastolic blood pressure
145 mmHg
135 mmHg
125 mmHg
4.8%
0.6%
54.8%
33.1%
1.0%
4.6%
26.4%
2.7%
1.6%
34.0%
27.2%
0.5%
10.1%
7.3%
6.6%
47.5%
11.3%
20.3%
4.5%
50.7%
3.5%
Aerobic
Aerobic+Resistance
BFRE
HIIT
Mind–body
Resistance
Walking
Treatment
Harmful probability
50%
40%
30%
20%
10%

Supplementary S16. Predictive probabilities that a new trial shows no or adverse effects applying different interventions on people with different baseline DBP levels.

Diastolic blood pressure			
Trt	75 mmHg	85 mmHg	90 mmHg
BFRE	53.2%	33.3%	35.1%
Mind-body	6.8%	16.6%	31.7%
Aerobic + Resistance	66.2%	2.1%	2.9%
HIIT	11.6%	18.1%	23.7%
Resistance	57.9%	7.5%	6.4%
Aerobic	15.9%	2.0%	4.2%
Walking	4.9%	4.0%	9.2%

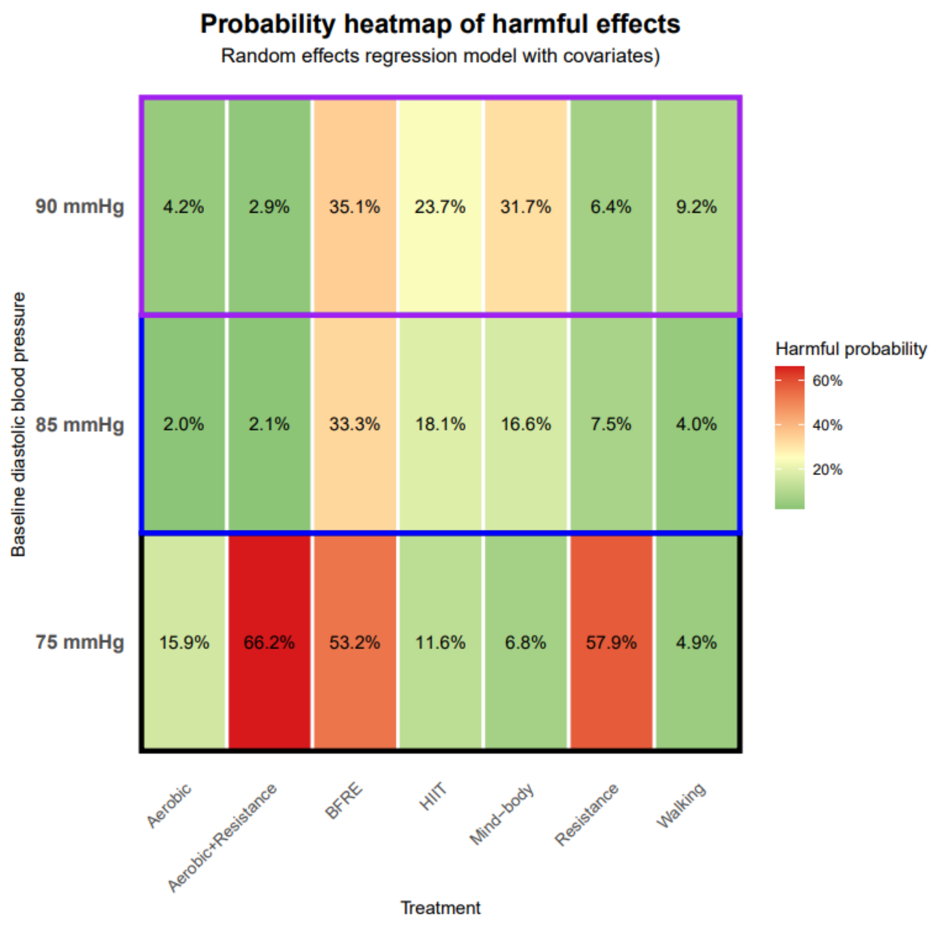

Probability heatmap of harmful effects
Random effects regression model with covariates)
Baseline diastolic blood pressure
90 mmHg
85 mmHg
75 mmHg
4.2%
2.9%
35.1%
23.7%
31.7%
6.4%
9.2%
2.0%
2.1%
33.3%
18.1%
16.6%
7.5%
4.0%
15.9%
66.2%
53.2%
11.6%
6.8%
57.9%
4.9%
Aerobic
Aerobic+Resistance
BFRE
HIIT
Mind–body
Resistance
Walking
Treatment
Harmful probability
60%
40%
20%

Supplementary S17. Comparison of linear and nonlinear dose-response models

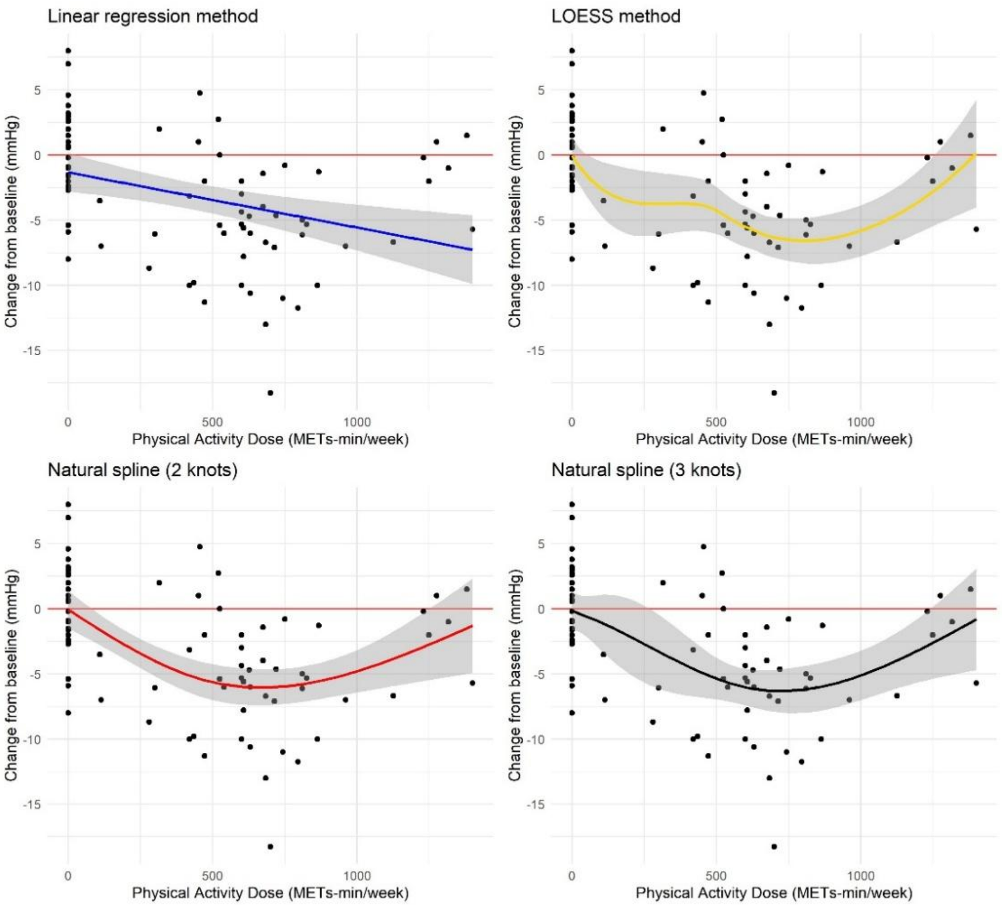

Exploration of Dose-Response Relationships between Physical Activity Dose and Systolic Blood Pressure Reduction Using Different Regression Methods

We explored the dose-response relationship between physical activity dose (measured as weekly energy expenditure in METs-min) and systolic blood pressure reduction using four different smoothing methods: linear regression, LOESS (locally estimated scatterplot smoothing), and natural splines with 2 and 3 knots. This multi-model approach allowed us to assess whether the relationship was linear or nonlinear, and to identify potential threshold effects.

Systabolic pressure blood						
Comparison	Model 1	Model 2	Δdf	ΔRSS	F-value	p-value
Test 1	Linear model	2-df spline model	1	318.88	18.848	4.313×10^{-5}
Test 2	Linear model	3-df spline model	2	329.25	9.6804	1.820×10^{-4}

Supplementary S18. Grouping analysis of systolic blood pressure dose

Analysis of variance (ANOVA) was used to test whether the mean of the dependent variable y (change in systolic blood pressure) differed significantly across dose groups. The ANOVA results showed a significant difference among groups: $F = 11.67$, $p < 0.001$ (highly significant).

Systolic blood pressure			
Dose Group	Dose Range	Sample Label	Clinical Significance
Usual Care	≤0	Usual Care	placebo control
Low Dose	0-500	Low	Low-intensity intervention
Medium Dose	500-1000	Medium	Moderate-intensity intervention
High Dose	>1000	High	High-intensity intervention

Post hoc pairwise comparisons were conducted using Tukey's Honestly Significant Difference (HSD) test to identify which specific dose groups differed significantly from each other.

Systolic blood pressure			
Comparison	Mean Difference	95% Confidence Interval	Adjusted p-value

Low vs Usual Care	-4.50	[-8.18, -0.83]	0.00996
Medium vs Usual Care	-6.07	[-8.85, -3.29]	1.1×10^{-6}
High vs Usual Care	-1.89	[-6.41, 2.62]	0.69015
Medium vs Low	-1.56	[-5.24, 2.11]	0.67925
High vs Low	2.61	[-2.50, 7.73]	0.53908
High vs Medium	4.18	[-0.34, 8.69]	0.07985

Result:

Effect ranking: Medium dose > Low dose > High dose ≈ Usual Care; The medium dose showed the best effect, reducing systolic blood pressure by 6.07 mmHg compared to usual care (highly significant); The low dose ranked second, with a reduction of 4.50 mmHg versus usual care (significant); The high dose did not show a significant effect, with no statistically meaningful difference compared to usual care.

Supplementary S19. Dose-response relationship between weekly physical activity and systolic blood pressure.

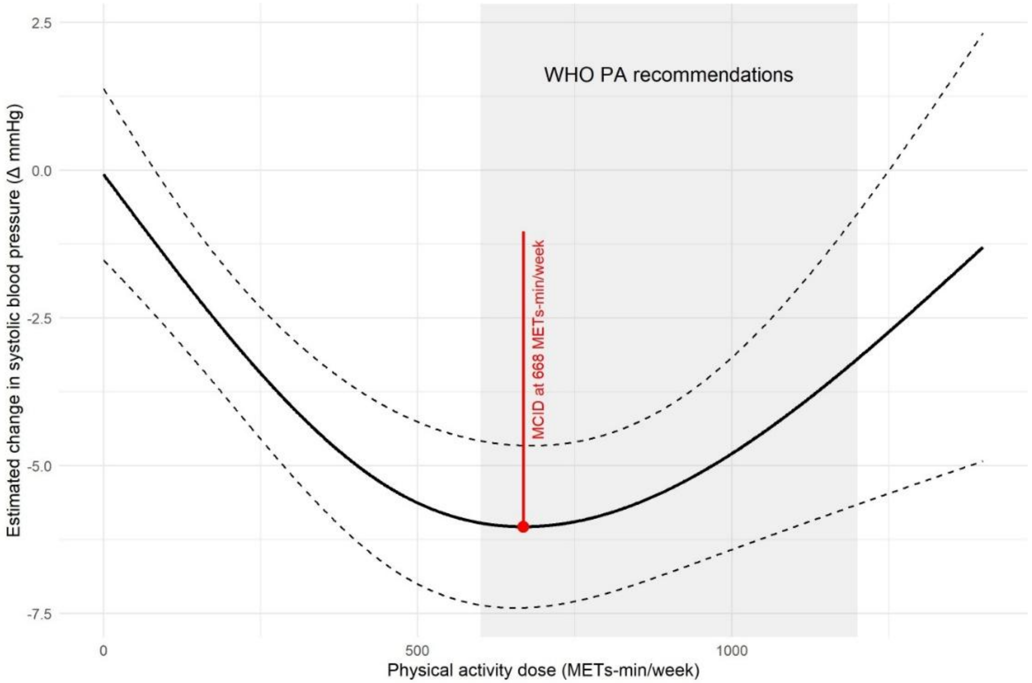

Dose-response relationship between weekly physical activity and systolic blood pressure. Solid line represents the posterior mean estimate, and dashed lines indicate 95% credible intervals (CrI) from the Bayesian model. The vertical red line shows the predicted minimum clinically important difference (MCID) at 668 MET-min/week. The shaded area corresponds to the WHO-recommended range (600-1200 MET-min/week)

Supplementary S20. Dose response relationship of different exercises

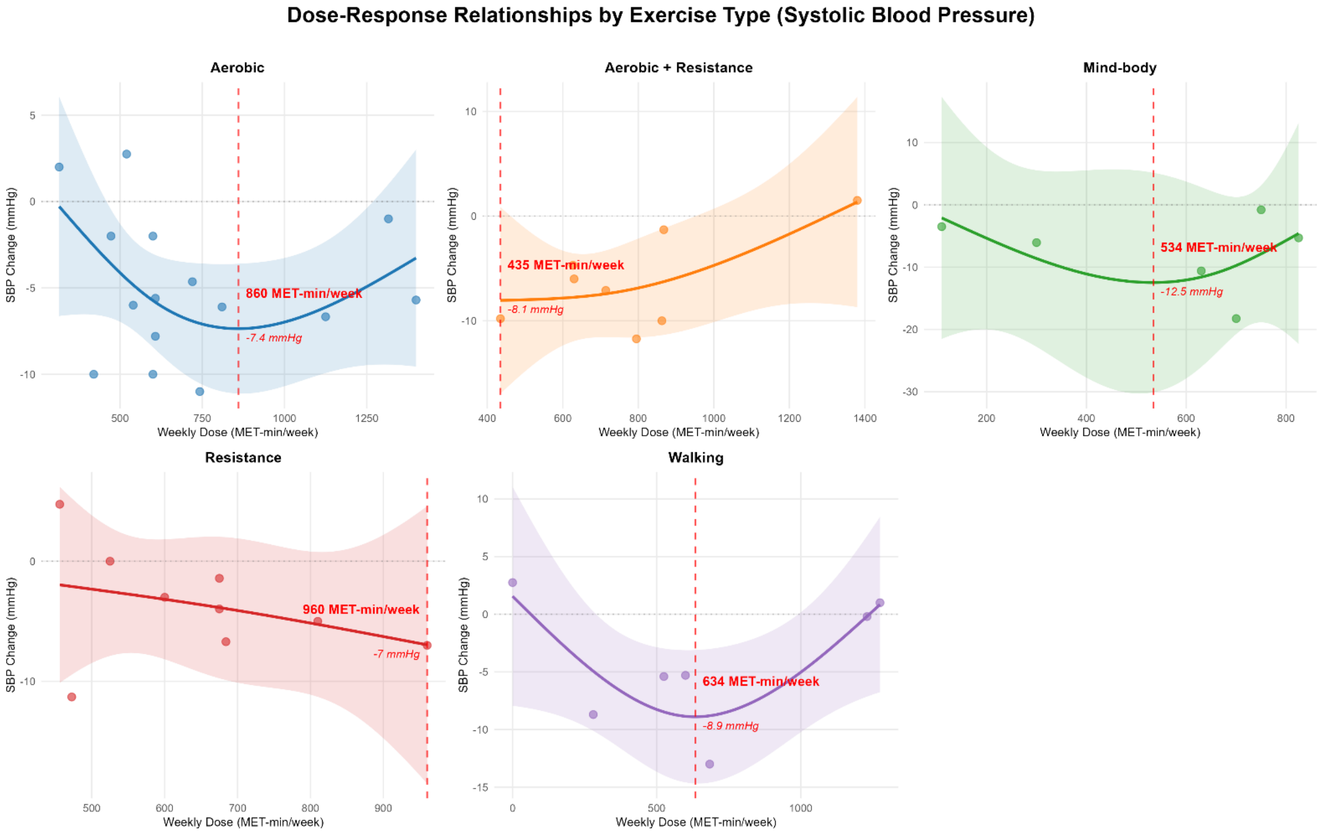

Note: Red dashed line indicates optimal dose for maximal systolic blood pressure reduction.

As illustrated in the Figure, the 95% confidence intervals for the effect sizes on systolic blood pressure for Resistance training, Aerobic+Resistance

combined training, Mind-body exercise, and Resistance training (when considered separately) all include the null value (zero), indicating a lack of statistical significance. Consequently, the analysis allows us to conclude only that the optimal doses for Aerobic exercise and Walking are 860 MET-min/week (corresponding to a reduction of -7.4 mmHg) and 634 MET-min/week (corresponding to a reduction of -8.9 mmHg), respectively.

Supplementary S21. Comparison of linear and nonlinear dose-response models

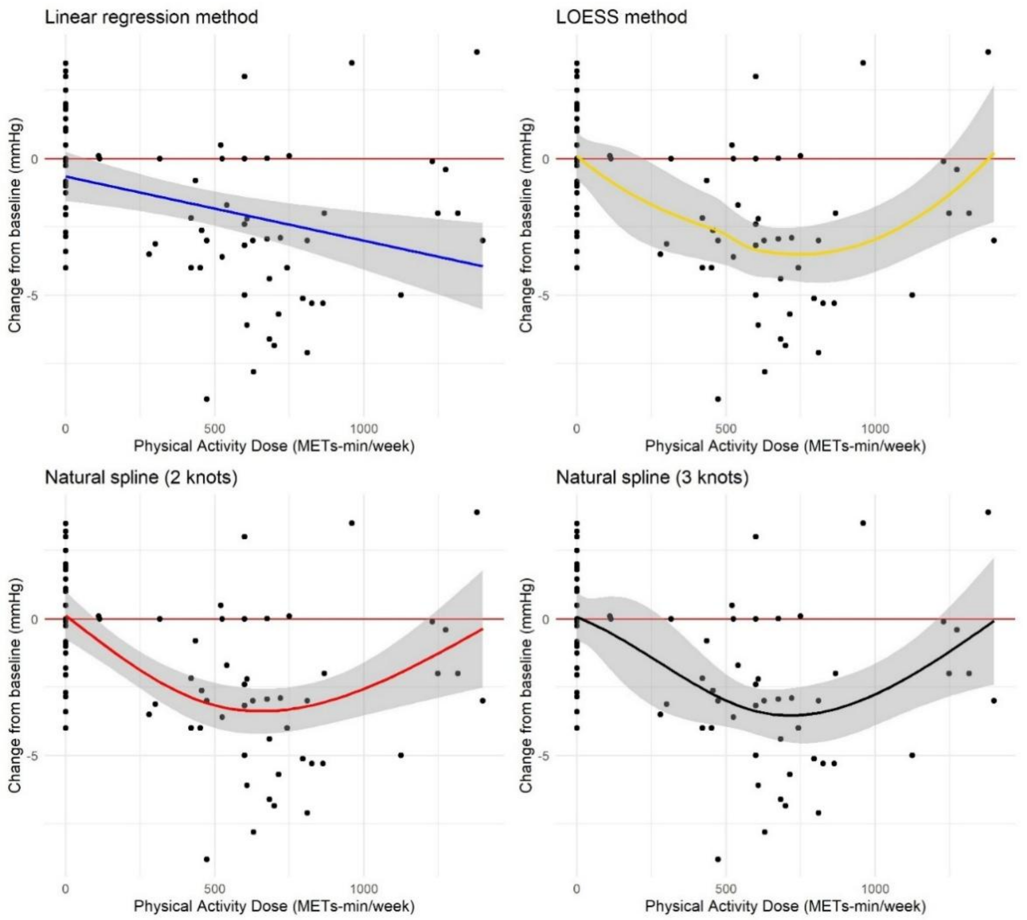

Exploration of Dose-Response Relationships between Physical Activity Dose and Systolic Blood Pressure Reduction Using Different Regression Methods

We explored the dose-response relationship between physical activity dose (measured as weekly energy expenditure in METs-min) and systolic blood pressure reduction using four different smoothing methods: linear regression, LOESS (locally estimated scatterplot smoothing), and natural splines with 2 and 3 knots. This multi-model approach allowed us to assess whether the relationship was linear or nonlinear, and to identify potential threshold effects.

Diastabolic pressure blood						
Comparison	Model 1	Model 2	Δdf	ΔRSS	F-value	p-value
Test 1	Linear model	2-df spline model	1	115.91	19.5	3.75×10^{-5}
Test 2	Linear model	3-df spline model	2	118.5	9.8917	1.58×10^{-4}

Supplementary S22. Grouping analysis of diastolic blood pressure dose

Analysis of variance (ANOVA) was used to test whether the mean of the dependent variable y (change in diastolic blood pressure) differed significantly across dose groups. The ANOVA results showed a significant difference among groups: $F = 8.1$, $p < 0.001$ (highly significant).

Diastolic blood pressure			
Dose Group	Dose Range	Sample Label	Clinical Significance
Usual Care	≤0	Usual Care	placebo control
Low Dose	0-500	Low	Low-intensity intervention
Medium Dose	500-1000	Medium	Moderate-intensity intervention
High Dose	>1000	High	High-intensity intervention

Post hoc pairwise comparisons were conducted using Tukey's Honestly Significant Difference (HSD) test to identify which specific dose groups differed significantly from each other.

Diastolic blood pressure			
Comparison	Mean Difference	95% Confidence Interval	Adjusted p-value
Low vs Usual Care	-2.718	[-5.010, -0.426]	0.0136

Medium vs Usual Care	-3.129	[-4.882, -1.375]	0.72×10^{-6}
High vs Usual Care	-1.286	[-4.097, 1.526]	0.6274
Medium vs Low	-0.410	[-2.702, 1.881]	0.9652
High vs Low	1.432	[-1.743, 4.608]	0.6376
High vs Medium	1.843	[-0.969, 4.655]	0.3192

Effect ranking: Medium dose > Low dose > High dose ≈ Usual Care; The medium dose showed the strongest effect, reducing diastolic blood pressure by 3.129 mmHg compared to usual care (highly significant, $p < 0.001$); The low dose ranked second, with a reduction of 2.72 mmHg versus usual care (significant, $p = 0.0136$); The high dose did not show a significant effect, with no statistically meaningful difference compared to usual care ($p = 0.6274$).

Supplementary S23. Dose-response relationship between weekly physical activity and diastolic blood pressure.

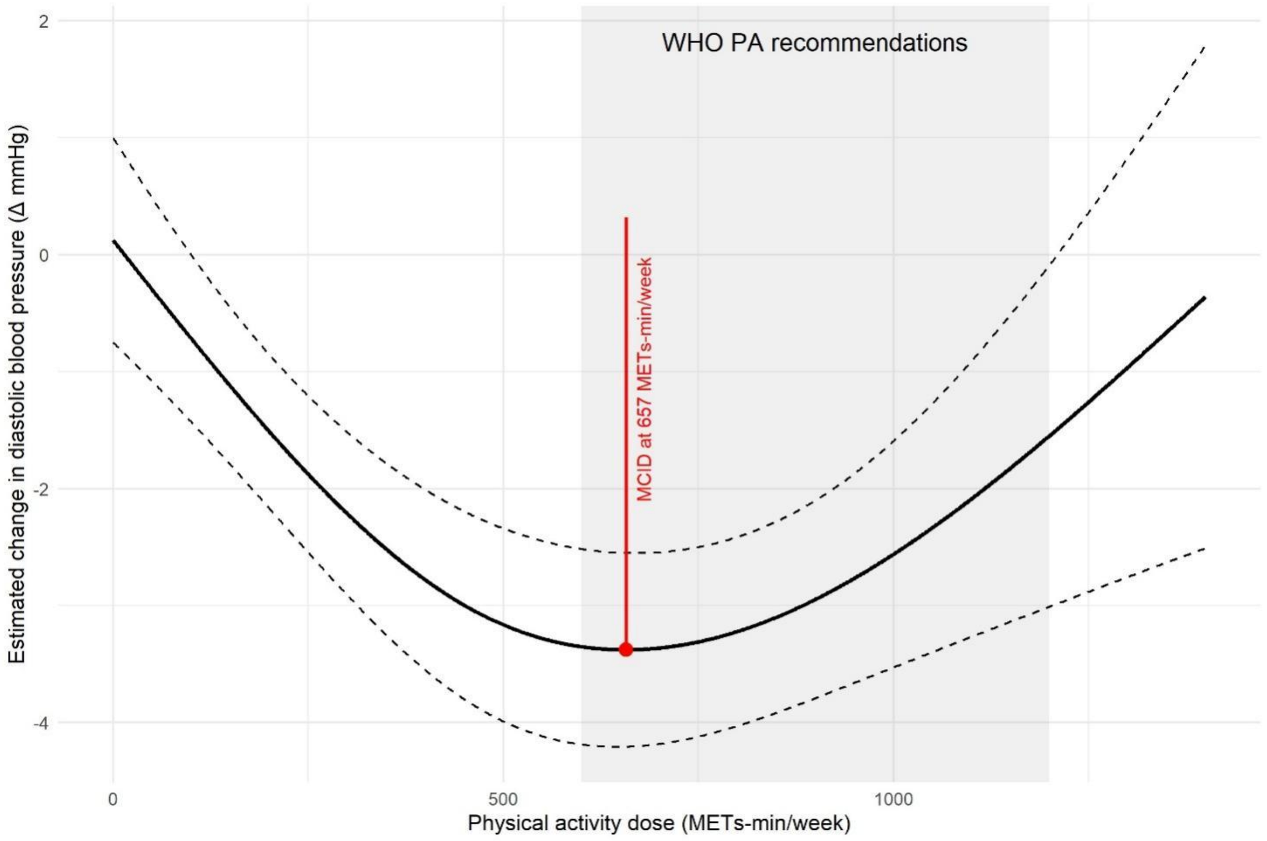

Dose-response relationship between weekly physical activity and diastolic blood pressure. Solid line represents the posterior mean estimate, and dashed lines indicate 95% credible intervals (CrI) from the Bayesian model. The vertical red line shows the predicted minimum clinically important difference (MCID) at 657 MET-min/week. The shaded area corresponds to the WHO-recommended range (600-1200 MET-min/week)

Supplementary S24. Dose response relationship of different exercises

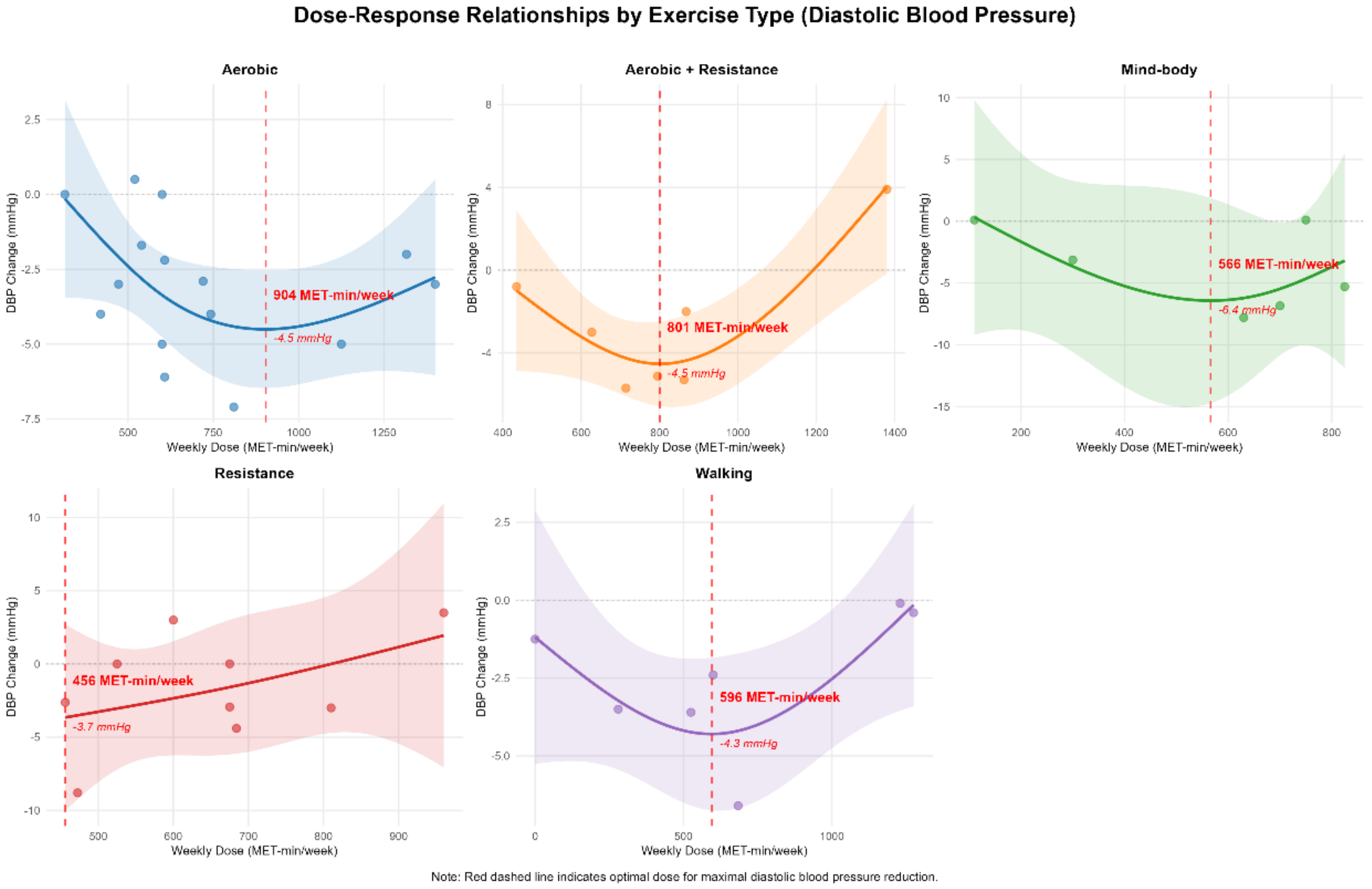

As shown in the Figure, the 95% confidence intervals for the effect sizes on diastolic blood pressure for both Mind-body and Resistance exercise encompass the null value, indicating results are not statistically significant. Therefore, we can only conclude that the optimal doses for Aerobic exercise, combined Aerobic+Resistance training, and Walking are 940 MET-min/week (associated with a reduction of -4.5 mmHg), 801 MET-min/week (-4.5 mmHg), and 596 MET-min/week (-4.3 mmHg), respectively.

Supplementary S25. Overall-level risk of bias of included studies

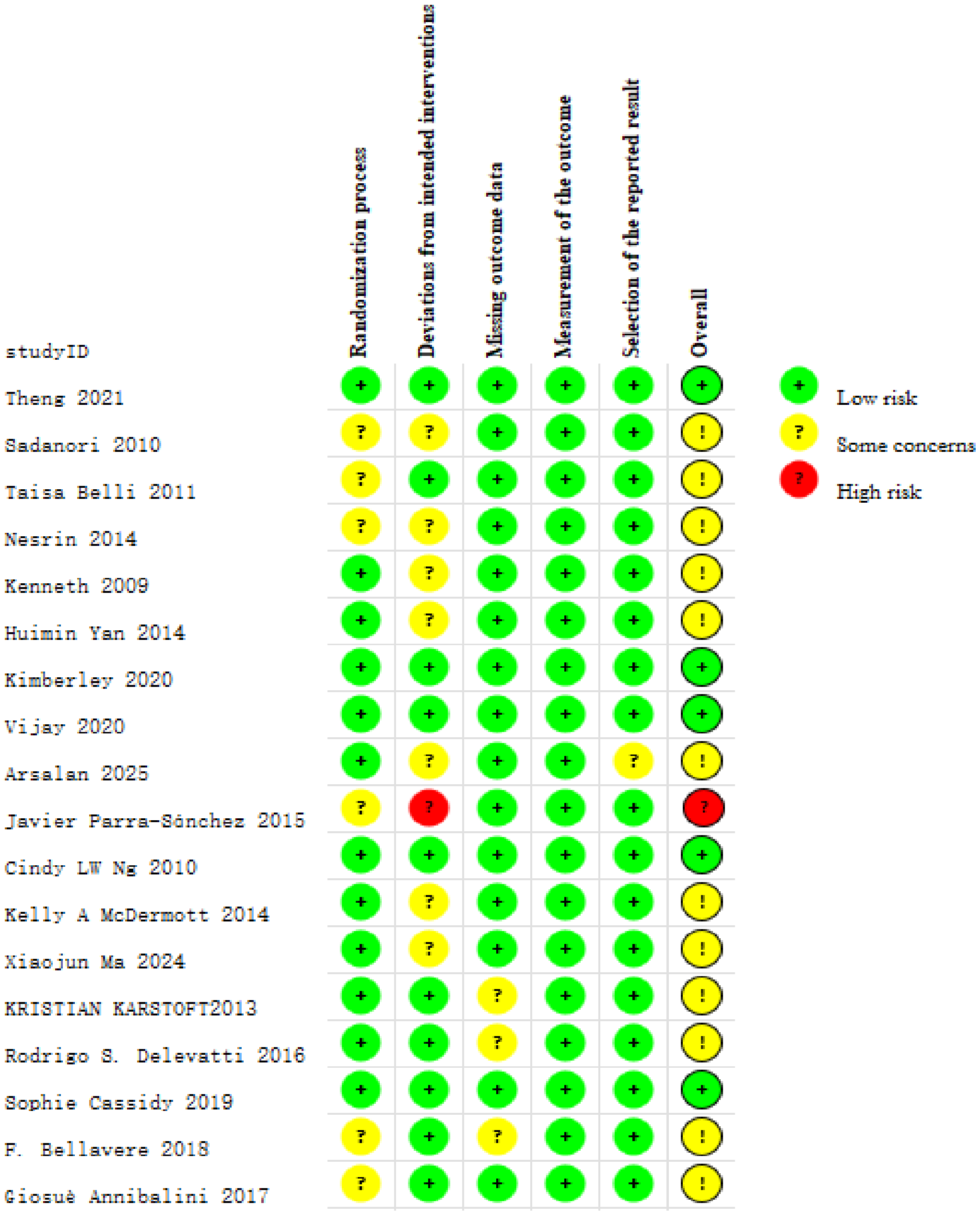
studyID
Randomization process
Deviations from intended interventions
Missing outcome data
Measurement of the outcome
Selection of the reported result
Overall
Theng 2021 + + + + + +
Sadanori 2010 ? ? + + + !
Taisa Belli 2011 ? + + + + !
Nesrin 2014 ? ? + + + !
Kenneth 2009 + ? + + + !
Huimin Yan 2014 + ? + + + !
Kimberley 2020 + + + + + +
Vijay 2020 + + + + + +
Arsalan 2025 + ? + + ? !
Javier Parra-Sánchez 2015 ? ? + + + ?
Cindy LW Ng 2010 + + + + + +
Kelly A McDermott 2014 + ? + + + !
Xiaojun Ma 2024 + ? + + + !
KRISTIAN KARSTOFT2013 + + ? + + !
Rodrigo S. Delevatti 2016 + + ? + + !
Sophie Cassidy 2019 + + + + + +
F. Bellavere 2018 ? + ? + + !
Giosuè Annibalini 2017 ? + + + + !
+ Low risk
? Some concerns
? High risk

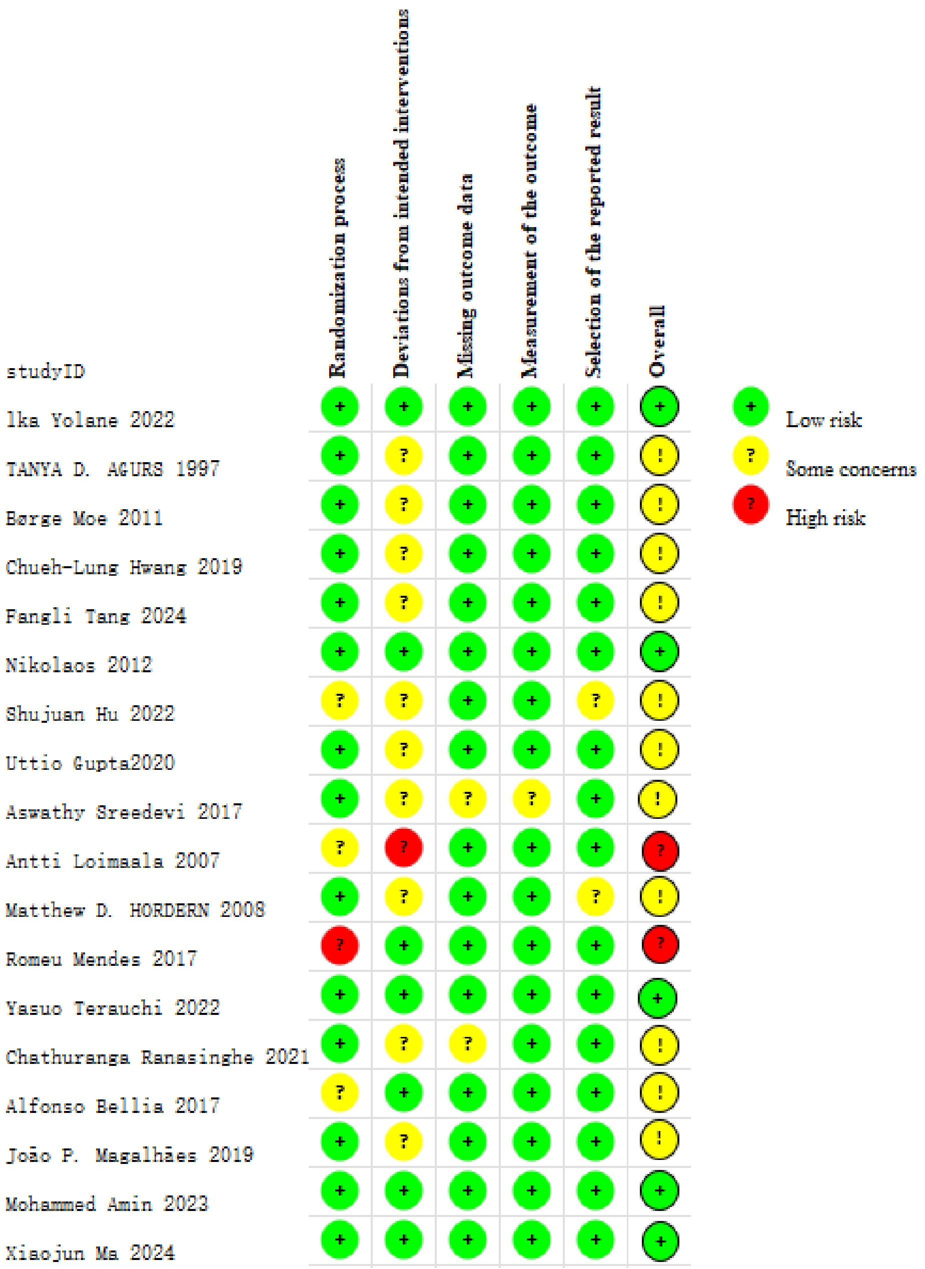

studyID
Randomization process
Deviations from intended interventions
Missing outcome data
Measurement of the outcome
Selection of the reported result
Overall
lka Yolane 2022
TANYA D. AGURS 1997
Børge Moe 2011
Chueh-Lung Hwang 2019
Fangli Tang 2024
Nikolaos 2012
Shujuan Hu 2022
Uttio Gupta2020
Aswathy Sreedevi 2017
Antti Loimaala 2007
Matthew D. HORDERN 2008
Romeu Mendes 2017
Yasuo Terauchi 2022
Chathuranga Ranasinghe 2021
Alfonso Bellia 2017
João P. Magalhães 2019
Mohammed Amin 2023
Xiaojun Ma 2024
Low risk
Some concerns
High risk

Supplementary S26. Funnel plot for the assessment of publication bias.

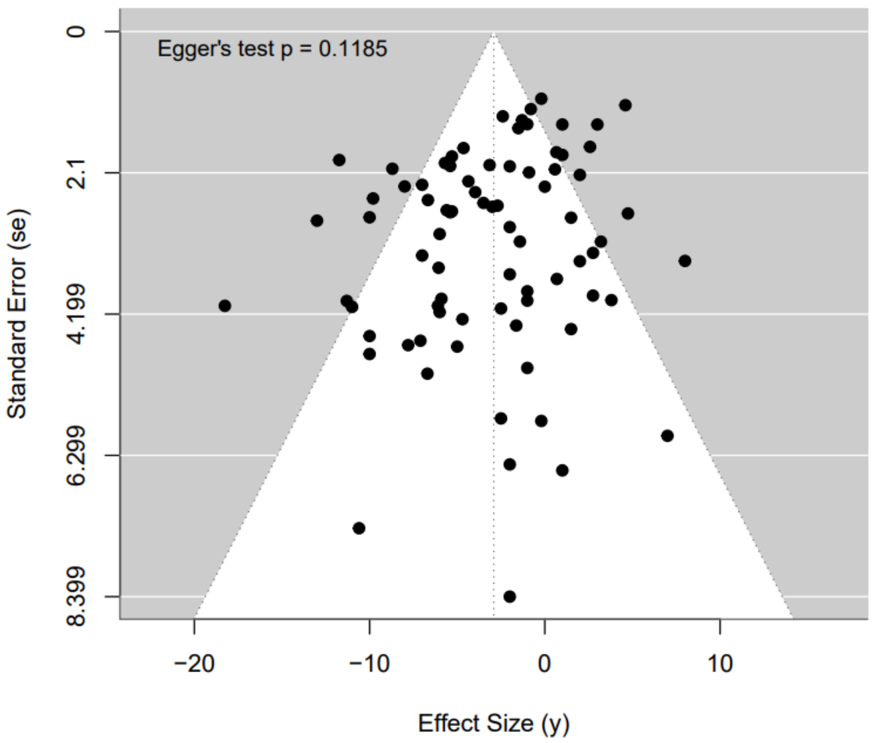

Funnel plot assessing publication bias for the effect of on systolic blood pressure.

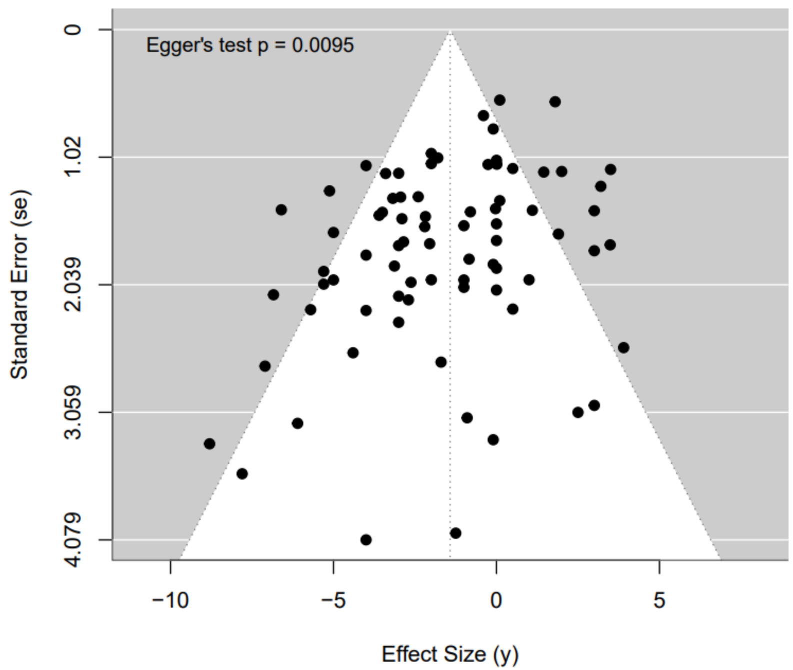

Funnel plot assessing publication bias for the effect of on diastolic blood pressure.

Supplementary S27. Grade summary of all studies

1.Systolic blood pressure

Comparison	Within-study bias	Reporting bias	Indirectness	Imprecision	Heterogeneity	Incoherence	Confidence rating	Reason(s) for downgrading
AE:BFRE	No concerns	Low risk	No concerns	Major concerns	No concerns	No concerns	Low	["Imprecision"]
AE:CG	Some concerns	Low risk	No concerns	No concerns	Major concerns	No concerns	Very low	["Within-study bias","Heterogeneity"]
AE:HIIT	Some concerns	Low risk	No concerns	Major concerns	No concerns	No concerns	Very low	["Within-study bias","Imprecision"]
AE:RT	Some concerns	Low risk	No concerns	Major concerns	No concerns	No concerns	Very low	["Within-study bias","Imprecision"]
AE:Walking	Some concerns	Low risk	No concerns	Major concerns	No concerns	No concerns	Very low	["Within-study bias","Imprecision"]
AE-RT:CG	Some concerns	Low risk	No concerns	No concerns	Major concerns	Major concerns	Very low	["Within-study bias","Heterogeneity","Incoherence"]
BFRE:CG	Some concerns	Low risk	No concerns	Major concerns	No concerns	No concerns	Very low	["Within-study bias","Imprecision"]
BFRE:RT	Some concerns	Low risk	No concerns	Major concerns	No concerns	No concerns	Very low	["Within-study bias","Imprecision"]
HIIT:CG	No concerns	Low risk	No concerns	Major concerns	No concerns	No concerns	Low	["Imprecision"]

Comparison	Within-study bias	Reporting bias	Indirectness	Imprecision	Heterogeneity	Incoherence	Confidence rating	Reason(s) for downgrading
MBE:CG	Some concerns	Low risk	No concerns	No concerns	Major concerns	No concerns	Very low	["Within-study bias","Heterogeneity"]
RT:CG	Some concerns	Low risk	No concerns	Major concerns	No concerns	No concerns	Very low	["Within-study bias","Imprecision"]
Walking:CG	Some concerns	Low risk	No concerns	No concerns	Major concerns	No concerns	Very low	["Within-study bias","Heterogeneity"]
MBE:Walking	Some concerns	Low risk	No concerns	Major concerns	No concerns	No concerns	Very low	["Within-study bias","Imprecision"]

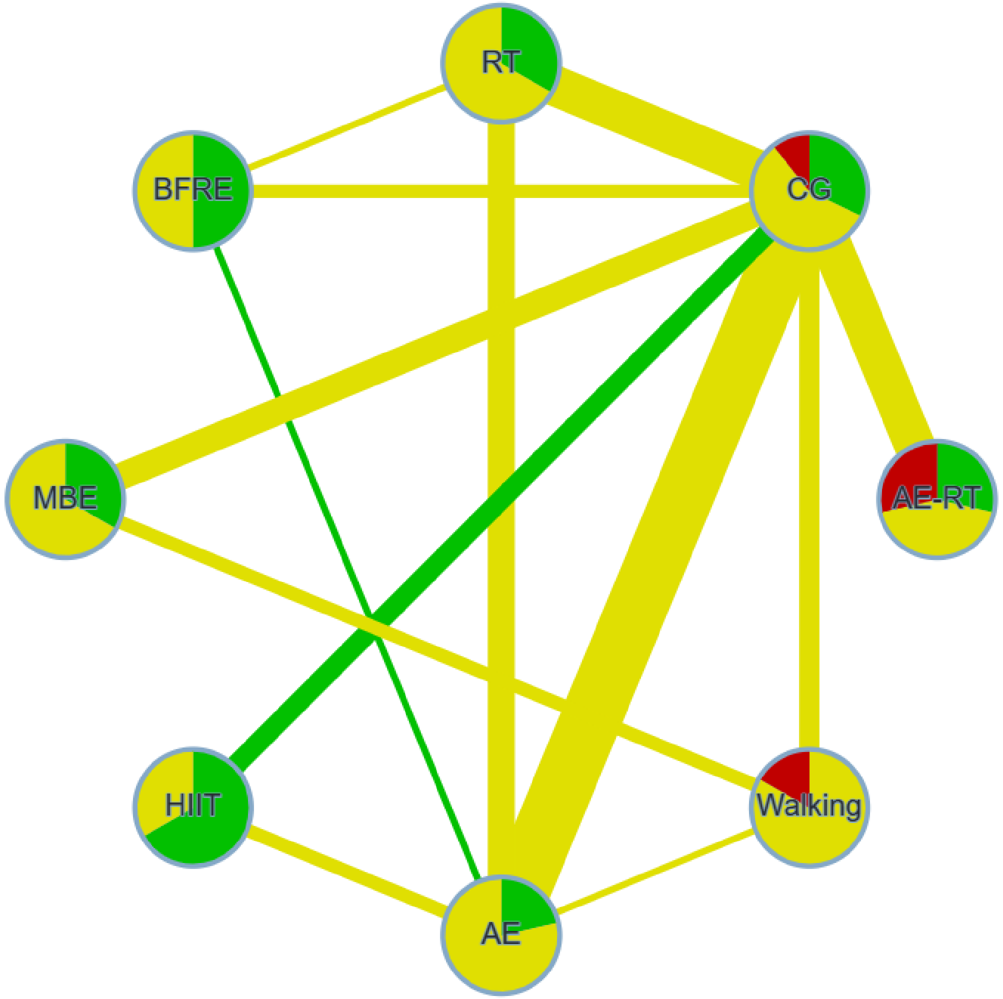

RT
BFRE
CG
MBE
AE-RT
HIIT
Walking
AE

Supplementary S27a. Network plot of study limitations of the included studies (systolic blood pressure).

.The colors in the circles indicate the percentage of low RoB studies (green), moderate RoB studies (yellow), high RoB studies (red) about each physical activity type. Edge width by the number of studies. Edge color by average RoB. The colors of the lines indicate the summative RoB assessment of each comparison. Low RoB is green, moderate RoB is yellow, high RoB is red.AE: Aerobic Exercise; AE-RT: Combined Aerobic and Resistance Training; RT: Resistance Training; MBE: Mind-body exercise;BFRE:Blood flow restriction training;HIIT:High-intensity intermittent

2.Diastolic blood pressure

Comparison	Within-study bias	Reporting bias	Indirectness	Imprecision	Heterogeneity	Incoherence	Confidence rating	Reason(s) for downgrading
AE:BFRE	Some concerns	Low risk	No concerns	Major concerns	No concerns	No concerns	Very low	["Within-study bias","Imprecision"]
**AE:CG**	**Some concerns**	**Low risk**	**No concerns**	**No concerns**	**No concerns**	**No concerns**	**Moderate**	**["Within-study bias"]**
AE:HIIT	Some concerns	Low risk	No concerns	Major concerns	No concerns	No concerns	Very low	["Within-study bias","Imprecision"]
AE:RT	Some concerns	Low risk	No concerns	No concerns	Major concerns	No concerns	Very low	["Within-study bias","Heterogeneity"]
AE:Walking	Some concerns	Low risk	No concerns	Major concerns	No concerns	No concerns	Very low	["Within-study bias","Imprecision"]

Comparison	Within-study bias	Reporting bias	Indirectness	Imprecision	Heterogeneity	Incoherence	Confidence rating	Reason(s) for downgrading
AE-RT:CG	Some concerns	Low risk	No concerns	No concerns	Major concerns	No concerns	Very low	["Within-study bias","Heterogeneity"]
BFRE:CG	Some concerns	Low risk	No concerns	No concerns	Major concerns	No concerns	Very low	["Within-study bias","Heterogeneity"]
BFRE:RT	Some concerns	Low risk	No concerns	Major concerns	No concerns	No concerns	Very low	["Within-study bias","Imprecision"]
HIIT:CG	No concerns	Low risk	No concerns	No concerns	Major concerns	No concerns	Low	["Heterogeneity"]
MBE:CG	No concerns	Low risk	No concerns	No concerns	Major concerns	No concerns	Low	["Heterogeneity"]
RT:CG	Some concerns	Low risk	No concerns	Major concerns	No concerns	No concerns	Very low	["Within-study bias","Imprecision"]
**Walking:CG**	**Some concerns**	**Low risk**	**No concerns**	**No concerns**	**No concerns**	**No concerns**	**Moderate**	**["Within-study bias"]**
MBE:Walking	Some concerns	Low risk	No concerns	Major concerns	No concerns	No concerns	Very low	["Within-study bias","Imprecision"]

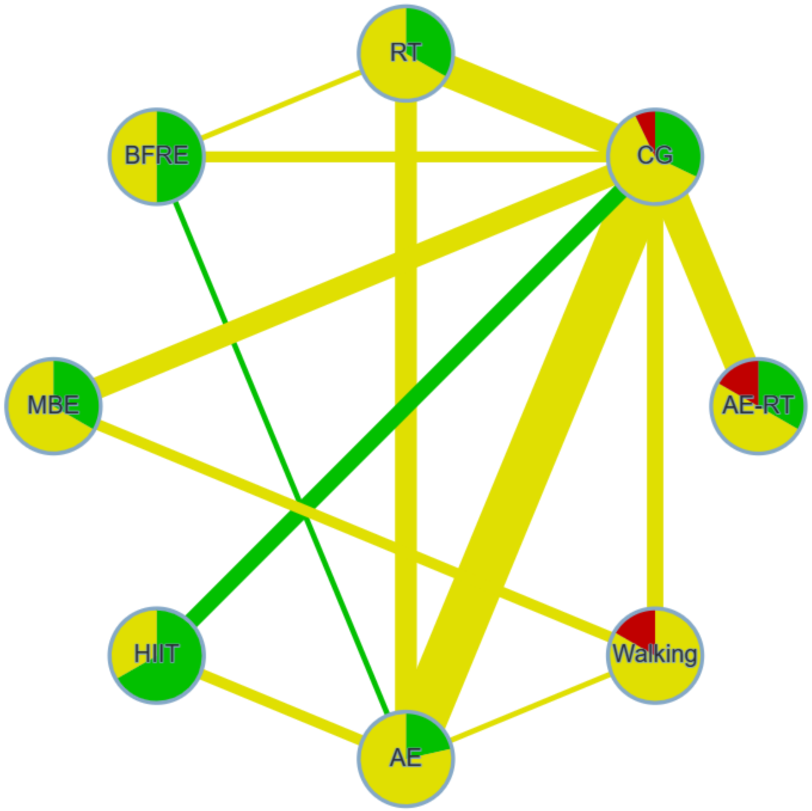

Supplementary S27b. Network plot of study limitations of the included studies (diastolic blood pressure).

.The colors in the circles indicate the percentage of low RoB studies (green), moderate RoB studies (yellow), high RoB studies (red) about each physical activity type. Edge width by the number of studies. Edge color by average RoB. The colors of the lines indicate the summative RoB assessment of each comparison. Low RoB is green, moderate RoB is yellow, high RoB is red.AE: Aerobic Exercise; AE-RT: Combined Aerobic and Resistance Training; RT: Resistance Training; MBE: Mind-body exercise;BFRE:Blood flow restriction training;HIIT:High-intensity intermitte
